# Kaemtakols A–D, highly oxidized pimarane diterpenoids with potent anti-inflammatory activity from *Kaempferia takensis*

**DOI:** 10.1007/s13659-023-00420-0

**Published:** 2023-12-01

**Authors:** Orawan Jongsomjainuk, Jutatip Boonsombat, Sanit Thongnest, Hunsa Prawat, Paratchata Batsomboon, Sitthivut Charoensutthivarakul, Saroj Ruchisansakun, Kittipong Chainok, Jitnapa Sirirak, Chulabhorn Mahidol, Somsak Ruchirawat

**Affiliations:** 1 Laboratory of Natural Products, Chulabhorn Research Institute, Bangkok, Thailand; 2 Laboratory of Medicinal Chemistry, Chulabhorn Research Institute, Bangkok, Thailand; 3 Program in Chemical Sciences, Chulabhorn Graduate Institute, Chulabhorn Royal Academy, Bangkok, Thailand; 4 Center of Excellence on Environmental Health and Toxicology (EHT), OPS, MHESI, Bangkok, Thailand; 5 Excellent Center for Drug Discovery (ECDD), School of Bioinnovation and Bio-Based Product Intelligence, and Center for Neuroscience, Faculty of Science, Mahidol University, Bangkok, Thailand; 6 Department of Plant Science, Faculty of Science, Mahidol University, Bangkok, Thailand; 7 Thammasat University Research Unit in Multifunctional Crystalline Materials and Applications (TU-MCMA), Faculty of Science and Technology, Thammasat University, Pathum Thani, Thailand; 8 Department of Chemistry, Faculty of Science, Silpakorn University, Nakhon Pathom, Thailand

**Keywords:** *Kaempferia takensis*, Diterpenoid, Structure elucidation, Anti-inflammatory, DP4+, Molecular docking

## Abstract

Four highly oxidized pimarane diterpenoids were isolated from *Kaempferia takensis* rhizomes. Kaemtakols A–C possess a tetracyclic ring with either a fused tetrahydropyran or tetrahydrofuran motif. Kaemtakol D has an unusual rearranged A/B ring spiro-bridged pimarane framework with a C-10 spirocyclic junction and an adjacent 1-methyltricyclo[3.2.1.0^2,7^]octene ring. Structural characterization was achieved using spectroscopic analysis, DP4 + and ECD calculations, as well as X-ray crystallography, and their putative biosynthetic pathways have been proposed. Kaemtakol B showed significant potency in inhibiting nitric oxide production with an IC_50_ value of 0.69 μM. Molecular docking provided some perspectives on the action of kaemtakol B on iNOS protein.

**Graphical Abstract:**

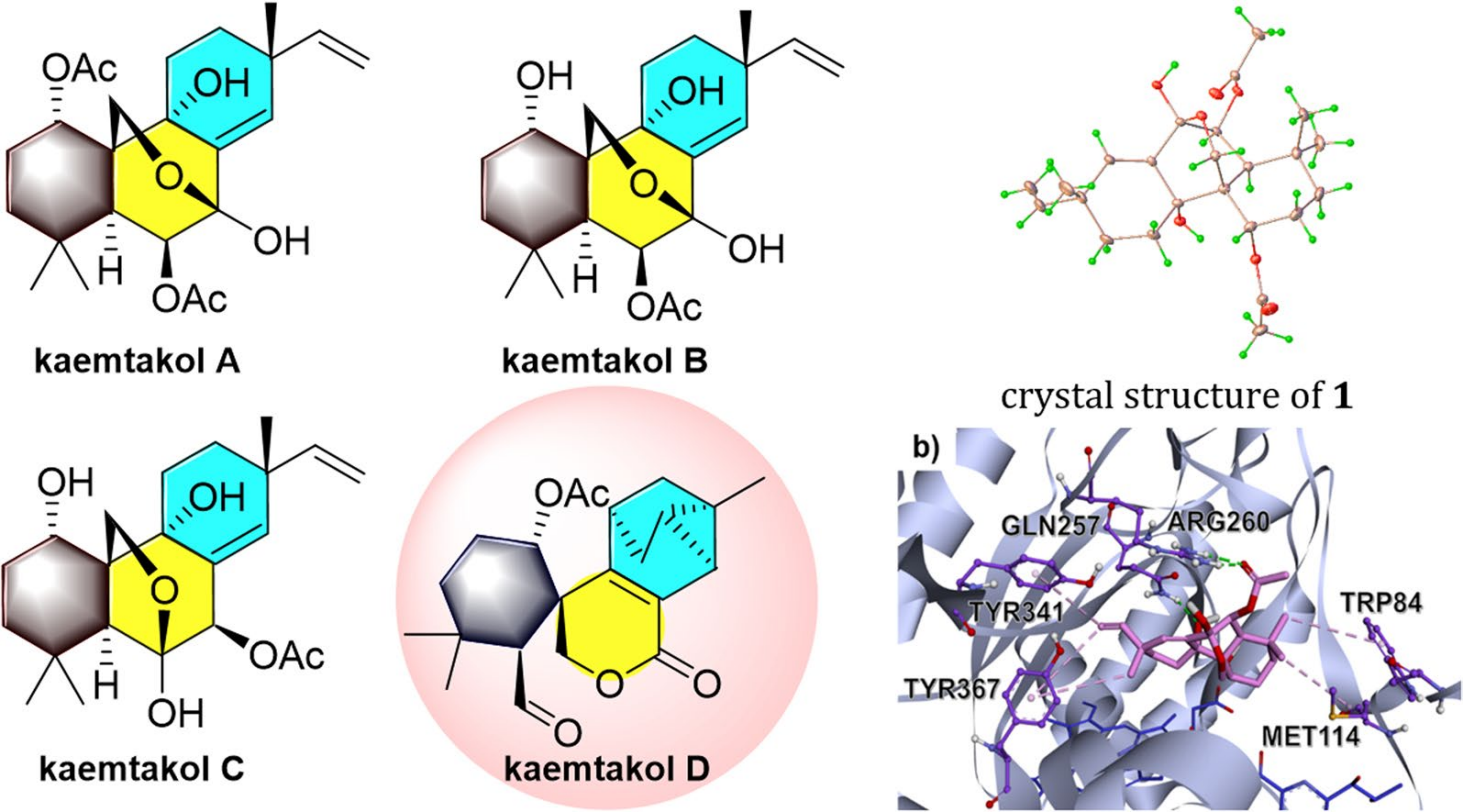

**Supplementary Information:**

The online version contains supplementary material available at 10.1007/s13659-023-00420-0.

## Introduction

The genus *Kaempferia* is a member of the Zingiberaceae family, encompassing over 60 species. This genus exhibits a broad distribution across Southeast Asia, with a significant presence in Thailand [1, 2]. The rhizomes of *Kaempferia* plants are rich in isopimarane, abietane, labdane, and clerodane type diterpenoids [3–6]. Additionally, some intriguing rearranged diterpenoids have been identified, such as unique norditerpenoids featuring an epoxide bridge with a hemiketal ring named elegansins D and E, from *K. elegans* [7].

Compounds isolated from *Kaempferia* species have gained attention due to their wide array of structural variations and remarkable anti-inflammatory effects [8–11]. Nonetheless, the scientific exploration concerning chemical characterization and biological studies has been confined to only a handful of species, such as *K. parvifera, K. galanga*, *K. marginata,* and *K. pulchra* [1]. As a result, research on plants in this genus, essentially those unexplored, could potentially unveil novel diterpenoids with intriguing structural frameworks and promising biological properties.

**Fig. 1:**
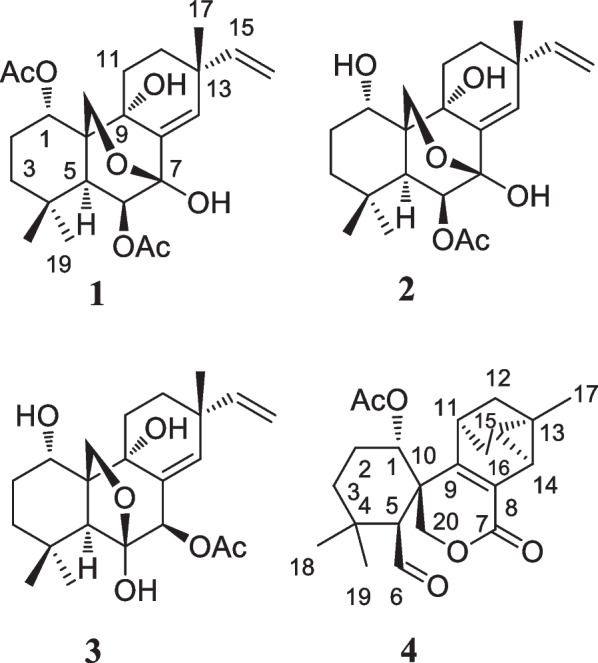
Structures of compound** 1–4**

Here, we investigated the chemical constituents from *K. takensis* Boonma & Saensouk, which to date have not yet been reported. Our investigation led to the isolation of the architecturally unique highly oxidized pimarane diterpenoids, kaemtakols A–D (**1**–**4**, Fig. 1). Compounds** 1**–**3** were characterized as C-20 oxygenated methylene pimarane-type diterpenoids with either a fused tetrahydropyran or tetrahydrofuran ring. Compound **4** featured an unusual rearranged A/B ring spiro-bridged pimarane framework with the presence of a rare spiro[5,5]decane moiety adjacent to a 1-methyltricyclo[3.2.1.0^2,7^]octene ring. All compounds were examined for their ability to reduce inflammation, by suppressing nitric oxide (NO) generation in RAW264.7 macrophage cells and by inhibiting NF-κB production in HaCat human skin cells.

## Results and discussion

**Fig. 2:**
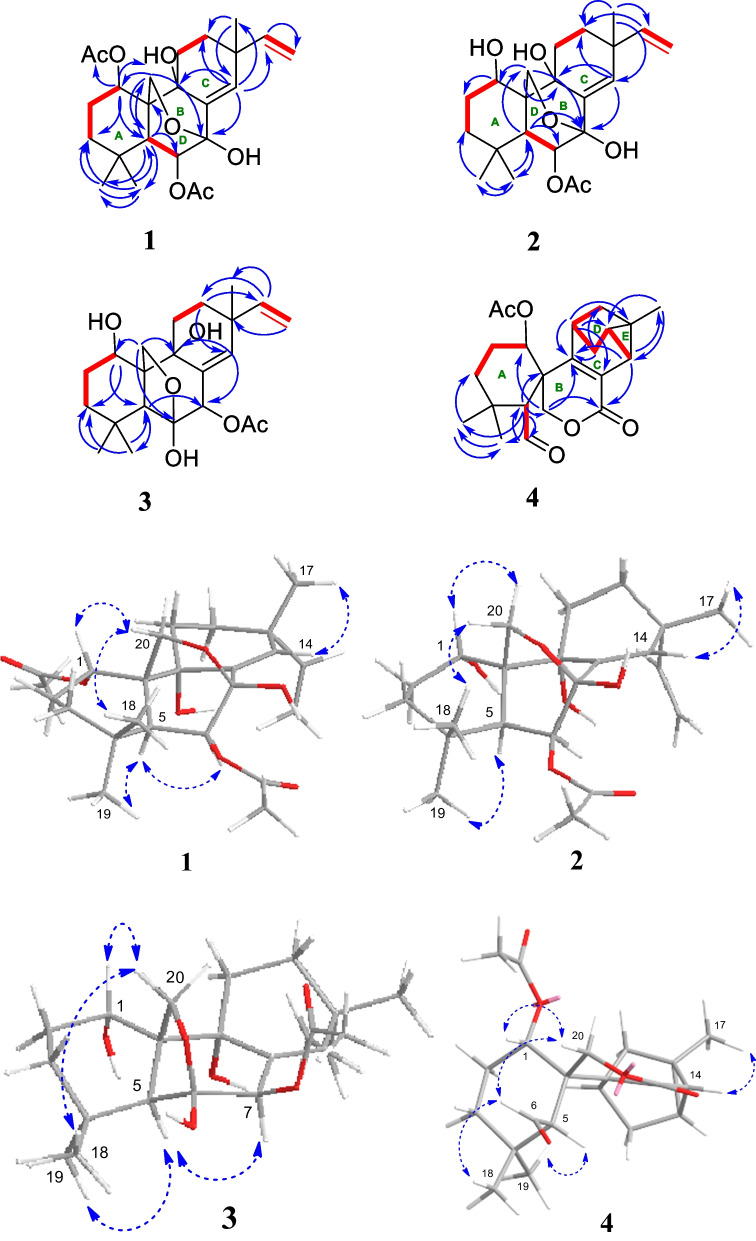
Key COSY (red dash), HMBC (blue curved arrow), and NOESY (blue dotted curve arrow) correlations of compounds **1**–**4**

**Fig. 3:**
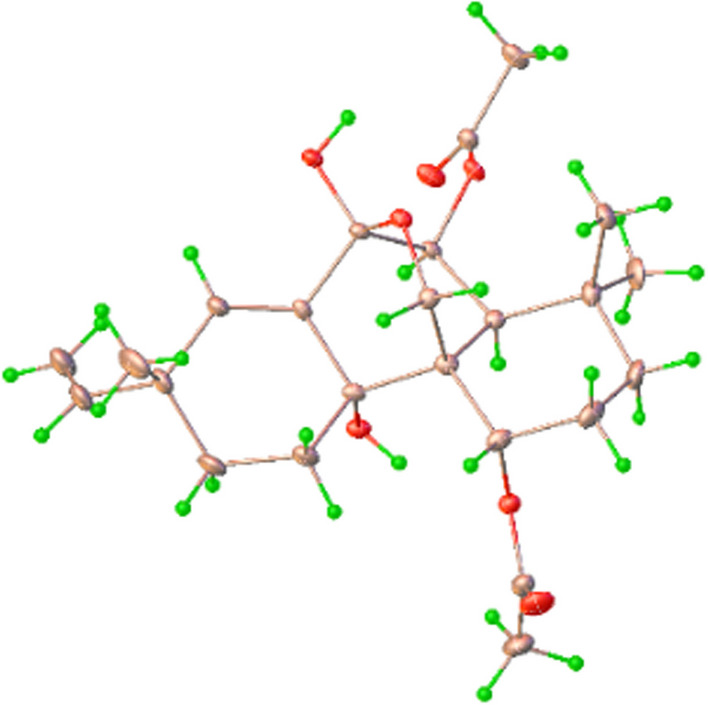
Crystal structure of compound** 1**

**Fig. 4:**
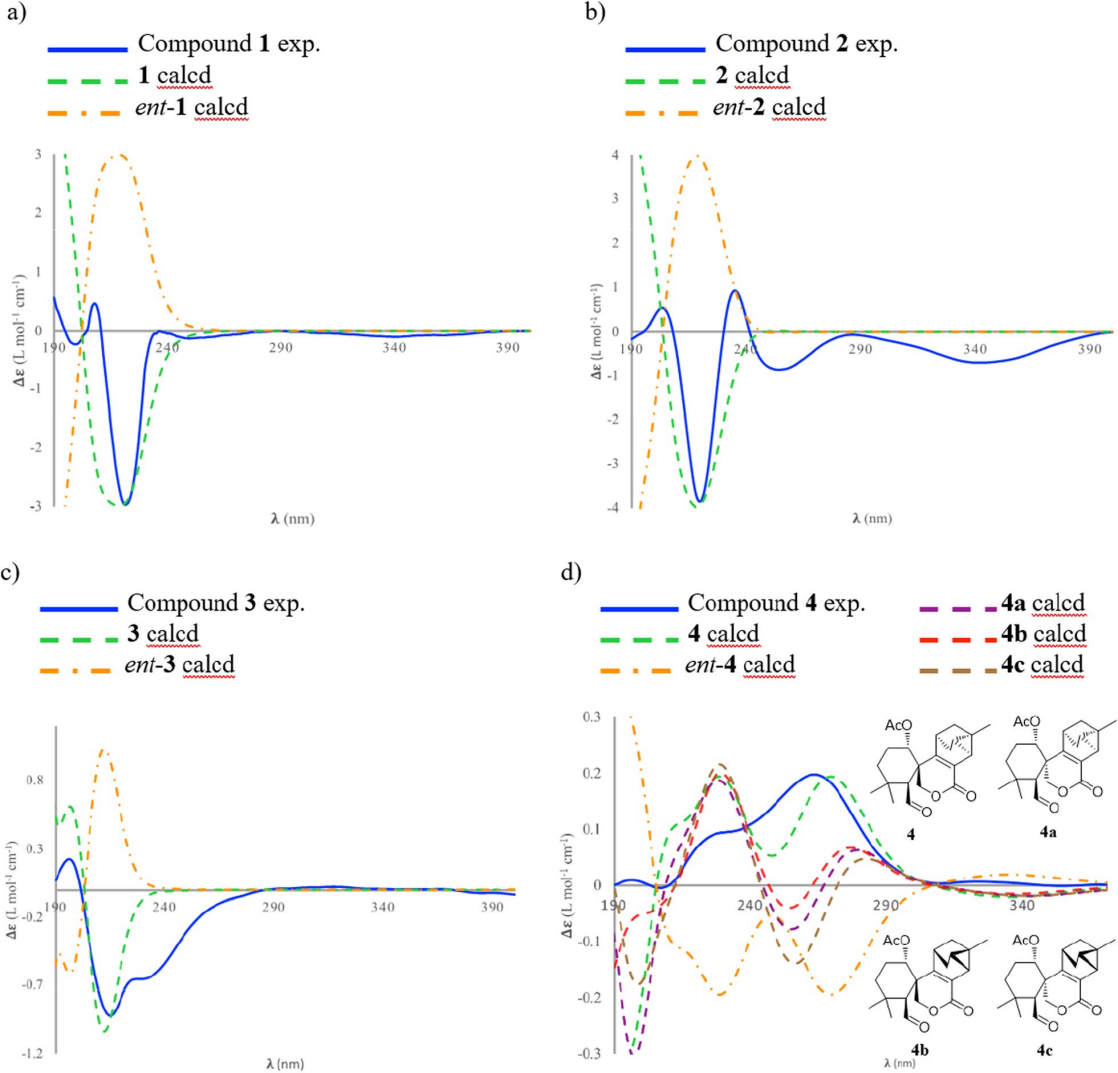
Simulated and calculated ECD spectra of **1**–**4**

Kaemtakol A (**1**), colorless crystals, has a molecular formula C_24_H_34_O_7_, deduced from the HRESIMS [M + Na]^+^ at *m/z* 457.2204 and supported by ^13^C and DEPT 135 NMR data, suggesting eight unsaturation indices. The IR absorption peaks at 3415 cm^–1^ and 1740 cm^–1^ indicated hydroxy and ester functional groups. The ^1^H- NMR (Table 1) exhibited signals for three quaternary methyls *δ*_H_/*δ*_C_ 1.09/25.4 (CH_3_-17), 1.37/22.6 (CH_3_-18), and 1.08/34.5 (CH_3_-19); two acetyl methyls at *δ*_H_ 2.12 and 2.22; an oxymethylene group at *δ*_H_/*δ*_C_ 3.53 and 3.96 (each 1H, d, *J* = 10.4 Hz)/65.8; two oxymethine groups at *δ*_H_/*δ*_C_ 4.78/71.9 and 5.64/71.7; a vinyl group with the *δ*_H_ values of 4.98 (1H, d, *J* = 10.6 Hz), 5.04 (1H, d, *J* = 17.6 Hz), and 5.86 (1H, dd, *J* = 17.5, 10.6 Hz); and an olefinic proton at *δ*_H_/*δ*_C_ 5.95/132.9. The ^13^C-NMR and DEPT of compound **1** presented twenty-four carbon signals, which matched with five methyl carbons including two acetyl methyls, six methylene carbons including one alkene and one oxygenated, five methine carbons including two oxymethines and two olefinics, and eight quaternary carbons including one alkene, two carbonyls, one oxygenated, and one hemiketal (Table 1). Excluding the above four degrees of unsaturation from two double bonds and two carbonyls, the remaining four unsaturation indices implied a tetracyclic compound. Hence, the structure of **1** was identified as C-20 oxygenated methylene pimarane diterpenoid, featuring a tetracyclic ring bearing two acetoxy groups. The COSY correlations of four spin systems comprising H-1/H_2_-2/H_2_-3, H-5/H-6, H_2_-11/H_2_-12, and H-15/H_2_-16, together with the HMBC correlations connecting H_3_-18 and H_3_-19 to C-3, C-4, and C-5; H_2_-20 to C-1, C-5, C-7, and C-9; H-1 to C-3, C-5, and C-20; H-5 to C-6, C-10, C-18, C-19, and C-20; H-14 to C-7, C-9, C-13, C-15, and C-17; and H_3_-17 to C-12 and C-14, suggested the structural architecture of **1** (Fig. 2). Based on the NOESY experiment, it was indicated that rings A and B displayed a *trans*-decalin, with the *α*-axial hydrogen on C-5 and a *β*-axial position of C-20. Additionally, the acetoxy group on C-1 was found to be in an *α*-axial position, and the oxygen linkage connecting C-7 and C-20 was situated above the B-ring plane. The C-6 relative configuration was assigned by a large coupling constant value of ^3^*J*_5,6_ ($\approx$ 10 Hz) which implied that the dihedral angle between them was nearly 0 degree, indicating a *cis* relationship [12]. This relationship was further proved by the presence of H-5 and H-6 with H_3_-19 correlations, and the absence of correlation of either H_3_-18 or H_2_-20 in the NOESY spectrum (Fig. 2). Thus, these above observations revealed the *β*-equatorial position of the OAc group on C-6. The X-ray crystallography analysis supported the assigned conformation of **1** and the visual representation of the crystal structure provided by ORTEP drawing is depicted (Fig. 3). Consequently, **1** was structurally determined as 7*α*,9*α*-dihydroxy-1*α*,6*β*-diacetoxy-7*β*,20-epoxyisopimara-8(14),15-diene. Furthermore, based on the high resemblance between the calculated and measured ECD spectrum, the (1*S*,5*S*,6*S*,7*R*,9*S*,10*S*,13*R*) absolute configuration of **1** was determined (Fig. 4).

**Table 1:** ^1^H (400 MHz) and ^13^C (100 MHz) NMR spectroscopic data of **1**–**4** in CDCl_3_

No.	**1**		**2**		**3**		**4**	
	*δ*_H_ (mult, *J* in Hz)	*δ*_C_ type	*δ*_H_ (mult, *J* in Hz)	*δ*_C_ type	*δ*_H_ (mult, *J* in Hz)	*δ*_C_ type	*δ*_H_ (mult, *J* in Hz)	*δ*_C_ type
1	4.78 br s	71.9, CH	3.77, br s	67.8, CH	4.02, br s	67.8, CH	5.13, t (2.6)	73.8, CH
2	1.79, m	23.9, CH_2_	1.78^b^, m	27.3, CH_2_	1.98, m	27.6, CH_2_	1.97, m	22.9, CH_2_
	1.76, m		1.54^*c*^, m		1.57, m			
3	1.49, m	37.5, CH_2_	1.78^b^, m	36.8, CH_2_	1.80, m	34.8, CH_2_	1.63, m	34.8, CH_2_
	1.21, m		1.14, m		1.12, m		1.35, t (3.0)	
4		33.8, C		33.9, C		31.8, C		34.0, C
5	2.97, d (10.8)	42.0, CH	2.98, d (10.7)	40.4, CH	2.62, br s	47.2, CH	3.24, d (1.9)	56.2, CH
6	5.62, d (10.6)	71.7, CH	5.50, d (10.6)	71.9, CH		107.3, C	9.93, d (2.1)	202.9, CH
7		93.8, C		93.9, C	5.47, d (2.2)	78.1, CH		163.7, C
8		135.5, C		136.1, C		131.7, C		122.7, C
9		71.7, C		72.8, C		75.6, C		154.6, C
10		44.0, C		44.0, C		54.7, C		43.5, C
11	1.93, m	25.9, CH_2_	1.92, m	25.3, CH_2_	2.10, m	27.4, CH_2_	2.84, t (5.0)	35.3, CH
	1.16, m		1.84, m		1.90^d^, m			
12	2.20, m	30.4, CH_2_	2.15, m	30.3, CH_2_	1.90^d^, m	30.4, CH_2_	1.77, ddd (11.7, 4.9, 2.1)	28.6, CH_2_
	1.53, m		1.54^*c*^, m		1.51, m		0.64, d (11.7)	
13		38.6, C		38.7, C		37.8, C		23.5, C
14	5.95, br s	132.8, CH	5.89, br s	132.8, CH	5.36, t (1.8)	132.1, CH	2.17, d (6.7)	20.9, CH
15	5.86, dd (17.5, 10.6)	146.8, CH	5.85, dd (17.4, 10.5)	147.0, CH	5.82, dd (17.5, 10.6)	147.6, CH_3_	1.30, m	21.6, CH
16a	5.04, d (17.6)	111.6, CH_2_	5.02, d (17.5)	111.4, CH_2_	4.97, d (17.5)	111.5, CH_2_	1.54, dd (11.4, 5.0)	34.8, CH_2_
16b	4.98, d (10.6)		4.96, d (10.6)		4.96, d (10.6)		0.53, d (11.4)	
17	1.09, s	25.4, CH_3_	1.08, s	25.6, CH_3_	1.09, s	23.76, CH_3_	1.32, s	17.6, CH_3_
18	1.37, s	22.6, CH_3_	1.33, s	22.7, CH_3_	1.30, s	23.76, CH_3_	1.05, s	23.6, CH_3_
19	1.08, s	34.5, CH_3_	1.02, s	34.6, CH_3_	1.23, s	33.9, CH_3_	1.28, s	33.1, CH_3_
20	3.96 d (10.4)	65.8, CH_2_	3.92, d (10.3)	66.1, CH_2_	3.71, d (8.9)	70.5, CH_2_	4.83, d (11.7)	69.7, CH_2_
	3.53, d (10.4)		3.47, d (10.2)		3.45, d (8.9)		4.59, d (11.7)	
OCOCH_3_	2.22, s	21.8^a^, CH_3_	2.18, br s	21.7, CH_3_	2.20, br s	21.0, CH_3_	2.05, br s	21.6, CH_3_
OCOCH_3_	2.12, s	21.7^a^, CH_3_						
OCOCH_3_		171.2, C		171.5, C		170.7, C		169.4, C
OCOCH_3_		169.5, C						
1-OH					2.85, d (2.0)			
6-OH					3.30, br s			
9-OH					3.96, br s			

^a^It can be interchangeable within column; ^b,c,d^Obscured due to overlapping ^1^H signals

A white amorphous compound **2** named kaemtakol B was formulated as C_22_H_32_O_6_ by HRESIMS at *m/z* of 415.2099 [M + Na]^+^. NMR analysis revealed that compounds **1** and **2** were C-20 oxygenated methylene pimarane-type diterpenoids bearing an epoxide ring (Table 1). Their NMR data closely resembled each other, with the only difference being the presence of a secondary alcohol on C-1 in **2**. This alcohol at C-1 (*δ*_C_ 67.8) position was confirmed using HMBC cross-peaks that connected H-1 (*δ*_H_ 3.77) to C-5, and H_2_-20 to C-1 and C-5. Additionally, an oxygen linkage connecting C-7 and C-20 resembling that of **1** could be established from HMBC cross-peaks connecting H-1 and H-5 to C-20; and H-5, H-14, and H_2_-20 to C-7. The above analyses enabled the establishment of the 2D structure of **2**. The NOESY experiment (Fig. 2) unveiled an identical *trans*-decalin A/B ring pattern to **1**, with the hydroxy group on C-1 occupying an *α*-equatorial position, the 7,20-oxygen bridge situated above the B-ring plane, HO-7 positioned in an *α*-orientation, and H_2_-20 in part of the 7,20-oxygen bridge located in a *β*-orientation. Consequently, **2** was structurally determined as 1*α*,7*α*,9*α*-trihydroxy-6*β*-acetoxy-7*β*,20-epoxyisopimara-8(14),15-diene. The high resemblance between the calculated and measured ECD spectra of **2** strongly suggested the (1*S*, 5*S*, 6*S*, 7*R*, 9*S*, 10*S*, 13*R*) absolute configuration (Fig. 4).

The 1D-NMR spectra of kaemtakol D (**3**) suggested the presence of a C-20 oxygenated methylene pimarane diterpenoid, and its structure appeared to resemble that of **2** (Table 1). The main differences were detected in the placement of the C-6 hemiketal hydroxy group at *δ*_C_ 107.3 and the C-7 acetoxy group at *δ*_C_ 78.1. These differences were confirmed through HMBC cross-peaks connecting H-5 and H_2_-20 to C-6, and H-5 and H-14 to C-7 (Fig. 2). NOESY interactions and the optimized structure obtained from the conformer analysis were used to determine compound **3** relative configuration (Fig. 2), which suggested the *trans*-decalin arrangement of the A/B rings having H-5 positioned in an *α*-axial orientation and C-20 in an *β*-axial orientation. As a consequence, the *β*-oriented acetoxy group at C-7, the *α*-oriented hydroxy group at C-1, and the oxygen linkage connecting C-6 and C-20 above the B-ring plane were assigned. Hence, compound **3** was determined to be 1*α*,6*α*,9*α*-trihydroxy-7*β*-acetoxy-6*β*,20-epoxyisopimara-8(14),15-diene. The high resemblance between the calculated and measured ECD spectra of **3** established the (1*S*, 5*S*, 6*S*, 7*R*, 9*R*, 10*S*, 13*R*) absolute configuration (Fig. 4).

Kaemtakol D (**4**) presented a molecular formula C_22_H_28_O_5_, deduced from the HRESIMS ion at *m/z* 395.1827 [M + Na]^+^, consistent with nine unsaturation indices. The IR absorption peaks at 3352, 1732, and 1714 cm^–1^ suggested hydroxy and carbonyl functional groups. The ^13^C-NMR data revealed twenty-two carbon signals as the resonances of four singlet methyls (one of which being an acetoxy), five methylenes (one of which being an oxymethylene carbon), six methines (including one oxymethine carbon and one sp^2^ hybridized aldehyde carbon), and seven quaternary carbons (including one olefinic carbon and two sp^2^ hybridized carbonyls). The abovementioned functional groups attributed to four out of the nine degrees of unsaturation, leaving five degrees to be deduced as a pentacyclic ring. The ^1^H-NMR spectrum exhibited four methyl protons at *δ*_H_ 2.05 (an acetyl group), 1.32, 1.28, and 1.05, oxymethylene protons at *δ*_H_ 4.83 and 4.59, an oxymethine proton at *δ*_H_ 5.13, as well as an aldehyde proton at *δ*_H_ 9.93 (Table 1). The COSY spin systems led to the assignment of three fragments: H-1/H_2_-2/H_2_-3 for C-1 to C-3, H-5/CHO for C-5 with an aldehyde function, and H-14/H-15/H_2_-16/H-11/H_2_-12 for C-11 to C-16 (Fig. 2). By deduction from HMBC correlations (Fig. 2), these fragments expanded to form the major portion of the molecules. Furthermore, the HMBC correlations were used to establish the bond connections between rings A and B, whereby the observed cross-peaks from H-5 to C-1 (*δ*_C_ 73.8), C-6 (*δ*_C_ 202.9, an aldehyde group), C-10, C-20 (*δ*_C_ 69.7), and a gem-dimethyl group, from H-6 to C-5, from a gem-dimethyl group to C-3 and C-5, and from H_2_-20 to C-1, C-5, C-7 (*δ*_C_ 163.7, an ester carbonyl group), C-9 (*δ*_C_ 154.6, an alkene group), and C-10, indicated the appearance of a six-membered carbocycle (A) with a geminal dimethyl group at C-4, an aldehyde unit at C-5, a lactone ring (B), two oxygen atoms (at C-1 and C-20), along with two quaternary carbons (at C-4, and C-10). Therefore, rings A and B are united by a bridged *spiro* lactone moiety. The incorporation of the remaining tricyclo unit to complete a pentacyclic structure was guided by the HMBC cross-peaks connecting H-11 to C-8 and C-13, H_2_-12 to C-9, and H-14 to C-9 and C-17 (Fig. 2). In addition, an isolated methylene unit that displayed an AB geminal coupling pattern of H_2_-16 (*δ*_H_ 1.54 and 0.53) was assigned to be at the junction between C-11 and C-13, and this position was further verified by long-range correlations between H-11 and C-15, H_2_-16 and C-9, H_2_-16 and C-12, as well as H_3_-17 and C-14 and C-15. Moreover, the cross-peaks from H-14 to C-7 (*δ*_C_ 163.7) signified the incorporation of the carbonyl carbon into the lactone ring, thereby necessitating the linkage of C-7 to C-8. The last remaining assignment of carbonyl carbon for the acetate group (OAc, *δ*_C_ 169.4) was positioned at C-1 due to a higher chemical shift value (*δ*_C_ 73.8) in the downfield region, compared with C-1 in compounds** 2** and **3** (67.8 ppm). The compound **4** relative stereochemistry was assigned using the NOESY correlations of H_3_-18/H-6, H_3_-19/H-5, and H-5/H-3*α* (Fig. 2). Further evidence for the *α*-configuration of the C-1 acetoxy group was provided by NOESY cross-peaks among H-1, H-6_*β*_, and H_2_-20, which inferred their co-facial relationship. The location of C-13 was deduced from strong correlations connecting H-12_*β*_ and H-14 to H_3_-17_*β*_, and the lack of correlation between H-11 and either H-14 or H-15 in the NOESY spectrum. Based on these considerations, **4** was characterized as 1*α*-acetoxy-13*β*-methyl-6,7-seco-7,20-olide-8,9-tricyclo[3.2.1.0^2,7^]octane-isopimara-8-en-6-al.

The relative configuration of the carbon positions 5, 10, 11, 13, and 14 in **4** was determined by DP4 + NMR chemical shift probability analysis. ^1^H and ^13^C chemical shifts for four possible isomers (**4** and **4a**–**4c**) were computed and compared to the experimental values (see Additional file 1). The statistical analysis confirmed the agreement with isomer **4**, with a 100% probability. Furthermore, the high resemblance between the calculated and measured ECD spectra of **4** established the (1*S*,5*S*,10*S*,11*R*,13*R*,14*S*,15*S*) absolute configuration (Fig. 4).

It is noteworthy that compound **4** is partially associated with trachylobane diterpenoids [13, 14], known for their characteristic cyclopropane moiety. However, **4** stands out from this structural family by incorporating two additional functional elements, which are the adjacent tricyclo[3.2.1.0^2,7^]octene ring system [15, 16] and a spirocyclic lactone ring junction at C-10, thereby expanding the diversity of this particular structural type.

**Fig. 5:**
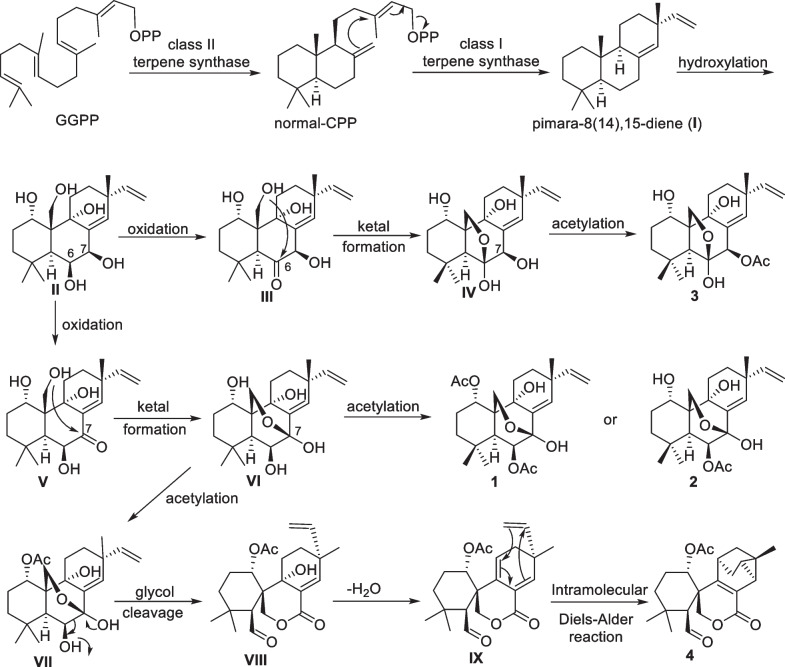
Putative Biosynthetic Pathways to Generate** 1–4**

The putative biosynthetic pathway of compounds **1**–**4** is illustrated in Fig. 5. Compounds **1**–**4** are possibly formed from geranylgeranyl pyrophosphate (GGPP) through the pimara-8(14),15-diene (**I**). Oxidation at multiple sites of **I** gives intermediate **II**, which is presumably a common biosynthetic precursor of compounds **1**–**4**. Further oxidation at C-6 of **II** followed by ketal formation gives **IV**, which is then acetylated at 7-OH to give **3**. Alternatively, oxidation at C-7 of **II** followed by ketal formation provides intermediate **VI**. Acetylation at different sites of **VI** yields compounds **1** and **2** and intermediate **VII**. Compound **4** is proposed to originate from **VII**, whereby the glycol cleavage followed by dehydration generates the diene moiety in **IX**. Upon intramolecular Diels–Alder reaction with the adjacent double bond in **IX**, compound **4** is formed.

Kaemtakols A–D (**1**–**4**) were screened for their inhibitory activity in suppressing the NO production triggered by lipopolysaccharide in RAW 264.7 macrophage cells and the NF-κB production activated by cytokine-induced inflammation in HaCat human skin cells. The results (Table 2) showed that **2** potently inhibited NO production having an IC_50_ of 0.69 μM. In contrast, substituting the acetylated group at position 1 of compound **2**, namely kaemtakol A (**1**), abolished anti-inflammation activity (IC_50_ = 103.9 μM). In addition, compounds **1**–**3** satisfyingly exhibited minimal cytotoxicity towards RAW 264.7 cells at the specific concentrations needed to inhibit NO production. Nevertheless, these compounds did not show discernible inhibitory effects against NF-*κ*B (EC_50_ > 100 μM).

**Table 2:** Inhibitory effects of the compounds **1**–**4** against the LPS-induced NO production in RAW264.7 cells and NF-κB production by cytokine induced inflammation in HaCat human skin cells

Compound	EC_50,_ (*μ*M)		% Cell viability	
	HaCaT cell	Nitric oxide^a^	At 10 μM	At 100 μM
**1**	> 100	103.9	95.66	68.47
**2**	> 100	0.69	83.02	20.80
**3**	> 100	29.87	92.54	97.19
**4**	> 100	18.81	89.70	1.55

^a^Aminoguanidine hydrochloride 500 μM, a positive control, suppresses NO 90.62%

**Fig. 6:**
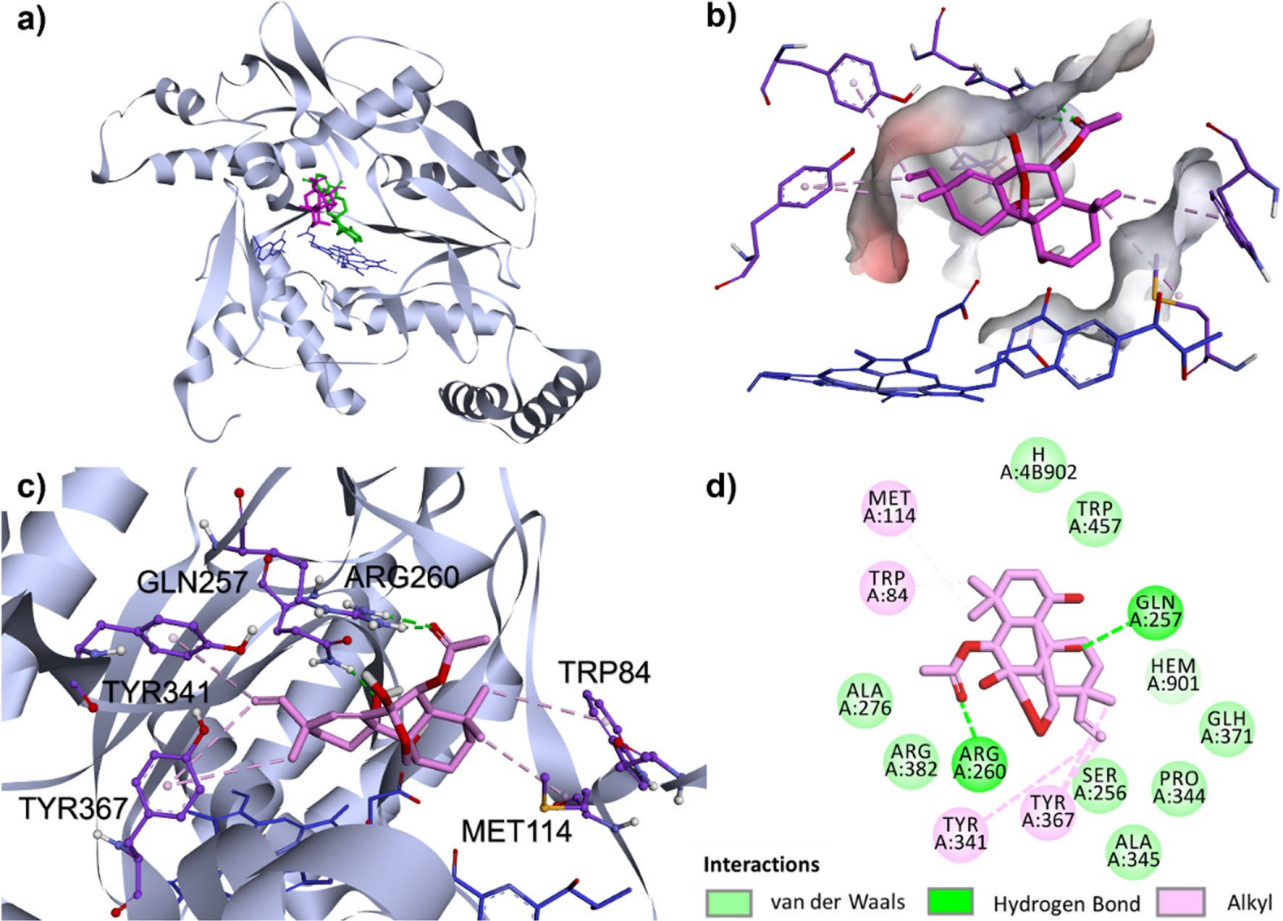
The binding position of compound **2** (pink) in **a** the whole receptor containing AR-C118901 (green) and cofactor: HEM-901 and H4B-902 (blue), and **b** the binding pocket of iNOS. **c** Potential hydrogen contacts (green dash) and hydrophobic interactions (pink dash) of compound **2** with amino acid residues in the binding site of iNOS. **d** 2D diagram representing interactions of compound **2** in the cavity of iNOS (PDB ID: 3E6T)

To determine the possible binding mode of compound **2** to iNOS, docking studies were conducted using the crystal structure with high-resolution iNOS protein (PDB 3E6T). Results (Fig. 6a, b) revealed that the binding position of **2** was next to cofactors: HEM-901 and H4B-902, and overlapped partly with that of the iNOS inhibitor AR-C118901 [17]. Compound **2** exhibited a binding energy of − 8.0 kcal/mol, which was marginally greater than that of AR-C118901 (− 9.2 kcal/mol), indicating that **2** and the cocrystal inhibitor had roughly equivalent binding affinities (Table S16). Moreover, **2** occupied the binding pocket through hydrophobic interactions and hydrogen bonds with crucial amino acid residues, including GLN257, ARG260, TYR341 and ARG260 (Fig. 6c). As depicted in Fig. 6d, the hydroxy and acetoxy groups of **2** played an important role in hydrogen contacts with GLN257 and ARG260, while its alkene and methyl groups took part in the hydrophobic interactions with MET114, TRP84, TYR341, and TYR367 in the cavity of iNOS.

## Experimental procedures

### General experimental procedures

Column gel filtration involved using Sephadex LH-20 (GE Healthcare) and MCI gel (75–150 μm; Mitsubishi Chemical, Tokyo), as well as RP-C_18_ and silica gel 60 from Merck for column chromatography separation. HPLC analysis was carried out using a Waters Delta 600 with a Waters 2996 photodiode array detector. NMR spectra were collected using Bruker Avance 400 and 600 spectrometers. HRESIMS analysis was performed with a Bruker Daltonics microTOF spectrometer. A PerkinElmer Spectrum One Spectrometer using a universal attenuated reflectance (ATR) technique was utilized to record the IR spectra. A JASCO P-1020 polarimeter was employed for recording optical rotation, and A JASCO J-810 spectropolarimeter for recording CD spectra.

### Plant materials

In April 2022, the whole plants of *Kaempferia takensis* Boonma & Saensouk were collected in Tak Province, Thailand. Assist. Prof. Dr. Saroj Ruchisansakun provided the identification. The Suan Luang Rama IX Botanic Garden in Thailand housed a reference specimen (S.R. 1851).

### Extraction and isolation

The fresh *K. takensis* rhizomes (414.6 g) were soaked in two rounds with an equal volume (1.5 L) of 95% EtOH and 50% dichloromethane-MeOH mixture at ambient temperature, respectively. The complete evaporation of the solvent gave respective crude extracts of 7.04 g and 1.53 g. Due to these two crude extracts exhibiting the same NMR patterns of diterpenoids, we decided to combine them to yield 8.6 g of a crude extract in order to get more pure active compounds.

The combined crude extract (8.6 g) was processed to the Sephadex LH-20 column using a dichloromethane-MeOH (1:1) elution, resulting in four fractions (F1–F4). F3 (2.8 g) was subjected to further separation on a Sephadex LH-20 column, and elution was carried out using a dichloromethane-MeOH (60:40) mixture to obtain four subfractions (Fr3.1–Fr3.4). Fr. 3.2 (289.4 mg) was purified using semi-preparative HPLC with a Hichrom C_18_ (250 × 21 mm i.d.). Elution was carried out with a gradient of 50:50–95:5 MeCN in H_2_O for 90 min at a flow rate of 10 mL/min, monitored at UV wavelength λ 220 nm. This process yielded 1.4 mg of kaemtakol D (**4**, *t*_R_ 25 min). Fr. 3.3 (287.0 mg) underwent purification using preparative HPLC with a YMC-Pack ODS-A C18 column (250 × 20 mm i.d.). The purification was achieved through gradient elution with 50–95% MeCN in H_2_O for 45 min at a flow rate of 10 mL/min, monitored at a UV wavelength of λ 220 nm. This process yielded 28.0 mg of kaemtakol A (**1**, *t*_R_ 16 min). Fr. 3.4 (2.3 g) was subjected to gel filtration with Sephadex LH20, using 100% MeOH as the eluent, resulting in the separation into six subfractions (Fr.3.4.1–3.4.6). Subsequently, Fr. 3.4.2 (2.0 g) underwent a second round of separation by Sephadex LH20, again using 100% MeOH as the eluent, yielding six additional subfractions (Fr.3.4.2.1–3.4.2.6). Fr. 3.4.2.3 (1.7 g) was additionally purified by semi-preparative HPLC (Hichrom C_18_ 250 × 21 mm i.d.) with 50–95% MeCN in H_2_O gradient elution over 90 min at a flow rate of 10 mL/min, monitored at UV λ 220 nm. This process yielded kaemtakol B (**2**, 140.0 mg, *t*_R_ 12 min) and kaemtakol C (**3**, 43.0 mg, *t*_R_ 14 min).

#### *Kaemtakol A* (*1*)

Colorless crystals; C_22_H_32_O_6_, mp 205–208 °C; ${\left[\alpha \right]}_{\mathrm{D}}^{24}$ +40.6 (*c* 0.59, MeOH); UV (MeOH) *λ*_max_ (log* ε*): 279 (0.838) and 212 (2.916) nm; CD (MeOH) *λ*_max_ (∆*ε*) 222 (–87.9289) nm; IR (ATR) *ν*_max_ 3415, 2953, 2869, 1740, 1608, 1372, 1227, 1026, 910, 829, and 733 cm^–1^; ^1^H (400 MHz) NMR data (CDCl_3_) and ^13^C (100 MHz) NMR data (CDCl_3_) see Table 1; ESI HRMS *m/z* 457.2204 [M + Na]^+^ (calcd for C_24_H_34_O_7_Na, 457.2197).

#### *Kaemtakol B* (*2*)

White amorphous; C_22_H_32_O_6_, ${\left[\alpha \right]}_{\mathrm{D}}^{25}$ +43.5 (*c* 0.15, MeOH); UV (MeOH) *λ*_max_ (log *ε*) 245 (0.36) nm; CD (MeOH) *λ*_max_ (∆*ε*) 343 (–11.6695), 255 (–14.299), 220 (–63.4363) nm; IR (ATR) *ν*_max_ 3413, 2935, 1734, 1365, 1235, 1023, 911, 829, and 729 cm^–1^; ^1^H (400 MHz) NMR data (CDCl_3_) and ^13^C (100 MHz) NMR data (CDCl_3_) see Table 1; ESI HRMS *m/z* 415.2099 [M + Na]^+^ (calcd for C_22_H_32_O_6_Na, 415.2091).

#### *Kaemtakol C* (*3*)

White amorphous; C_22_H_32_O_6_, ${\left[\alpha \right]}_{\mathrm{D}}^{25}$ +43.1 (*c* 0.38, MeOH); UV (MeOH) *λ*_max_ (log *ε*) 214 (0.30) nm; CD (MeOH) *λ*_max_ (∆*ε*) 225 (–10.8958) nm, 215 (–15.1665) nm, 196 (+ 3.7076) nm; IR (ATR) *ν*_max_ 3375, 2943, 1725, 1636, 1371, 1237, 1191, 1047, 960, 891, and 735 cm^–1^; ^1^H (400 MHz) NMR data (CDCl_3_) and ^13^C (100 MHz) NMR data (CDCl_3_) see Table 1; ESI HRMS *m/z* 415.2097 [M + Na]^+^ (calcd for C_22_H_32_O_6_Na, 415.2091).

#### *Kaemtakol D* (*4*)

White amorphous; C_22_H_28_O_5_, ${\left[\alpha \right]}_{\mathrm{D}}^{24}$ +182.1 (*c* 0.08, MeOH); CD (MeOH) *λ*_max_ (∆*ε*) 264 (+ 13.7087) nm, 233 (+ 6.6737) nm; IR (ATR) *ν*_max_ 3352, 2927, 2866, 1732, 1714, 1655, 1374, 1236, 1115, 1063, and 764 cm^–1^; ^1^H (400 MHz) NMR data (CDCl_3_) and ^13^C (100 MHz) NMR data (CDCl_3_) see Table 1; ESI HRMS *m/z* 395.1827 [M + Na]^+^ (calcd for C_22_H_28_O_5_Na, 395.1829).

### X-ray crystallographic analysis of 1

Colorless crystals of kaemtakol A (**1**) were obtained using the vapor diffusion method from a solution of MeOH-EtOH (1:1) and MeOH. X-ray crystallographic analyses were conducted at 150 K using a Bruker APEX-II CCD diffractometer with Mo Kα radiation. The crystallographic data was submitted to the Cambridge Crystallographic Data Center (CCDC number 2247367). The data is accessible at no cost through www.ccdc.cam.ac.uk/data_request/cif, or by emailing data_request@ccdc.cam.ac.uk, or by contacting The Cambridge Crystallographic Data Centre, 12 Union Road, Cambridge CB2 IEZ, UK, fax: + 44 1223 336,033.

### ECD calculations and DP4 + analysis

The computation methods employed in this study were slightly modified from previous reports [18]. A conformational search within a 5 kcal/mol energy window was conducted using Spartan’ 20 with the MMFF94 molecular mechanics model. The Gaussian 16 Rev. C.01 package was utilized for DFT calculations [19, 20]. All low-energy conformers were additionally optimized at the ωB97XD/cc-PVDZ level of theory with the IEFPCM solvent model (methanol). At the same computational level, the vibrational frequencies of all optimized conformers were analyzed to verify the presence of actual electronic potential energy minima, and no imaginary frequency was detected. The low-Gibbs free energy conformer with over 2% population in the Boltzmann distribution was subjected to ECD calculations. TD-DFT calculations were performed at the M06-2x/def2-SVP level with the IEFPCM solvent model (methanol) [21–23]. For each conformer, calculations were performed for 30 excited states, and the simulated ECD curves were post-processed using SpecDis. This involved applying a Boltzmann averaging approach across all conformers and employing overlapping Gaussian functions with an exponential half-width (σ = 0.35) [24, 25]. The theoretical ECD spectra of **1**–**4** of their related diastereomers and enantiomers were directly compared with experimental ECD spectra. In addition, for the same set of low-energy conformers, magnetic shielding tensors (σ) were computed using the GIAO method at the mPW1PW91/6–31 + G(d,p) level in PCM solvent modeling, and then weight-averaged. Finally, DP4 + analysis was carried out using the provided Excel tool [26]. CYLview was utilized for virtualizing all 3D structures [27].

### Molecular docking with human iNOS

Molecular docking was performed using Autodock Vina [28, 29] and AutoDockTool 1.5.6 to examine the binding of compound **2** in the cavity of iNOS oxygenase. The protein structure involved in the inhibition of nitric oxide synthase (NOS), iNOS oxygenase (PDB 3E6T), was acquired from Protein Data Bank (http://www.rcsb.org/). This structure served as the receptor. The molecular structure of **2** was obtained through geometry optimization using Gaussian09 at the B3LYP/6-311G* level. The central grid parameters were set at 70–70-70 along the x-, y- and z-axes, with a 1.000 Å spacing to encompass all protein structures. The analysis and visualization of the docking results were performed using the BIOVIA Discovery Studio Visualizer [30].

### Inhibition of NO production in LPS-activated murine macrophages (RAW 264.7 cells)

The inhibitory assay followed the methods described previously with some modifications [31, 32]. RAW 264.7 cells were seeded at 3 × 10^4^ cells/well in a 96-well flat bottom plate and cultured in DMEM high glucose supplemented with 10% FBS, 1% penicillin–streptomycin, and 1% HEPES. The plates were initially incubated at 37 °C for 24 h under 5% CO_2_ atmosphere. Thereafter, LPS (0.25 μg/mL) in the absence or presence of different concentrations of kaemtakols (100, 10, 1, 0.1, 0.01, and 0.001 μg/mL) were introduced into the culture plate and then incubated further for 24 h under the same condition. Aminoguanidine hydrochloride (iNOS inhibitor) was also added as a positive control. The nitrite accumulation in the media was determined by the Griess reaction. The 75 μL of each culture supernatant was mixed with an equivalent amount of Griess reagent and the resulting mixture was incubated at room temperature for additional 10 min. Then, the absorbance at 546 nm was measured using a multimode microplate reader (ENVISION). The cytotoxicity of kaemtakols against RAW 264.7 was determined by a standard MTT assay.

### Assessment of inhibitory effects on NF-κB nuclear translocation in human skin HaCaT cells

The assay followed the methods described previously with some modifications [33, 34]. Human skin cells (HaCaT cells) were seeded at 5 × 10^4^ cells/well in a 96-well black flat-bottom plate, using DMEM (Dulbecco’s Modified Eagle Medium) high glucose supplemented with 10% FBS and 1% penicillin–streptomycin. The plates were then incubated at 37 °C for 24 h under 5% CO_2_ atmosphere. Following the initial incubation, a range of concentrations (100, 50, 25, 12.5, 6.25, 3.125 μM) of kaemtakols were introduced to the cells, and a subsequent 1-h incubation at 37 °C with 5% CO_2_ was carried out. Afterward, a cytokine cocktail comprising TNF-α (10 ng/mL), IL-1β (10 ng/mL), and IL-6 (10 ng/mL), along with the same concentration range of kaemtakols, was introduced to the culture plates and incubated at 37 °C with 5% CO_2_ for additional 30 min. Thereafter, the treated cells were fixed with 100% methanol at -20 °C for 10 min. The fixed cells underwent triple washes with DPBS before being exposed to a blocking solution (3% BSA) at room temperature for 60 min. Subsequently, a primary antibody (anti-NF-κB), a Rabbit monoclonal antibody, was introduced into the culture plate at a 1:500 ratio in DPBS and incubated at 4 °C overnight. Afterward, the culture plate was washed with DPBS three times. A secondary antibody (Alexa Fluor 488-conjugated goat anti-rabbit IgG) at a 1:500 ratio in DPBS was added to the culture plate and incubated for 1 h at room temperature in the dark. Simultaneously, Hoechst (at a 1:1,000 ratio in DPBS) was also added to the culture plate. The translocation of NF-κB in cells treated with the cytokine cocktail and kaemtakols was subsequently detected using a high-content imaging system (Operetta). The cell viability was determined by Hoechst DNA staining (Operetta).

## Conclusions

In summary, unprecedented highly oxidized pimarane diterpenoids named kaemtakols A–D (**1**–**4**) were isolated from *K. takensis* for the first time. This discovery adds to the diversity of structural types of natural diterpenoids. Kaemtakol B displayed the highest potency in NO production inhibition, signifying its potential as a lead compound for novel anti-inflammatory agent development.

### Supplementary Information

**Additional file 1.** X-ray crystal data analyses and structure refinement for **1**. Spectra of compounds **1**–**4**, including 1D- and 2D-NMR, ESI HRMS, CD, ECD, and IR techniques. ECD calculations and DP4 + analysis. X-ray crystallographic data for compound **1**. **Table S1** DP4 + probability Excel sheets of compound **4**. **Table S2** Conformational analysis of **1**. **Table S3** Conformational analysis of **2**. **Table S4** Conformational analysis of **3**. **Table S5** Conformational analysis of **4**. **Table S6** Conformational analysis of **4a**. **Table S7** Conformational analysis of **4b**. **Table S8** Conformational analysis of **4c**. **Table S9** Coordinates of Compound **1**. **Table S10** Coordinates of Compound **2**. **Table S11** Coordinates of Compound **3**. **Table S12** Coordinates of Compound **4**. **Table S13** Coordinates of Compound **4a**. **Table S14** Coordinates of Compound **4b**. **Table S15** Coordinates of Compound **4c**. **Table S16** Summary of binding energies, amino acid residue involved in the hydrogen bond and hydrophobic interactions of **2** observed in molecular docking studies.. **Figure S1** ORTEP drawing of crystal structure of **1**. **Figure S2** 1H NMR spectrum (400 MHz) of compound **1** in CDCl3. **Figure S3** 13C NMR spectrum (100 MHz) of compound **1** in CDCl3. **Figure S4** 1H– 1H COSY spectrum of compound **1** in CDCl3. **Figure S5** HSQC spectrum of compound **1** in CDCl3. **Figure S6** HMBC spectrum of compound **1** in CDCl3. **Figure S7** NOESY spectrum of compound **1** in CDCl3. **Figure S8** HREI ( +) MS spectrum of compound **1**. **Figure S9** CD spectrum of compound **1**. **Figure S10** IR spectrum of compound **1**. **Figure S11** 1H NMR spectrum (400 MHz) of compound **2** in CDCl3. **Figure S12** 13C NMR spectrum (100 MHz) of compound **2** in CDCl3. **Figure S13** 1H– 1H COSY spectrum of compound **2** in CDCl3. **Figure S14** HSQC spectrum of compound **2** in CDCl3. **Figure S15** HMBC spectrum of compound **2** in CDCl3. **Figure S16** NOESY spectrum of compound **2** in CDCl3. **Figure S17** HREI ( +) MS spectrum of compound **2**. **Figure S18** CD spectrum of compound **2**. **Figure S19** IR spectrum of compound **2**. **Figure S20** 1H NMR spectrum (400 MHz) of compound **3** in CDCl3. **Figure S21** 13C NMR spectrum (100 MHz) of compound **3** in CDCl3. **Figure S22** 1H– 1H COSY spectrum of compound **3** in CDCl3. **Figure S23** HSQC spectrum of compound **3** in CDCl3. **Figure S24** HMBC spectrum of compound **3** in CDCl3. **Figure S25** NOESY spectrum of compound **3** in CDCl3. **Figure S26** HREI ( +) MS spectrum of compound **3**. **Figure S27** CD spectrum of compound **3**. **Figure S28** IR spectrum of compound **3**. **Figure S29** 1H NMR spectrum (400 MHz) of compound **4** in CDCl3. **Figure S30** 13C NMR spectrum (100 MHz) of compound **4** in CDCl3. **Figure S31** 1H– 1H COSY spectrum of compound **4** in CDCl3. **Figure S32** HSQC spectrum of compound **4** in CDCl3. **Figure S33** HMBC spectrum of compound **4** in CDCl3. **Figure S34** NOESY spectrum of compound **4** in CDCl3. **Figure S35** HREI ( +) MS spectrum of compound **4**. **Figure S36** CD spectrum of compound **4**. **Figure S37** IR spectrum of compound **4**.

## Data Availability

All data generated or analyzed during this study are included in this published article and its additional information files.
