# Supplementary material for: Kaemtakols A–D, highly oxidized pimarane diterpenoids with potent anti-inflammatory activity from Kaempferia takensis
Source: Nat Prod Bioprospect. 2023 Dec 1;13(1):55. doi: 10.1007/s13659-023-00420-0 (PMC10689700; doi:10.1007/s13659-023-00420-0)
Supplement: Supplementary file 1 — Additional file 1. X-ray crystal data analyses and structure refinement for 1. Spectra of compounds 1–4, including 1D- and 2D-NMR, ESI HRMS, CD, ECD, and IR techniques. ECD calculations and DP4 + analysis. X-ray crystallographic data for compound 1. Table S1 DP4 + probability Excel sheets of compound 4. Table S2 Conformational analysis of 1. Table S3 Conformational analysis of 2. Table S4 Conformational analysis of 3. Table S5 Conformational analysis of 4. Table S6 Conformational analysis of 4a. Table S7 Conformational analysis of 4b. Table S8 Conformational analysis of 4c. Table S9 Coordinates of Compound 1. Table S10 Coordinates of Compound 2. Table S11 Coordinates of Compound 3. Table S12 Coordinates of Compound 4. Table S13 Coordinates of Compound 4a. Table S14 Coordinates of Compound 4b. Table S15 Coordinates of Compound 4c. Table S16 Summary of binding energies, amino acid residue involved in the hydrogen bond and hydrophobic interactions of 2 observed in molecular docking studies.. Figure S1 ORTEP drawing of crystal structure of 1. Figure S2 1H NMR spectrum (400 MHz) of compound 1 in CDCl3. Figure S3 13C NMR spectrum (100 MHz) of compound 1 in CDCl3. Figure S4 1H– 1H COSY spectrum of compound 1 in CDCl3. Figure S5 HSQC spectrum of compound 1 in CDCl3. Figure S6 HMBC spectrum of compound 1 in CDCl3. Figure S7 NOESY spectrum of compound 1 in CDCl3. Figure S8 HREI ( +) MS spectrum of compound 1. Figure S9 CD spectrum of compound 1. Figure S10 IR spectrum of compound 1. Figure S11 1H NMR spectrum (400 MHz) of compound 2 in CDCl3. Figure S12 13C NMR spectrum (100 MHz) of compound 2 in CDCl3. Figure S13 1H– 1H COSY spectrum of compound 2 in CDCl3. Figure S14 HSQC spectrum of compound 2 in CDCl3. Figure S15 HMBC spectrum of compound 2 in CDCl3. Figure S16 NOESY spectrum of compound 2 in CDCl3. Figure S17 HREI ( +) MS spectrum of compound 2. Figure S18 CD spectrum of compound 2. Figure S19 IR spectrum of compound 2. Figure S20 1H NMR spectrum (400 MHz) of compound 3 [file 13659_2023_420_MOESM1_ESM.pdf]

Supplementary Information

Kaemtakols A–D, Highly Oxidized Pimarane Diterpenoids with Potent Anti-inflammatory Activity from *Kaempferia takensis*

Orawan Jongsomjainuk[a], Jutatip Boonsombat[a,d], Sanit Thongnest[a,d,*], Hunsa Prawat[a,d], Paratchata Batsomboon[b], Sitthivut Charoensutthivarakul[e], Saroj Ruchisansakul[f], Kittipong Chainok[g], Jitnapa Sirirak[h], Chulabhorn Mahidol[a,c], and Somsak Ruchirawat[b,c,d]

[a] Laboratory of Natural Products, Chulabhorn Research Institute, Bangkok, Thailand.

[b] Laboratory of Medicinal Chemistry, Chulabhorn Research Institute, Bangkok, Thailand.

[c] Program in Chemical Sciences, Chulabhorn Graduate Institute, Chulabhorn Royal Academy, Bangkok, Thailand.

[d] Center of Excellence on Environmental Health and Toxicology (EHT), OPS, MHESI, Thailand.

[e] Excellent Center for Drug Discovery (ECDD), School of Bioinnovation and Bio-Based Product Intelligence, and Center for Neuroscience, Faculty of Science, Mahidol University, Bangkok, Thailand.

[f] Department of Plant Science, Faculty of Science, Mahidol University, Bangkok, Thailand.

[g] Thammasat University Research Unit in Multifunctional Crystalline Materials and Applications (TU-MCMA), Faculty of Science and Technology, Thammasat University, Pathum Thani, Thailand.

[h] Department of Chemistry, Faculty of Science, Silpakorn University, Nakhon Pathom, Thailand.

* Corresponding author,

E-mail address: sanit@cri.or.th (Sanit Thongnest)

Table of Contents

X-ray crystallographic data for compound **1** 4
Table S1 DP4+ probability Excel sheets of compound **4** 5
Table S2 Conformational analysis of **1** 6
Table S3 Conformational analysis of **2** 6
Table S4 Conformational analysis of **3** 6
Table S5 Conformational analysis of **4** 7
Table S6 Conformational analysis of **4a** 7
Table S7 Conformational analysis of **4b** 7
Table S8 Conformational analysis of **4c** 7
Table S9 Coordinates of Compound **1** 9
Table S10 Coordinates of Compound **2** 10
Table S11 Coordinates of Compound **3** 13
Table S12 Coordinates of Compound **4** 15
Table S13 Coordinates of Compound **4a** 16
Table S14 Coordinates of Compound **4b** 17
Table S15 Coordinates of Compound **4c** 18
Table S16 Summary of binding energies, amino acid residue involved in the hydrogen bond and hydrophobic interactions of **2** observed in molecular docking studies. 19
Figure S1 ORTEP drawing of crystal structure of **1** 20
Figure S2 ^{1}H NMR spectrum (400 MHz) of compound **1** in $CDCl_3$ 21
Figure S3 ^{13}C NMR spectrum (100 MHz) of compound **1** in $CDCl_3$ 21
Figure S4 ^{1}H– ^{1}H COSY spectrum of compound **1** in $CDCl_3$ 22
Figure S5 HSQC spectrum of compound **1** in $CDCl_3$ 22
Figure S6 HMBC spectrum of compound **1** in $CDCl_3$ 23
Figure S7 NOESY spectrum of compound **1** in $CDCl_3$ 23
Figure S8 HREI (+) MS spectrum of compound **1** 24
Figure S9 CD spectrum of compound **1** 24
Figure S10 IR spectrum of compound **1** 25
Figure S11 ^{1}H NMR spectrum (400 MHz) of compound **2** in $CDCl_3$ 26
Figure S12 ^{13}C NMR spectrum (100 MHz) of compound **2** in $CDCl_3$ 26
Figure S13 ^{1}H– ^{1}H COSY spectrum of compound **2** in $CDCl_3$ 27
Figure S14 HSQC spectrum of compound **2** in $CDCl_3$ 27

Figure S15 HMBC spectrum of compound **2** in $CDCl_3$ 28
Figure S16 NOESY spectrum of compound **2** in $CDCl_3$ 28
Figure S17 HREI (+) MS spectrum of compound **2** 29
Figure S18 CD spectrum of compound **2** 29
Figure S19 IR spectrum of compound **2** 30
Figure S20 ^{1}H NMR spectrum (400 MHz) of compound **3** in $CDCl_3$ 31
Figure S21 ^{13}C NMR spectrum (100 MHz) of compound **3** in $CDCl_3$ 31
Figure S22 ^{1}H– ^{1}H COSY spectrum of compound **3** in $CDCl_3$ 32
Figure S23 HSQC spectrum of compound **3** in $CDCl_3$ 32
Figure S24 HMBC spectrum of compound **3** in $CDCl_3$ 33
Figure S25 NOESY spectrum of compound **3** in $CDCl_3$ 33
Figure S26 HREI (+) MS spectrum of compound **3** 34
Figure S27 CD spectrum of compound **3** 34
Figure S28 IR spectrum of compound **3** 35
Figure S29 ^{1}H NMR spectrum (400 MHz) of compound **4** in $CDCl_3$ 36
Figure S30 ^{13}C NMR spectrum (100 MHz) of compound **4** in $CDCl_3$ 36
Figure S31 ^{1}H– ^{1}H COSY spectrum of compound **4** in $CDCl_3$ 37
Figure S32 HSQC spectrum of compound **4** in $CDCl_3$ 37
Figure S33 HMBC spectrum of compound **4** in $CDCl_3$ 38
Figure S34 NOESY spectrum of compound **4** in $CDCl_3$ 38
Figure S35 HREI (+) MS spectrum of compound **4** 39
Figure S36 CD spectrum of compound **4** 39
Figure S37 IR spectrum of compound **4** 40

X-ray crystallographic data for compound 1. From a solution of MeOH/EtOH (1:1) and MeOH using the vapor diffusion method, colorless crystals were obtained for kaemtakol A (**1**), respectively. X-ray crystallographic analyses were performed on a Bruker APEX-II CCD diffractometer with Mo Kα radiation at 150 K. Crystallographic data have been deposited in the Cambridge Crystallographic Data Center (Deposition number: CCDC 2247367 for **1**). The data can be obtained free of charge *via* www.ccdc.cam.ac.uk/data_request/cif, or by emailing data_request@ccdc.cam.ac.uk, or by contacting The Cambridge Crystallographic Data Centre, 12 Union Road, Cambridge CB2 IEZ, UK, fax: +44 1223 336033.

Crystal Data and structure refinement for 1.

Empirical formula	$C_{24}H_{34}O_7$
Formula weight	434.51
Temperature/K	296
Crystal system	hexagonal
Space group	$P6_1$
a/Å	24.4225(6)
b/Å	24.4225(6)
c/Å	7.0814(3)
α/°	90
β/°	90
γ/°	120
Volume/Å^3	3657.9(2)
Z	6
ρ_{calc} g/cm^3	1.184
μ/mm^{-1}	0.086
F(000)	1404.0
Crystal size/mm^3	0.18 × 0.1 × 0.08
Radiation	MoKα (λ = 0.71073 Å)
2θ range for data collection/°	6.068 to 50.77
Index ranges	$-29 \leq h \leq 29$, $-29 \leq k \leq 29$, $-8 \leq l \leq 8$
Reflections collected	101126
Independent reflections	4471 [R_{int} = 0.0996, R_{sigma} = 0.0298]
Data/restraints/parameters	4471/152/350
Goodness-of-fit on F^2	1.241
Final R indexes [$I \geq 2\sigma(I)$]	R_1 = 0.0695, wR_2 = 0.1416
Final R indexes [all data]	R_1 = 0.0743, wR_2 = 0.1438
Largest diff. peak/hole / e Å^{-3}	0.19/-0.18
Flack parameter	-0.1(4)
CCDC No.	2247367

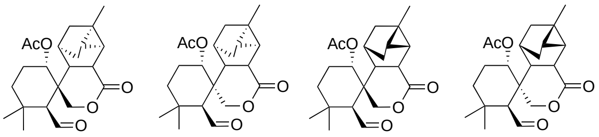

4 4a 4b 4c

Isomer	Unscaled DP4+ (%)			Scaled DP4+ (%)			DP4+ (%)		
	^{1}H	^{13}C	^{1}H+^{13}C	^{1}H	^{13}C	^{1}H+^{13}C	^{1}H	^{13}C	^{1}H+^{13}C
**4**	99.77	11.85	100.00	100.00	10.83	100.00	100.00	1.61	100.00
**4a**	0.23	0.00	0.00	0.00	0.00	0.00	0.00	0.00	0.00
**4b**	0.00	88.15	0.00	0.00	89.17	0.00	0.00	98.39	0.00
**4b**	0.00	0.00	0.00	0.00	0.00	0.00	0.00	0.00	0.00

Table S1. DP4+ probability Excel sheets of compound **4**

Settings	Type of data (shifts)
Default	Shielding tensors

Functional	Solvent?	Basis Set
mPW1PW91	PCM	6-31+G(d,p)

TMS 1H	31.560	TMS 13C	196.609
Default	μ	σ	ν
13Cu,sp2	-0.920	1.748	5.364
13Cu,sp3	2.909	1.600	6.269
1Hu,sp2	0.347	0.118	4.911
1Hu,sp3	-0.018	0.112	3.651
13Cs	-	1.557	6.227
1Hs	-	0.104	3.893

Isomer Nº			1	2	3	4	5	6	7	8
DP4+ (%)		H data	100.00%	0.00%	0.00%	0.00%	-	-	-	-
		C data	1.61%	0.00%	98.39%	0.00%	-	-	-	-
		All data	100.00%	0.00%	0.00%	0.00%	-	-	-	-
Type	sp2?	Exp	1	2	3	4	5	6	7	8
C		23.6	170.6247149	166.0710363	170.2803407	166.1648133				
C		34	159.6326916	158.7326665	159.5833846	158.6731829				
C		33.1	162.3857055	165.3667183	162.1938087	165.3556622				
C		34.8	159.4585931	161.4534847	159.2441987	161.5564827				
C		22.9	170.9776921	170.2452721	170.9176461	170.0406355				
C		73.8	121.2870154	125.9502986	121.2986244	125.9837441				
C	x	169.4	30.00567633	29.50233659	29.94643676	29.5232917				
C		21.6	173.1791204	173.3596637	173.0619562	173.2549048				
C		43.5	150.3058609	150.7436402	150.4972178	149.6114323				
C		69.7	127.2923689	125.7732972	127.1125409	126.0997097				
C	x	163.7	37.58403402	36.54194245	37.56537018	36.5265967				
C	x	122.7	73.79334	72.18400054	73.84737816	72.19256535				
C	x	154.6	39.35147054	39.41982371	39.31841577	38.88412405				
C		28.6	158.1969576	159.9276331	159.4091554	159.1315829				
C		17.6	176.634643	176.5119774	176.3068669	176.4159361				
C		20.9	171.9107406	171.2955118	171.5179898	171.1305489				
C		56.2	136.9179855	135.4693969	136.7051155	135.8655984				
C	x	202.9	-6.033386469	-8.52153859	-6.00279668	-8.74188649				
C		23.5	168.2978489	167.9089731	168.7279557	168.5331785				
C		21.6	170.4182378	169.6712606	170.2269731	169.1635508				
C		34.8	164.7061369	164.3018649	163.5504723	164.8702899				
C		35.3	157.0964555	158.016251	156.5687877	157.6556703				
H		5.13	26.3956594	25.12242106	26.43924485	25.03879107				
H		2.17	29.18979415	29.2794193	29.12170571	29.27478807				
H		3.24	28.17063527	28.25612714	28.09744689	28.23263248				
H		1.53	30.2478995	29.61722731	30.24846699	29.61319196				
H		1.63	29.68430051	30.11062334	29.66112114	30.15307149				
H		1.97	29.50667621	29.66704999	29.53082974	29.67073423				
H		1.97	29.51724894	29.77219468	29.4965086	29.77183989				
H		4.83	26.25486691	27.17309897	26.16407482	27.10886389				
H		4.59	26.86488788	26.7618616	26.90755485	26.81057884				
H		0.64	30.97449963	31.048232	30.04327428	29.99615495				
H		1.77	29.91653175	30.03283627	30.81530795	30.89007355				
H	x	9.93	21.20024172	21.25913544	21.15872619	21.30482538				
H		1.3	30.22775554	30.29404829	30.18502727	30.18727246				
H		0.53	30.84150798	30.8786278	29.59963209	29.72533874				
H		1.54	29.74282353	29.65612347	30.90177494	31.08462943				
H		2.84	28.85042375	28.81829948	28.87810566	28.82935975				
H		1.05	30.41044685	30.5082347	30.40293008	30.49165934				
H		1.28	30.12671668	30.09052347	30.06135083	30.09520624				
H		2.05	29.39710183	29.46202128	29.39387589	29.44727568				
H		1.32	30.18183034	30.1908	30.16492085	30.20592977				

Table S2 Conformational analysis of **1**

Conformers of **1**	Gibbs Free Energy (Hartree)	Gibbs Free Energy (kcal/mol)	Relative Gibbs Free Energy (kcal/mol)	Population (%)
I	-1460.769971	-916633.156803	2.294767	1.3
II	-1460.773628	-916635.451570	0.000000	61.0
III	-1460.772798	-916634.930745	0.520825	25.6
IV	-1460.771421	-916634.066678	1.384893	6.0
V	-1460.771241	-916633.953728	1.497842	5.0
VI	-1460.769776	-916633.034440	2.417130	1.1

Table S3 Conformational analysis of **2**

Conformers of **2**	Gibbs Free Energy (Hartree)	Gibbs Free Energy (kcal/mol)	Relative Gibbs Free Energy (kcal/mol)	Population (%)
I	-1308.178343	-820881.910233	0.080947	18.1
II	-1308.178305	-820881.886388	0.104793	17.4
III	-1308.177672	-820881.489180	0.502000	9.0
IV	-1308.177386	-820881.309715	0.681465	6.6
V	-1308.177650	-820881.475375	0.515805	8.7
VI	-1308.178472	-820881.991180	0.000000	20.7
VII	-1308.178413	-820881.954158	0.037022	19.5

Table S4 Conformational analysis of **3**

Conformers of **3**	Gibbs Free Energy (Hartree)	Gibbs Free Energy (kcal/mol)	Relative Gibbs Free Energy (kcal/mol)	Population (%)
I	-1308.189066	-820888.638915	0.919915	9.3
II	-1308.189359	-820888.822773	0.736058	12.6
III	-1308.188057	-820888.005768	1.553062	3.2
IV	-1308.190192	-820889.345480	0.213350	30.2
V	-1308.190532	-820889.558830	0.000000	43.1
VI	-1308.187383	-820887.582833	1.975998	1.6

Table S5 Conformational analysis of **4**

Conformers of **4**	Gibbs Free Energy (Hartree)	Gibbs Free Energy (kcal/mol)	Relative Gibbs Free Energy (kcal/mol)	Population (%)
I	-1230.634197	-772222.958618	0.000000	77.8
II	-1230.632999	-772222.206873	0.751745	22.2

Table S6 Conformational analysis of **4a**

Conformers of **4a**	Gibbs Free Energy (Hartree)	Gibbs Free Energy (kcal/mol)	Relative Gibbs Free Energy (kcal/mol)	Population (%)
I	-1230.633733	-772222.667458	0.000000	86.3
II	-1230.631981	-772221.568078	1.099380	13.7

Table S7 Conformational analysis of **4b**

Conformers of **4b**	Gibbs Free Energy (Hartree)	Gibbs Free Energy (kcal/mol)	Relative Gibbs Free Energy (kcal/mol)	Population (%)
I	-1230.633964	-772222.812410	0.000000	84.6
II	-1230.632153	-772221.676008	1.136402	12.7
II	-1230.630646	-772220.730365	2.082045	2.6

Table S8 Conformational analysis of **4c**

Conformers of **4c**	Gibbs Free Energy (Hartree)	Gibbs Free Energy (kcal/mol)	Relative Gibbs Free Energy (kcal/mol)	Population (%)
I	-1230.634108	-772222.902770	0.000000	93.9
II	-1230.631497	-772221.264368	1.638402	6.1

Table S9 Coordinates of Compound **1**

	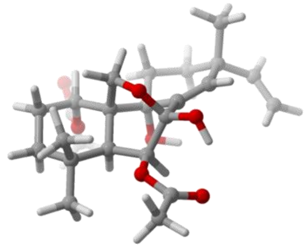 conformer **1-I**			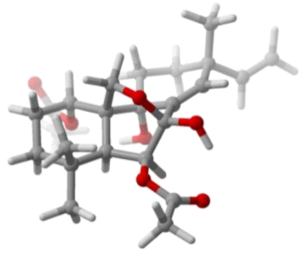 conformer **1-II**			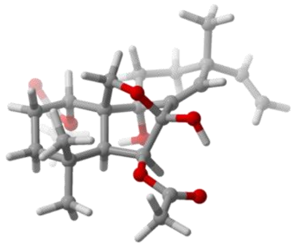 conformer **1-III**		
C	4.166741	-1.665773	1.713068	4.442720	-0.827211	1.539081	4.291352	-1.304017	1.699550
C	3.525316	-1.501101	0.318281	3.747164	-0.819612	0.169477	3.654599	-1.256774	0.293482
C	4.313423	-2.328892	-0.676259	4.561351	-1.532726	-0.893417	4.523669	-2.063040	-0.650294
C	3.822437	-3.224284	-1.532195	5.750301	-2.115370	-0.745443	4.111705	-3.004924	-1.496987
C	2.095437	-1.973887	0.428526	2.435975	-1.564979	0.321267	2.267314	-1.837980	0.412537
C	1.026174	-1.191027	0.294982	1.232164	-1.014621	0.186294	1.142205	-1.147129	0.248880
C	-0.381759	-1.637974	0.647902	-0.050085	-1.735071	0.567049	-0.222933	-1.698611	0.622719
O	-0.503157	-3.002294	0.832076	0.100682	-3.098829	0.730725	-0.240619	-3.067114	0.814117
C	-1.347406	-1.058020	-0.415072	-1.142813	-1.345524	-0.456824	-1.246280	-1.200795	-0.425559
H	-0.969115	-1.321036	-1.412224	-0.749217	-1.513730	-1.468170	-0.862067	-1.436085	-1.427019
O	-2.646827	-1.650259	-0.275580	-2.297421	-2.178543	-0.283396	-2.496359	-1.884162	-0.256708
C	-2.873713	-2.818127	-0.887248	-2.313579	-3.365867	-0.902568	-2.647696	-3.072329	-0.854943
C	-4.317644	-3.212904	-0.776926	-3.640463	-4.045669	-0.724255	-4.053364	-3.576664	-0.699418
O	-2.017308	-3.460645	-1.458548	-1.376512	-3.818685	-1.524155	-1.761299	-3.651208	-1.445892
C	-1.405425	0.494579	-0.265214	-1.499846	0.163382	-0.275542	-1.420591	0.343460	-0.277603
H	-1.196124	0.899925	-1.262396	-1.407726	0.605855	-1.275258	-1.265018	0.751971	-1.283832
C	-2.767276	1.150040	0.144328	-2.951753	0.545671	0.161697	-2.820725	0.905726	0.133085
C	-3.397656	0.640096	1.455377	-3.443484	-0.073407	1.483641	-3.396528	0.377400	1.460869
C	-3.781416	0.951497	-0.998841	-3.934324	0.154933	-0.958765	-3.831303	0.613222	-0.992537
C	-2.538417	2.676363	0.250567	-3.006382	2.088094	0.261165	-2.690748	2.445408	0.201824
C	-1.383719	3.083666	1.158917	-1.942174	2.703415	1.161494	-1.570205	2.947727	1.104015
C	-0.079747	2.444857	0.700556	-0.540125	2.318134	0.711391	-0.219747	2.386131	0.682460
H	0.745387	2.741716	1.362631	0.205763	2.735413	1.402988	0.562529	2.725316	1.376615
O	0.203325	2.941095	-0.621416	-0.284794	2.898613	-0.598680	0.120439	2.903821	-0.634586
C	1.193794	3.835638	-0.777429	0.150018	4.180299	-0.629731	0.709924	4.121454	-0.683259
C	1.413921	4.106919	-2.238702	0.427370	4.610395	-2.043772	1.056185	4.484996	-2.100753
O	1.824387	4.316169	0.133423	0.303549	4.853680	0.355972	0.932402	4.791160	0.291804
C	1.109106	0.280920	-0.062198	1.011045	0.446545	-0.148800	1.104792	0.323169	-0.117364
O	1.030190	0.433804	-1.486959	0.895285	0.548499	-1.568275	1.020893	0.408438	-1.539489
C	2.452418	0.836917	0.429560	2.235504	1.252976	0.309080	2.411021	0.986670	0.344945
C	3.593294	-0.016932	-0.123369	3.493379	0.632402	-0.298840	3.594419	0.201353	-0.220981
H	4.081260	-2.704828	2.065174	4.577401	-1.852484	1.915749	4.300284	-2.332238	2.091955
H	3.672668	-1.015510	2.450936	3.839932	-0.279568	2.278956	3.731207	-0.676849	2.409812
H	5.235518	-1.401405	1.680991	5.431745	-0.347253	1.479900	5.331299	-0.942008	1.665625
H	5.391825	-2.125351	-0.680258	4.079330	-1.555766	-1.878461	5.589133	-1.801326	-0.621396
H	2.755800	-3.461229	-1.572971	6.281022	-2.133392	0.209166	3.059296	-3.290011	-1.574116
H	4.476415	-3.755448	-2.226834	6.240787	-2.603762	-1.590174	4.817399	-3.518277	-2.153459
H	1.935375	-3.017071	0.718736	2.496966	-2.615964	0.619917	2.188310	-2.880454	0.736315
H	-0.693003	-3.394349	-0.035454	-0.000502	-3.505366	-0.144729	-0.386003	-3.476065	-0.054012
H	-4.632382	-3.173348	0.274709	-3.874374	-4.117981	0.346830	-4.746497	-2.907999	-1.230779
H	-4.455289	-4.220666	-1.181874	-3.605677	-5.042480	-1.175557	-4.332423	-3.569268	0.363063
H	-4.937752	-2.497715	-1.337250	-4.431621	-3.446565	-1.198261	-4.127385	-4.589577	-1.108173
H	-3.330132	-0.448278	1.552598	-3.186014	-1.134998	1.558775	-3.247406	-0.701703	1.571917
H	-4.460436	0.929228	1.482055	-4.538662	0.028251	1.546642	-4.476954	0.589110	1.498529
H	-2.931644	1.081704	2.346714	-3.030063	0.430992	2.367265	-2.950978	0.865024	2.338448
H	-4.135314	-0.083896	-1.059756	-4.083653	-0.929132	-1.013380	-3.421645	0.892528	-1.976901
H	-3.340602	1.225020	-1.971030	-3.576150	0.504058	-1.940922	-4.747465	1.204393	-0.833452
H	-4.657508	1.599812	-0.836352	-4.914239	0.624411	-0.775782	-4.113439	-0.444903	-1.025634
H	-3.472342	3.146345	0.599298	-4.007323	2.383426	0.615114	-3.652777	2.865823	0.536719
H	-2.341197	3.075428	-0.757681	-2.896388	2.511324	-0.751722	-2.519880	2.830946	-0.817678
H	-1.580242	2.803999	2.205071	-2.074373	2.381796	2.205788	-1.752357	2.668074	2.152859
H	-1.254334	4.176471	1.153419	-2.019133	3.799956	1.172025	-1.514228	4.045492	1.090566
H	1.677340	3.157561	-2.727090	-0.439698	4.397197	-2.683790	0.176841	4.369661	-2.749087
H	2.213122	4.844446	-2.365635	1.281830	4.039014	-2.437271	1.836360	3.802940	-2.471605
H	0.483610	4.467004	-2.699635	0.663690	5.679325	-2.062118	1.425181	5.515266	-2.135260
H	1.477302	-0.330088	-1.868789	0.580156	1.448049	-1.733007	0.819518	1.335721	-1.726908
H	2.482170	0.842926	1.529996	2.300312	1.272862	1.407982	2.459402	1.029434	1.444045
H	2.583805	1.875256	0.101192	2.148965	2.297998	-0.024222	2.457747	2.024837	-0.016540
H	4.563866	0.407733	0.175686	4.378494	1.244148	-0.067357	4.541438	0.711882	0.014229
H	3.563938	0.035665	-1.223313	3.367979	0.641966	-1.391720	3.490445	0.183908	-1.315387
C	-0.170266	0.918487	0.590856	-0.348826	0.808169	0.562111	-0.210792	0.861303	0.565269
C	-0.268128	0.276415	1.991963	-0.277548	0.138529	1.950967	-0.241252	0.217201	1.967556
H	0.715344	0.306415	2.495619	0.700102	0.351830	2.421573	0.747650	0.321611	2.451197
H	-0.978213	0.802006	2.638270	-1.051571	0.508895	2.631810	-0.975868	0.691629	2.627232
O	-0.726367	-1.062031	1.896611	-0.473492	-1.257939	1.836670	-0.601929	-1.148005	1.876648

	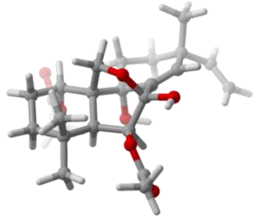 conformer **1-IV**			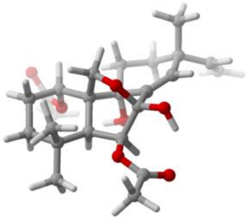 conformer **1-V**			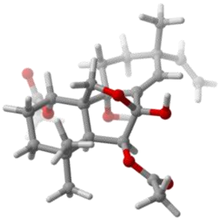 conformer **1-VI**		
C	-4.332524	-1.533869	-1.628234	4.090731	-1.651015	1.763502	-4.143766	-1.809487	-1.690775
C	-3.633455	-1.402137	-0.257834	3.505098	-1.569940	0.333704	-3.491030	-1.580613	-0.310397
C	-4.418813	-2.199010	0.763808	4.291912	-2.539322	-0.527468	-4.245140	-2.393312	0.722349
C	-3.934824	-3.119664	1.596688	5.087837	-2.249008	-1.555139	-3.718751	-3.243163	1.603494
C	-2.231221	-1.938135	-0.421684	2.071286	-2.049630	0.415686	-2.051183	-2.023359	-0.422114
C	-1.125731	-1.201851	-0.325241	1.005755	-1.273611	0.236562	-0.999419	-1.212066	-0.328490
C	0.246053	-1.687500	-0.716868	-0.403835	-1.722000	0.581283	0.418429	-1.618372	-0.671528
O	0.276132	-3.057337	-0.846283	-0.532244	-3.088086	0.746405	0.608196	-2.982062	-0.809224
C	1.270282	-1.164009	0.308990	-1.373422	-1.125027	-0.465963	1.366278	-1.041304	0.396505
H	0.926643	-1.417877	1.317766	-0.999272	-1.371495	-1.468560	0.992957	-1.325395	1.387502
O	2.480069	-1.899262	0.076386	-2.675601	-1.708640	-0.318859	2.643635	-1.641909	0.223612
C	3.038573	-2.535220	1.136413	-2.917180	-2.870710	-0.938700	2.936836	-2.699752	1.015825
C	4.259531	-3.295453	0.697379	-4.359604	-3.263720	-0.798515	4.217028	-3.340290	0.558863
O	2.612838	-2.478230	2.259434	-2.076168	-3.508446	-1.534975	2.265238	-3.056974	1.948229
C	1.402099	0.383081	0.173482	-1.425784	0.425317	-0.290011	1.392180	0.514105	0.256776
H	1.246170	0.787208	1.180982	-1.228084	0.838164	-1.286971	1.161420	0.908803	1.253504
C	2.776690	0.980010	-0.275045	-2.780740	1.090798	0.118604	2.747887	1.194211	-0.127515
C	3.344860	0.457956	-1.608260	-3.411636	0.586497	1.430599	3.405171	0.707306	-1.433647
C	3.822642	0.716881	0.825412	-3.799448	0.900364	-1.021507	3.748811	0.989119	1.025365
C	2.612686	2.516088	-0.352412	-2.528761	2.613578	0.217476	2.498532	2.717759	-0.218850
C	1.445156	2.987707	-1.212654	-1.380490	3.008449	1.138058	1.348821	3.117450	-1.137635
C	0.129052	2.400380	-0.719219	-0.075361	2.347948	0.717240	0.047372	2.454960	-0.704447
H	-0.703441	2.745488	-1.347947	0.725284	2.611197	1.423276	-0.773507	2.747614	-1.373919
O	-0.085065	2.887048	0.618723	0.316637	2.858442	-0.588195	-0.258539	2.934117	0.618063
C	-1.042694	3.806168	0.827970	0.999368	4.027040	-0.612606	-1.288575	3.781783	0.778927
C	-1.199368	4.056865	2.300806	1.384425	4.384800	-2.021599	-1.520768	4.034738	2.241236
O	-1.693753	4.320087	-0.049861	1.265018	4.662137	0.374718	-1.941339	4.237510	-0.129435
C	-1.128677	0.271888	0.045228	1.088238	0.199728	-0.107516	-1.117019	0.261370	0.018688
O	-1.004782	0.408346	1.467567	1.022853	0.311646	-1.529202	-1.070897	0.420271	1.442948
C	-2.462108	0.887127	-0.401501	2.439694	0.751321	0.371001	-2.467824	0.779273	-0.495390
C	-3.619445	0.081836	0.188795	3.559671	-0.126187	-0.190175	-3.592067	-0.082906	0.079193
H	-4.306026	-2.574456	-1.985415	4.017067	-2.674233	2.163382	-4.035946	-2.857489	-2.009665
H	-3.838468	-0.902678	-2.382552	3.545955	-0.986926	2.451965	-3.677303	-1.169479	-2.455417
H	-5.386803	-1.223418	-1.555723	5.152819	-1.361841	1.756776	-5.218891	-1.573389	-1.654047
H	-5.486049	-1.947510	0.810418	4.193249	-3.589131	-0.222822	-5.328480	-2.218477	0.732803
H	-2.878899	-3.403692	1.595216	5.239026	-1.227699	-1.911393	-2.645453	-3.449277	1.641105
H	-4.584514	-3.624321	2.314765	5.623038	-3.039496	-2.085231	-4.350032	-3.765305	2.325459
H	-2.131708	-2.988224	-0.711947	1.905625	-3.088598	0.718962	-1.876516	-3.077494	-0.661070
H	1.192069	-3.282055	-1.056391	-0.697611	-3.468110	-0.131337	0.424969	-3.176263	-1.737151
H	4.991893	-2.598818	0.264085	-4.512755	-4.257959	-1.230443	4.040484	-3.813554	-0.418058
H	3.991721	-4.017966	-0.086546	-4.994892	-2.529640	-1.315577	4.533209	-4.095425	1.286069
H	4.696632	-3.815380	1.555991	-4.641381	-3.258383	0.263304	4.999782	-2.581179	0.423342
H	3.268537	-0.631907	-1.686009	-3.353141	-0.503220	1.520592	3.384179	-0.383926	-1.520279
H	4.407529	0.740417	-1.681912	-4.471265	0.886067	1.462956	4.455614	1.039318	-1.453980
H	2.844993	0.890664	-2.484998	-2.936689	1.018399	2.321859	2.928909	1.126046	-2.330763
H	3.412695	0.911368	1.828488	-4.164201	-0.131251	-1.077331	4.112031	-0.043975	1.074382
H	4.689888	1.380979	0.680525	-3.358592	1.164122	-1.996652	3.292772	1.240867	1.996146
H	4.200096	-0.312432	0.805375	-4.667426	1.559538	-0.859485	4.620568	1.648385	0.884557
H	3.552904	2.951448	-0.728411	-3.457351	3.103491	0.552303	3.428919	3.206874	-0.550415
H	2.469456	2.906598	0.668260	-2.317779	3.002727	-0.793085	2.282277	3.101895	0.791218
H	1.593598	2.716496	-2.269028	-1.594487	2.726107	2.180126	1.563963	2.852652	-2.184185
H	1.365367	4.084958	-1.186493	-1.236746	4.098274	1.145010	1.202729	4.208098	-1.122152
H	-0.239232	4.367514	2.735579	0.503581	4.350497	-2.677245	-1.763510	3.073959	2.718087
H	-1.485667	3.108883	2.779053	2.110525	3.648860	-2.399118	-2.339169	4.750287	2.371531
H	-1.962326	4.823586	2.470477	1.834419	5.382831	-2.036534	-0.601694	4.412018	2.710540
H	-1.423513	-0.366881	1.858404	0.895557	1.254455	-1.703691	-1.422856	-0.391357	1.825806
H	-2.527770	0.898629	-1.500365	2.480197	0.786794	1.470641	-2.492395	0.755423	-1.595886
H	-2.538146	1.928673	-0.066371	2.573878	1.783472	0.014808	-2.623424	1.822904	-0.195872
H	-4.580149	0.549983	-0.075237	4.542603	0.314146	0.037423	-4.570324	0.309259	-0.238405
H	-3.547215	0.128996	1.286626	3.443604	-0.135727	-1.283569	-3.565844	0.007812	1.175930
C	0.157776	0.869768	-0.637049	-0.187218	0.829627	0.572928	0.160011	0.928051	-0.610966
C	0.185948	0.250551	-2.050389	-0.283985	0.164424	1.962453	0.282416	0.310162	-2.018883
H	-0.808736	0.335292	-2.524972	0.705079	0.180093	2.456835	-0.692831	0.336778	-2.537438
H	0.900715	0.753852	-2.709139	-0.985134	0.683883	2.624561	0.999538	0.847377	-2.647193
O	0.577415	-1.110675	-1.983971	-0.752243	-1.165517	1.841970	0.747268	-1.029495	-1.928140

Table S10 Coordinates of Compound **2**

	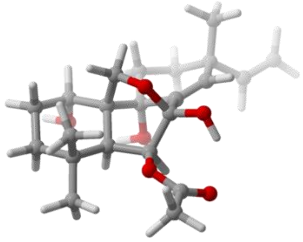 conformer **2-I**			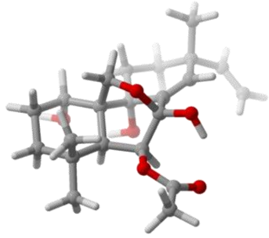 conformer **2-II**			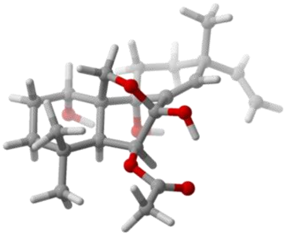 conformer **2-III**		
C	-4.496983	-0.107572	1.431198	-4.481440	-0.187234	1.563594	-4.499818	-0.107093	1.570559
C	-3.795006	0.134034	0.086405	-3.833784	0.107068	0.192993	-3.835316	0.126632	0.196426
C	-4.639358	0.963895	-0.861800	-4.819250	0.904277	-0.637200	-4.799664	0.898150	-0.681651
C	-5.856438	1.458322	-0.639253	-4.566380	2.029911	-1.302603	-4.532844	2.009496	-1.366396
C	-2.524257	0.910704	0.370451	-2.566185	0.882576	0.456215	-2.563204	0.901719	0.445888
C	-1.293529	0.449223	0.163480	-1.339219	0.425923	0.219351	-1.334916	0.432457	0.235360
C	-0.051590	1.160760	0.671828	-0.089916	1.123590	0.728579	-0.079370	1.123907	0.740813
O	-0.271257	2.471305	1.053043	-0.304733	2.423328	1.147924	-0.296934	2.422363	1.160257
C	1.062683	0.996983	-0.388591	1.008042	0.989289	-0.352730	1.016668	0.987170	-0.343067
H	0.665570	1.309413	-1.363647	0.595229	1.326324	-1.313007	0.609526	1.343267	-1.299585
O	2.173782	1.846243	-0.066882	2.124273	1.831091	-0.027384	2.139862	1.817128	-0.012432
C	2.133197	3.118157	-0.482563	2.076884	3.112923	-0.409211	2.107509	3.099664	-0.389865
C	3.422788	3.823708	-0.174855	3.376044	3.807837	-0.118204	3.423249	3.772662	-0.128030
O	1.179072	3.621506	-1.035519	1.110540	3.632255	-0.925506	1.137646	3.635036	-0.885774
C	1.492936	-0.502010	-0.452062	1.438443	-0.507009	-0.462212	1.433820	-0.511960	-0.468582
H	1.418122	-0.781994	-1.509455	1.349570	-0.758617	-1.525605	1.342316	-0.755557	-1.533269
C	2.962131	-0.877904	-0.071966	2.912838	-0.892076	-0.112207	2.908238	-0.911617	-0.129408
C	3.421685	-0.462534	1.338611	3.391786	-0.514775	1.302604	3.401439	-0.543541	1.283776
C	3.924264	-0.260240	-1.104677	3.861106	-0.247037	-1.141035	3.851072	-0.266544	-1.163567
C	3.094788	-2.410600	-0.229479	3.043475	-2.420086	-0.312280	3.024223	-2.439794	-0.337297
C	2.059436	-3.214611	0.548103	2.018611	-3.244984	0.457194	2.002402	-3.265082	0.434792
C	0.639313	-2.833834	0.141737	0.593322	-2.854426	0.079516	0.579291	-2.870113	0.054397
H	-0.084443	-3.371006	0.783481	-0.121807	-3.409304	0.715861	-0.126092	-3.414180	0.713648
O	0.401232	-3.182406	-1.227491	0.337470	-3.165654	-1.295365	0.363136	-3.285532	-1.285198
C	-0.997854	-0.928038	-0.395476	-1.051473	-0.935100	-0.382688	-1.056861	-0.937715	-0.341794
O	-0.871732	-0.811146	-1.810190	-0.944542	-0.777091	-1.794269	-0.892822	-0.860481	-1.772959
C	-2.180132	-1.857490	-0.081727	-2.227733	-1.875731	-0.078933	-2.229671	-1.875493	-0.028893
C	-3.465393	-1.212438	-0.600064	-3.523347	-1.214774	-0.549863	-3.541334	-1.234735	-0.484660
H	-4.687132	0.838046	1.961293	-4.666973	0.744825	2.118830	-4.671440	0.847386	2.090289
H	-3.869547	-0.732522	2.084325	-3.829144	-0.823520	2.181118	-3.862602	-0.732984	2.213700
H	-5.459271	-0.622296	1.285328	-5.445239	-0.704925	1.433083	-5.471630	-0.611398	1.450162
H	-4.154953	1.163156	-1.825524	-5.824825	0.467722	-0.691384	-5.800453	0.454010	-0.756094
H	-6.391178	1.301607	0.300354	-3.576868	2.494183	-1.293245	-3.550706	2.488613	-1.330183
H	-6.367071	2.048086	-1.403564	-5.343189	2.521063	-1.892436	-5.294274	2.481404	-1.990765
H	-2.640578	1.895774	0.832766	-2.667948	1.853216	0.951731	-2.663422	1.886294	0.913332
H	-0.193978	3.018144	0.255153	-0.227191	2.992601	0.365742	-0.163204	3.000813	0.391764
H	3.641841	3.734971	0.898178	3.639988	3.667666	0.938950	3.736962	3.584414	0.907535
H	3.342433	4.877114	-0.461963	3.282797	4.873411	-0.351602	3.330176	4.847555	-0.313851
H	4.247915	3.346803	-0.723704	4.178098	3.361221	-0.724063	4.190133	3.345705	-0.791254
H	4.521478	-0.503832	1.390426	4.492267	-0.555260	1.337709	4.502770	-0.563634	1.300512
H	3.045109	-1.134811	2.121495	3.027435	-1.209267	2.071773	3.065487	-1.259899	2.045728
H	3.097418	0.552430	1.591232	3.069461	0.491951	1.588334	3.067127	0.452366	1.593576
H	4.018999	0.823985	-0.978838	3.959459	0.833135	-0.986553	3.960205	0.812763	-1.007097
H	3.583067	-0.460300	-2.133292	3.505136	-0.418200	-2.169862	3.487121	-0.433754	-2.190254
H	4.926778	-0.704118	-0.993494	4.864425	-0.694949	-1.056332	4.851360	-0.722343	-1.088491
H	4.109708	-2.711585	0.077171	4.062426	-2.728583	-0.027221	4.045776	-2.752705	-0.065815
H	2.998003	-2.663172	-1.298436	2.932529	-2.644124	-1.386195	2.895810	-2.662281	-1.408995
H	2.177854	-3.072064	1.633352	2.150817	-3.130863	1.544231	2.142944	-3.164006	1.522571
H	2.204948	-4.294909	0.374409	2.162318	-4.320233	0.253284	2.128828	-4.333079	0.202255
H	-0.545787	-1.677479	-2.099777	-0.624741	-1.634870	-2.114038	-1.670757	-0.421460	-2.135853
H	-2.249747	-2.052114	1.000148	-2.276605	-2.108311	0.996621	-2.267948	-2.084144	1.050573
H	-2.024539	-2.824835	-0.581491	-2.080167	-2.825025	-0.614531	-2.080344	-2.833830	-0.545737
H	-4.320300	-1.895206	-0.479000	-4.372783	-1.906572	-0.435040	-4.381257	-1.922903	-0.303485
H	-3.333792	-1.044347	-1.679084	-3.415343	-0.998471	-1.622276	-3.511278	-1.091224	-1.578148
C	0.377033	-1.330094	0.262529	0.332557	-1.354706	0.244960	0.332645	-1.363388	0.242506
C	0.272666	-0.899314	1.738817	0.249143	-0.964605	1.733836	0.269801	-0.976469	1.732896
H	-0.690768	-1.237644	2.163845	-0.708574	-1.314026	2.162866	-0.672538	-1.339394	2.183235
H	1.068087	-1.330678	2.356902	1.052745	-1.413379	2.328531	1.091881	-1.418124	2.304787
O	0.390928	0.506709	1.853387	0.370178	0.437698	1.885266	0.372403	0.430230	1.892406
H	0.555257	-4.126948	-1.333254	0.490075	-4.106943	-1.428656	-0.110955	-2.587507	-1.760406

	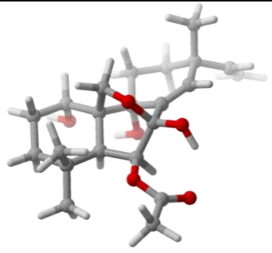 conformer **2-IV**			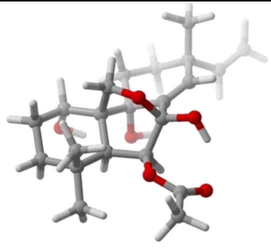 conformer **2-V**			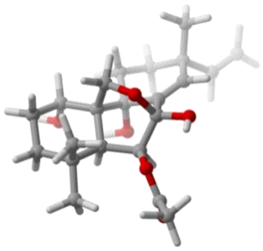 conformer **2-VI**		
C	-4.395080	0.100971	1.667863	-4.460860	-0.078920	1.454982	-4.601049	-0.022283	1.353706
C	-3.785351	0.357604	0.269247	-3.796626	0.150417	0.088316	-3.852444	0.271113	0.044785
C	-4.730768	1.288419	-0.465756	-4.672951	0.963659	-0.845512	-4.649899	1.165011	-0.885625
C	-5.441852	1.023545	-1.560374	-5.887558	1.450852	-0.597643	-5.865707	1.668728	-0.677518
C	-2.470170	1.076529	0.486580	-2.521563	0.934496	0.336286	-2.576273	1.006068	0.406508
C	-1.274433	0.552169	0.231462	-1.290034	0.463909	0.144803	-1.350777	0.523803	0.218870
C	0.019998	1.191525	0.702902	-0.044123	1.168350	0.658143	-0.107595	1.162959	0.784342
O	-0.115302	2.511018	1.092783	-0.266000	2.479755	1.032032	-0.333326	2.466670	1.159963
C	1.093312	0.969141	-0.388150	1.081613	0.993919	-0.388874	1.032104	1.029204	-0.242548
H	0.687257	1.306747	-1.351078	0.703660	1.320574	-1.367539	0.680704	1.397478	-1.212530
O	2.260466	1.752083	-0.096499	2.197509	1.830321	-0.051188	2.076270	1.905739	0.208233
C	2.282755	3.025396	-0.508498	2.180875	3.100871	-0.469111	2.598209	2.772941	-0.694175
C	3.624701	3.648772	-0.251143	3.487999	3.780100	-0.181037	3.603981	3.669862	-0.025492
O	1.341546	3.588523	-1.024872	1.230338	3.621517	-1.015010	2.298339	2.799452	-1.857832
C	1.436758	-0.551393	-0.466163	1.496240	-0.509671	-0.454747	1.442756	-0.468603	-0.373948
H	1.320504	-0.822922	-1.521893	1.434598	-0.785994	-1.513642	1.400825	-0.689104	-1.447243
C	2.891770	-1.010813	-0.124973	2.958109	-0.903566	-0.059886	2.888874	-0.897236	0.033338
C	3.410889	-0.627941	1.274129	3.410822	-0.493329	1.355114	3.312954	-0.572564	1.477412
C	3.860689	-0.445161	-1.181005	3.933724	-0.293672	-1.084929	3.905391	-0.239728	-0.920061
C	2.933365	-2.548139	-0.289309	3.073630	-2.437773	-0.217076	2.992743	-2.420144	-0.213516
C	1.874333	-3.295258	0.512839	2.025973	-3.235514	0.549404	1.913293	-3.244930	0.478292
C	0.468287	-2.833690	0.142958	0.616325	-2.847485	0.114932	0.514402	-2.807418	0.054004
H	-0.268618	-3.332279	0.800714	-0.110855	-3.369079	0.768505	-0.239313	-3.365180	0.641698
O	0.177204	-3.161449	-1.221073	0.437844	-3.303008	-1.217356	0.309460	-3.067007	-1.339458
C	-1.072234	-0.835338	-0.343341	-0.998965	-0.921719	-0.388789	-1.063800	-0.829732	-0.405670
O	-0.975724	-0.714631	-1.759844	-0.785504	-0.883524	-1.815761	-0.898047	-0.634111	-1.806578
C	-2.296548	-1.700235	-0.006406	-2.183582	-1.849080	-0.092397	-2.274953	-1.746602	-0.176221
C	-3.557557	-0.970171	-0.471133	-3.482530	-1.207926	-0.583727	-3.529286	-1.046277	-0.698803
H	-4.522636	1.043376	2.222949	-4.640158	0.872971	1.976780	-4.787848	0.899295	1.925788
H	-3.742785	-0.551123	2.268819	-3.812554	-0.691657	2.098592	-4.007727	-0.694003	1.992167
H	-5.382761	-0.375530	1.571178	-5.424061	-0.599566	1.341302	-5.569140	-0.506655	1.152601
H	-4.838635	2.275899	0.001270	-4.219614	1.161319	-1.825527	-4.129426	1.405618	-1.820565
H	-5.389331	0.062717	-2.076947	-6.396964	1.300020	0.356601	-6.434482	1.472531	0.234311
H	-6.110167	1.775320	-1.985402	-6.421353	2.028926	-1.354701	-6.340571	2.306677	-1.426031
H	-2.509918	2.063983	0.958137	-2.633393	1.930978	0.774624	-2.690570	1.974401	0.902303
H	-0.019919	3.055530	0.295004	-0.120210	3.032586	0.247178	0.524522	2.810427	1.442000
H	3.897968	3.513397	0.804237	3.757557	3.632780	0.873487	3.112426	4.260953	0.760604
H	3.589698	4.713309	-0.504455	3.407087	4.847026	-0.412765	4.051740	4.337131	-0.769116
H	4.389130	3.144847	-0.860739	4.280001	3.324830	-0.793902	4.381670	3.061930	0.458760
H	3.019856	-1.283295	2.064264	3.046373	-1.182309	2.129109	4.407108	-0.670857	1.565134
H	3.148704	0.401486	1.539788	3.074875	0.514395	1.621875	2.875907	-1.256075	2.217433
H	4.507859	-0.728585	1.295937	4.510952	-0.520271	1.406108	3.029800	0.447520	1.759911
H	3.481446	-0.620081	-2.200850	4.041547	0.789762	-0.958998	4.116285	0.801467	-0.649302
H	4.838185	-0.947250	-1.097947	3.600057	-0.492012	-2.116349	3.555455	-0.255806	-1.963612
H	4.021900	0.631073	-1.054128	4.929745	-0.749392	-0.965716	4.863075	-0.782913	-0.874457
H	3.937139	-2.907011	-0.009454	4.085383	-2.745710	0.093928	3.988923	-2.765265	0.108036
H	2.795157	-2.790925	-1.355949	2.975884	-2.692807	-1.284782	2.928406	-2.602990	-1.299038
H	2.027673	-3.162018	1.594956	2.135099	-3.101763	1.637230	1.999823	-3.171825	1.573575
H	1.954654	-4.381524	0.334111	2.154426	-4.310540	0.353229	2.041159	-4.315686	0.242652
H	-0.706575	-1.595452	-2.063353	-1.564993	-0.485919	-2.219484	-0.576492	-1.486408	-2.139113
H	-2.341348	-1.913898	1.073199	-2.247496	-2.044964	0.988197	-2.382229	-1.996532	0.891115
H	-2.212964	-2.664463	-0.528448	-2.025698	-2.814707	-0.592701	-2.124376	-2.689166	-0.722682
H	-4.441894	-1.615665	-0.354622	-4.330094	-1.891762	-0.427036	-4.401962	-1.714519	-0.638763
H	-3.432286	-0.766979	-1.544317	-3.423280	-1.070161	-1.678214	-3.359896	-0.826741	-1.763120
C	0.294924	-1.318167	0.275522	0.370797	-1.334841	0.249670	0.284995	-1.307666	0.259399
C	0.255361	-0.888524	1.755292	0.265415	-0.901509	1.724722	0.151598	-0.967033	1.755838
H	-0.714243	-1.173092	2.205163	-0.692753	-1.245229	2.156689	-0.829060	-1.310481	2.135350
H	1.041212	-1.367639	2.350074	1.066850	-1.329163	2.335184	0.921309	-1.452813	2.366119
O	0.457184	0.508131	1.870520	0.372371	0.508803	1.843064	0.288782	0.427705	1.952832
H	0.273844	-4.112793	-1.333593	-0.009462	-2.613470	-1.729367	0.445014	-4.006836	-1.498780

	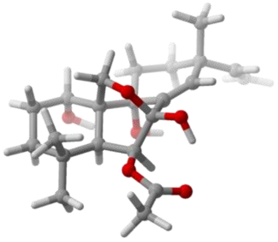 conformer **2-VII**

C	-4.385029	0.179564	1.679243
C	-3.784666	0.381708	0.267257
C	-4.728391	1.288836	-0.499177
C	-5.477354	0.974829	-1.555402
C	-2.465077	1.103620	0.456028
C	-1.268241	0.565443	0.229501
C	0.031639	1.198769	0.697725
O	-0.105851	2.519675	1.079069
C	1.109333	0.965338	-0.387490
H	0.719228	1.319782	-1.351655
O	2.283428	1.733308	-0.084146
C	2.329083	3.004595	-0.496496
C	3.686653	3.599832	-0.260986
O	1.391380	3.586499	-1.001581
C	1.434794	-0.559247	-0.472316
H	1.319545	-0.827487	-1.528680
C	2.885521	-1.035805	-0.131021
C	3.409344	-0.658946	1.269290
C	3.856915	-0.477087	-1.188788
C	2.909833	-2.573467	-0.296399
C	1.847619	-3.315107	0.505661
C	0.447363	-2.846362	0.124382
H	-0.283741	-3.329388	0.802849
O	0.196453	-3.284412	-1.202047
C	-1.075894	-0.832684	-0.312632
O	-0.919928	-0.801275	-1.746880
C	-2.298467	-1.693213	0.030228
C	-3.575522	-0.980224	-0.418183
H	-4.500586	1.142451	2.199959
H	-3.730735	-0.454988	2.296288
H	-5.376474	-0.292776	1.607314
H	-4.800751	2.304981	-0.091307
H	-5.471735	-0.019190	-2.008763
H	-6.140672	1.715341	-2.006839
H	-2.502180	2.107021	0.892849
H	0.061262	3.066048	0.293582
H	4.000223	3.412420	0.774724
H	3.658185	4.674552	-0.467746
H	4.417895	3.113527	-0.923685
H	4.509149	-0.720913	1.275291
H	3.054866	-1.347745	2.048265
H	3.116407	0.354502	1.563802
H	4.032699	0.597360	-1.062753
H	3.474959	-0.649460	-2.208062
H	4.828370	-0.990668	-1.108001
H	3.913423	-2.938199	-0.022579
H	2.759896	-2.817929	-1.360463
H	2.003405	-3.192256	1.589165
H	1.908490	-4.394746	0.302378
H	-1.661592	-0.307127	-2.114285
H	-2.332402	-1.886908	1.112599
H	-2.213329	-2.665246	-0.475020
H	-4.450619	-1.623331	-0.240257
H	-3.530471	-0.838172	-1.511056
C	0.292262	-1.322974	0.271874
C	0.267585	-0.889378	1.750532
H	-0.689427	-1.183872	2.219670
H	1.069285	-1.360135	2.328143
O	0.452510	0.513538	1.866846
H	-0.242327	-2.574341	-1.692804

Table S11 Coordinates of Compound **3**

	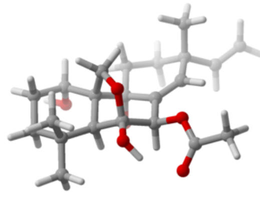 conformer **3-I**			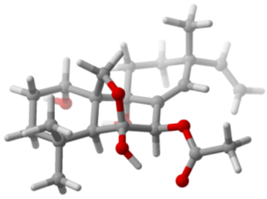 conformer **3-II**			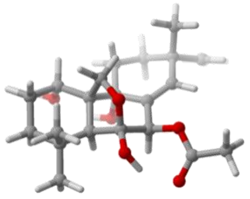 conformer **3-III**		
O	1.613090	2.480084	0.368250	1.405491	2.561241	0.362683	1.720187	2.430916	0.304233
C	0.995366	1.240805	0.317107	0.881101	1.279037	0.326774	1.043167	1.221890	0.294782
C	-0.384281	1.334564	-0.384727	-0.524213	1.269492	-0.328562	-0.353965	1.369844	-0.363014
H	-0.215393	1.542453	-1.453419	-0.406617	1.488144	-1.401916	-0.213172	1.546258	-1.441261
O	-1.171839	2.399414	0.168489	-1.371305	2.273012	0.251086	-1.070016	2.483842	0.191712
C	-1.039834	3.624776	-0.347738	-1.347321	3.504682	-0.266432	-0.893291	3.690452	-0.354272
C	-2.085122	4.556139	0.191908	-2.440964	4.355901	0.308842	-1.874368	4.684009	0.195334
O	-0.180749	3.932550	-1.148554	-0.539522	3.875507	-1.093390	-0.045111	3.939169	-1.186499
C	1.810418	0.104785	-0.337852	1.755996	0.208272	-0.360278	1.781172	0.037708	-0.366238
H	1.656128	0.109555	-1.424970	1.565463	0.203504	-1.441536	1.591189	0.032534	-1.447569
C	3.353219	0.166074	-0.098792	3.297235	0.384280	-0.173168	3.332346	0.028969	-0.178583
C	3.785590	0.677901	1.289018	3.737235	0.924223	1.201536	3.833835	0.541371	1.185656
C	3.964800	1.096260	-1.157968	3.801085	1.359799	-1.248151	3.951398	0.912698	-1.272681
C	3.930546	-1.245133	-0.316408	3.970539	-0.979304	-0.416908	3.835171	-1.411224	-0.391252
C	3.276431	-2.288341	0.580124	3.427261	-2.070728	0.496008	3.162160	-2.409409	0.541815
C	1.798231	-2.435605	0.235197	1.953048	-2.326877	0.201460	1.667818	-2.489786	0.248360
H	1.315459	-3.125724	0.952288	1.548307	-3.052452	0.931655	1.177425	-3.146433	0.991218
O	1.677751	-2.984187	-1.084515	1.827952	-2.880407	-1.115664	1.476003	-3.048744	-1.058464
C	1.038329	-1.098142	0.257775	1.096466	-1.050047	0.255767	0.973962	-1.117235	0.275983
C	0.787333	-0.599207	1.700428	0.858929	-0.573865	1.708175	0.798648	-0.584217	1.717336
O	0.810431	0.817647	1.658093	0.774045	0.840838	1.671337	0.883972	0.829424	1.648493
C	-0.350286	-1.216162	-0.494321	-0.304819	-1.271044	-0.449078	-0.445007	-1.178695	-0.424381
O	-0.165656	-1.252511	-1.904168	-0.167126	-1.290762	-1.863991	-0.313218	-1.241438	-1.839113
C	-1.157790	0.047634	-0.232650	-1.194513	-0.071266	-0.153671	-1.180900	0.125658	-0.152779
C	-2.451837	0.034992	0.092576	-2.471308	-0.179159	0.217490	-2.458569	0.178925	0.226632
C	-3.325396	-1.200804	0.182529	-3.237099	-1.471625	0.351577	-3.374590	-1.016146	0.387739
C	-4.575361	-0.916850	-0.629741	-4.597090	-1.374080	-0.312794	-4.718492	-0.650096	-0.215328
C	-5.836307	-0.902119	-0.199364	-5.007809	-0.436719	-1.165585	-5.398040	-1.317876	-1.146014
C	-3.649102	-1.481316	1.656326	-3.457951	-1.768196	1.849158	-3.608674	-1.266041	1.894418
C	-2.588543	-2.386939	-0.467443	-2.448906	-2.604662	-0.335436	-2.737875	-2.233078	-0.290806
C	-1.131841	-2.459092	-0.034255	-0.973372	-2.570418	0.034347	-1.268513	-2.377165	0.079365
H	1.247616	3.036566	-0.339706	0.978334	3.088937	-0.332661	1.361598	2.990997	-0.404511
H	-2.078892	4.517748	1.289696	-2.386524	4.329436	1.405720	-1.844700	4.661088	1.293126
H	-3.078157	4.226892	-0.146670	-3.418016	3.946026	0.014843	-2.891006	4.404150	-0.116714
H	-1.888250	5.574246	-0.158805	-2.339488	5.382883	-0.056250	-1.632126	5.684983	-0.175731
H	3.456450	1.709785	1.449665	3.337441	1.928197	1.377865	3.551711	1.587792	1.342204
H	4.885705	0.646172	1.352537	4.838161	0.974881	1.227729	4.933306	0.465553	1.211601
H	3.391214	0.076771	2.118954	3.417269	0.293752	2.041672	3.444896	-0.032140	2.037525
H	3.579925	2.118303	-1.048782	3.345296	2.350056	-1.120814	3.621011	1.953961	-1.166710
H	3.733193	0.742703	-2.175693	3.561355	0.992351	-2.259047	3.667463	0.556596	-2.276164
H	5.061427	1.128483	-1.054323	4.895202	1.473343	-1.181973	5.051233	0.892117	-1.207017
H	5.018390	-1.219449	-0.143376	5.058729	-0.872742	-0.280812	4.928055	-1.434798	-0.252982
H	3.779723	-1.543247	-1.367329	3.806560	-1.285463	-1.463549	3.636808	-1.716964	-1.432039
H	3.390850	-2.026278	1.642534	3.558924	-1.804257	1.555291	3.324580	-2.138934	1.595843
H	3.763156	-3.272013	0.460185	3.981301	-3.015058	0.353861	3.597180	-3.416872	0.420753
H	2.193637	-3.795985	-1.125381	2.400419	-3.651938	-1.177626	1.949620	-3.885638	-1.104499
H	1.566951	-0.948843	2.397427	1.686023	-0.865277	2.376574	1.586280	-0.957854	2.392484
H	-0.185622	-0.935639	2.091999	-0.071880	-0.982924	2.131626	-0.173645	-0.869008	2.149278
H	0.474109	-1.965376	-2.058196	0.518133	-1.953516	-2.043657	0.285902	-1.986003	-2.005610
H	-2.949148	0.984494	0.302822	-3.020970	0.733960	0.456251	-2.897484	1.157486	0.436289
H	-4.377324	-0.701962	-1.687197	-5.278549	-2.197962	-0.065257	-5.164759	0.261412	0.203697
H	-6.105148	-1.104378	0.839907	-4.365001	0.395356	-1.464072	-5.019840	-2.233367	-1.605961
H	-6.657295	-0.685413	-0.886267	-6.004958	-0.479443	-1.608699	-6.372952	-0.962510	-1.486231
H	-2.731220	-1.693553	2.223591	-2.502275	-1.894150	2.379334	-2.666680	-1.499428	2.412916
H	-4.321649	-2.347695	1.755501	-4.049444	-2.688654	1.981354	-4.310230	-2.102106	2.040572
H	-4.132288	-0.612855	2.129415	-4.001014	-0.941777	2.332696	-4.035960	-0.373450	2.377536
H	-2.609027	-2.267068	-1.562264	-2.534660	-2.480242	-1.425606	-2.796120	-2.107319	-1.382943
H	-3.115148	-3.324180	-0.229542	-2.890794	-3.580149	-0.076391	-3.292931	-3.147467	-0.028640
H	-0.654383	-3.344162	-0.475270	-0.450614	-3.415533	-0.433100	-0.855822	-3.287219	-0.375627
H	-1.066593	-2.569654	1.059565	-0.853560	-2.680906	1.123576	-1.159724	-2.486111	1.169834

	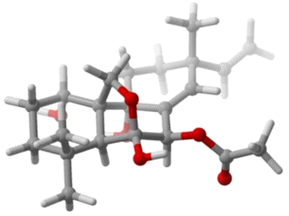 conformer **3-IV**			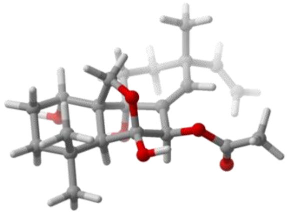 conformer **3-V**			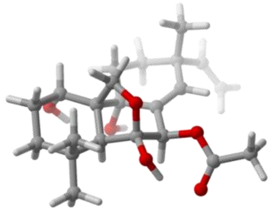 conformer **3-VI**		
O	1.47155	2.63433	0.42722	1.28298	2.72360	0.34416	1.42934	2.55059	0.37606
C	0.96887	1.34705	0.38277	0.87454	1.40273	0.35330	0.89845	1.27044	0.33158
C	-0.40968	1.35083	-0.31051	-0.52840	1.28709	-0.27714	-0.50797	1.28079	-0.32603
H	-0.27071	1.58360	-1.37549	-0.45663	1.49767	-1.35394	-0.38179	1.50291	-1.39791
O	-1.17854	2.39938	0.28774	-1.33632	2.30136	0.33162	-1.34412	2.29106	0.25069
C	-1.92833	3.17130	-0.54221	-2.30742	2.85898	-0.43324	-1.29718	3.52398	-0.26923
C	-2.72736	4.16491	0.25520	-3.08685	3.86850	0.36334	-2.33734	4.41111	0.34857
O	-1.94974	3.04940	-1.73730	-2.51525	2.55526	-1.57850	-0.50815	3.86513	-1.12515
C	1.88119	0.33573	-0.32173	1.82876	0.44034	-0.36495	1.75971	0.19352	-0.36322
H	1.71811	0.37772	-1.40608	1.62033	0.44221	-1.44226	1.57089	0.20010	-1.44453
C	3.41362	0.52073	-0.08453	3.35204	0.74098	-0.19577	3.30466	0.34566	-0.18577
C	3.80896	0.99192	1.32816	3.76762	1.27677	1.18771	3.76092	0.87940	1.18583
C	3.92425	1.56248	-1.09280	3.74616	1.78877	-1.24908	3.81230	1.31832	-1.26183
C	4.11013	-0.81964	-0.38357	4.13202	-0.55366	-0.49110	3.95075	-1.02923	-0.43837
C	3.55711	-1.96623	0.45330	3.69767	-1.71403	0.39530	3.41160	-2.11455	0.48245
C	2.09251	-2.22191	0.11125	2.24326	-2.08324	0.11984	1.93343	-2.36531	0.19288
H	1.68165	-2.99148	0.79109	1.91585	-2.86151	0.83428	1.53463	-3.05415	0.96275
O	2.00194	-2.70119	-1.23691	2.13556	-2.60406	-1.21147	1.85350	-3.03108	-1.05730
C	1.21787	-0.96126	0.21849	1.28396	-0.88642	0.23256	1.09616	-1.06543	0.25506
C	0.94515	-0.56634	1.68793	1.04221	-0.47268	1.70248	0.87296	-0.58460	1.70749
O	0.83023	0.84664	1.71267	0.82681	0.92905	1.69921	0.78340	0.83127	1.67164
C	-0.17278	-1.16432	-0.52000	-0.11579	-1.20856	-0.44479	-0.31085	-1.26456	-0.41463
O	-0.01295	-1.10962	-1.93034	-0.01360	-1.17750	-1.86067	-0.06762	-1.32928	-1.83855
C	-1.07855	0.01023	-0.17414	-1.08917	-0.09505	-0.08377	-1.19301	-0.05371	-0.15294
C	-2.34536	-0.12553	0.21737	-2.32523	-0.31397	0.36083	-2.47946	-0.15150	0.19241
C	-3.11021	-1.43311	0.27560	-2.99455	-1.66083	0.46004	-3.25187	-1.43739	0.35253
C	-4.41740	-1.20863	-0.46184	-4.37184	-1.61275	-0.17673	-4.60878	-1.35312	-0.31950
C	-5.65251	-1.33567	0.02174	-4.83062	-0.67356	-1.00366	-5.02397	-0.42190	-1.17709
C	-3.33653	-1.82701	1.74132	-3.15784	-2.04275	1.94423	-3.47517	-1.69458	1.85740
C	-2.31021	-2.51130	-0.48145	-2.14691	-2.70256	-0.29958	-2.46907	-2.59681	-0.29734
C	-0.83354	-2.49305	-0.11236	-0.66403	-2.57007	0.01967	-0.98879	-2.55875	0.05783
H	0.75657	3.18322	0.77745	0.53631	3.23118	0.69086	1.04873	3.06765	-0.35181
H	-2.10379	4.64005	1.02396	-2.41217	4.52891	0.92396	-2.19463	4.43056	1.43796
H	-3.54097	3.63364	0.77153	-3.71288	3.33643	1.09527	-3.33916	4.00346	0.15229
H	-3.15454	4.91574	-0.41779	-3.72741	4.44882	-0.30890	-2.25193	5.42103	-0.06482
H	3.37687	1.97310	1.55180	3.27364	2.22936	1.40688	3.38768	1.89395	1.36188
H	4.90699	1.07062	1.38306	4.85816	1.43676	1.19461	4.86269	0.90263	1.20705
H	3.48849	0.30602	2.12349	3.53118	0.58981	2.01101	3.43155	0.25746	2.02817
H	3.42772	2.52963	-0.94028	3.18890	2.72256	-1.09878	3.36985	2.31506	-1.13269
H	3.73266	1.23500	-2.12732	3.53688	1.42113	-2.26656	3.56680	0.95363	-2.27245
H	5.01105	1.70578	-0.98069	4.82367	2.01075	-1.18624	4.90765	1.41950	-1.19994
H	5.19304	-0.70864	-0.21346	5.20986	-0.36047	-0.36900	5.04331	-0.93518	-0.32878
H	3.97672	-1.06934	-1.44927	3.97469	-0.84109	-1.54395	3.76063	-1.34082	-1.47929
H	3.66130	-1.75950	1.52895	3.82900	-1.47136	1.46040	3.56480	-1.85149	1.54046
H	4.12527	-2.89502	0.27101	4.32446	-2.60410	0.21191	3.93864	-3.06443	0.31079
H	2.57266	-3.47138	-1.32593	2.75436	-3.33577	-1.30374	1.28300	-2.51677	-1.64767
H	1.76688	-0.87259	2.35642	1.91026	-0.70152	2.34279	1.71002	-0.87703	2.36033
H	0.01632	-1.01561	2.07280	0.16436	-0.97633	2.13637	-0.05098	-0.99258	2.14723
H	0.68129	-1.75567	-2.13379	0.71524	-1.77954	-2.07746	-0.91926	-1.45253	-2.27460
H	-2.90946	0.76651	0.50358	-2.93595	0.54027	0.66480	-3.02689	0.76932	0.40628
H	-4.28894	-0.90396	-1.50773	-5.01302	-2.47043	0.06350	-5.28404	-2.18187	-0.07216
H	-5.85247	-1.63137	1.05420	-4.22731	0.19084	-1.29542	-4.39175	0.41991	-1.47136
H	-6.52123	-1.14648	-0.61253	-5.83194	-0.75019	-1.43316	-6.01916	-0.47438	-1.62324
H	-2.37778	-1.99191	2.25421	-2.18253	-2.13591	2.44524	-2.52025	-1.81315	2.39014
H	-3.92821	-2.75284	1.81586	-3.68881	-3.00328	2.04381	-4.07338	-2.60674	2.01209
H	-3.86779	-1.03317	2.28835	-3.73761	-1.27669	2.48196	-4.01223	-0.85183	2.31807
H	-2.39031	-2.32363	-1.56397	-2.28166	-2.54245	-1.37993	-2.59526	-2.53202	-1.39246
H	-2.75006	-3.50160	-0.28598	-2.50434	-3.71834	-0.06677	-2.90676	-3.56228	0.00074
H	-0.30744	-3.30760	-0.62827	-0.09771	-3.35916	-0.49331	-0.45782	-3.40517	-0.39683
H	-0.71293	-2.66726	0.96838	-0.49628	-2.70439	1.10002	-0.87124	-2.65698	1.14682

Table S12 Coordinates of Compound **4**

	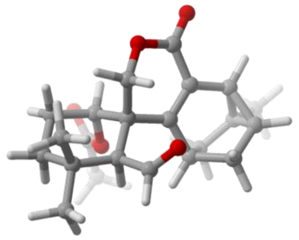 conformer **4-I**			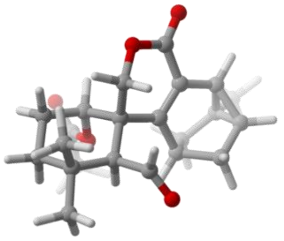 conformer **4-II**		
C	-3.900444	-1.544238	0.369116	-3.910974	-1.465707	0.645211
C	-3.248782	-0.466278	-0.514101	-3.243980	-0.525432	-0.374977
C	-4.012805	-0.438831	-1.852013	-3.984432	-0.695369	-1.712059
C	-3.402495	0.928608	0.125766	-3.389648	0.940418	0.078310
C	-2.470828	1.190168	1.303809	-2.464043	1.328916	1.227934
C	-1.012235	0.982724	0.915770	-1.000955	1.075483	0.880201
H	-0.356640	1.193230	1.770392	-0.353437	1.363155	1.719139
O	-0.669103	1.888761	-0.155240	-0.641366	1.870613	-0.267365
C	-0.335235	3.150717	0.191908	-0.290826	3.155208	-0.038120
C	-0.039820	3.976132	-1.030767	0.014255	3.858906	-1.331861
O	-0.298594	3.542032	1.330212	-0.248612	3.648690	1.059684
C	-0.730307	-0.439452	0.376195	-0.721274	-0.393644	0.484942
C	-0.834310	-1.455527	1.526820	-0.820842	-1.289470	1.730499
O	0.117686	-1.214216	2.563137	0.163608	-0.996386	2.715794
C	1.423161	-1.130944	2.208108	1.463015	-0.991780	2.323008
C	1.694413	-0.867513	0.778672	1.703857	-0.854898	0.870366
C	0.723577	-0.507055	-0.084682	0.722229	-0.524898	0.004057
C	2.340237	0.938195	-1.278531	2.342459	0.811299	-1.300039
C	4.858620	0.832773	-0.593361	4.871379	0.694810	-0.652192
C	3.091708	-0.889042	0.300707	3.089648	-0.937356	0.369152
H	3.839922	-1.191252	1.032556	3.846252	-1.210451	1.103745
O	2.275756	-1.215540	3.054922	2.332394	-1.038252	3.154202
C	-1.722668	-0.724889	-0.787665	-1.725054	-0.801285	-0.639441
H	-1.477206	0.018827	-1.567010	-1.473754	-0.163034	-1.497655
C	-1.510649	-2.051899	-1.483791	-1.488221	-2.214778	-1.130508
O	-0.772089	-2.938030	-1.125443	-1.129710	-2.478115	-2.252999
H	-3.617803	-1.465977	1.426403	-3.587607	-1.291301	1.679214
H	-3.645445	-2.561096	0.030836	-3.738449	-2.527371	0.413179
H	-4.995999	-1.444909	0.323733	-5.000047	-1.305122	0.623075
H	-3.515297	0.209094	-2.591203	-3.529858	-0.079307	-2.503076
H	-5.025414	-0.038928	-1.687014	-5.036426	-0.388886	-1.603835
H	-4.131789	-1.438707	-2.294687	-3.974119	-1.740882	-2.057043
H	-4.451527	1.061655	0.436277	-4.438958	1.127663	0.358202
H	-3.206597	1.687693	-0.649471	-3.172163	1.593899	-0.782347
H	-2.706392	0.534498	2.155769	-2.712115	0.775359	2.146613
H	-2.592120	2.218714	1.671925	-2.580363	2.392867	1.478444
H	-0.963072	4.101713	-1.615448	0.411144	4.857186	-1.120023
H	0.691021	3.463708	-1.671132	-0.909130	3.945530	-1.923305
H	0.339149	4.957220	-0.726033	0.732802	3.276139	-1.924047
H	-0.691285	-2.466348	1.122941	-0.725591	-2.348014	1.439118
H	-1.797385	-1.411602	2.040679	-1.769501	-1.169077	2.258714
H	2.019051	1.755693	-0.616243	2.051715	1.673144	-0.681089
H	2.606931	1.365189	-2.259526	2.598405	1.174664	-2.308952
H	4.806726	1.686132	0.101089	4.847536	1.586956	-0.006782
H	5.209251	1.207953	-1.568531	5.211272	1.007202	-1.652826
H	5.616125	0.134222	-0.208242	5.622382	0.004187	-0.240924
H	-2.097054	-2.173127	-2.426270	-1.675076	-3.040458	-0.400452
C	3.518029	0.161405	-0.723009	3.516161	0.044340	-0.722383
C	3.331628	-1.267922	-1.159353	3.294730	-1.402603	-1.072191
H	4.180413	-1.913673	-1.387707	4.127387	-2.077113	-1.275680
C	2.068103	-1.347197	-1.987989	2.015726	-1.501834	-1.872718
H	1.526773	-2.297343	-1.881958	1.459474	-2.436553	-1.719674
H	2.306253	-1.192119	-3.052417	2.233333	-1.409236	-2.948096
C	1.244863	-0.143139	-1.452660	1.222956	-0.255218	-1.393803
H	0.457610	0.174703	-2.144093	0.427398	0.025804	-2.088486

Table S13 Coordinates of Compound **4a**

	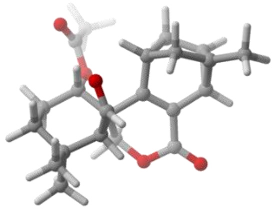 conformer **4a-I**			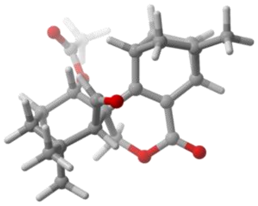 conformer **4a-II**		
C	3.492657	-2.307392	-1.494308	3.508871	-2.272325	-1.434789
C	2.984699	-1.338382	-0.410082	3.018827	-1.268286	-0.376701
C	3.538439	-1.851396	0.932726	3.603504	-1.723160	0.973639
C	3.515726	0.075571	-0.710606	3.541596	0.136552	-0.735582
C	2.902391	1.158314	0.176748	2.916402	1.253434	0.097724
C	1.388744	1.168444	0.016815	1.404038	1.229251	-0.067248
H	1.165511	1.404120	-1.029596	1.188176	1.397792	-1.131154
O	0.798075	2.184534	0.848890	0.795244	2.302221	0.670352
C	0.629433	3.402705	0.289555	0.606330	3.463682	0.004606
C	-0.030703	4.348782	1.254218	-0.047676	4.489637	0.886513
O	0.954001	3.671060	-0.839761	0.911768	3.626824	-1.150507
C	0.723903	-0.167347	0.379408	0.753912	-0.086105	0.387307
C	0.794081	-0.422879	1.894242	0.844665	-0.237855	1.914194
O	0.172027	-1.654614	2.260708	0.215485	-1.430534	2.375917
C	-1.112340	-1.840909	1.861537	-1.080231	-1.624524	2.017694
C	-1.609922	-0.911843	0.822173	-1.579800	-0.782180	0.907364
C	-0.776162	-0.142146	0.091837	-0.746813	-0.086276	0.107763
C	-2.383617	-0.178091	-1.802359	-2.369733	-0.291905	-1.775038
C	-4.723229	-1.276702	-1.422876	-4.707713	-1.339760	-1.275255
C	-3.059169	-0.832477	0.552419	-3.029098	-0.720438	0.638006
H	-3.695097	-1.477168	1.158403	-3.664402	-1.302765	1.304584
O	-1.756838	-2.741689	2.335192	-1.732988	-2.464847	2.580606
C	1.412078	-1.323210	-0.422909	1.449361	-1.283649	-0.352884
H	1.073524	-2.271715	0.022414	1.120950	-2.212203	0.136515
C	0.937101	-1.343306	-1.868548	0.907741	-1.379158	-1.766418
O	0.860732	-0.373837	-2.586626	0.337325	-2.348099	-2.202249
H	3.203175	-1.996135	-2.509256	3.194908	-1.998339	-2.453414
H	3.118585	-3.330020	-1.324396	3.125261	-3.283625	-1.229707
H	4.592313	-2.347660	-1.471014	4.608762	-2.315143	-1.431323
H	3.524173	-1.094639	1.726695	3.575614	-0.943742	1.745643
H	4.590467	-2.149245	0.804480	4.661775	-1.997015	0.842621
H	2.979149	-2.727535	1.293895	3.070864	-2.602467	1.365280
H	3.300682	0.323256	-1.763061	3.340194	0.340562	-1.801847
H	4.612672	0.076015	-0.604109	4.637895	0.151050	-0.626087
H	3.284750	2.144260	-0.124459	3.286275	2.229576	-0.247986
H	3.176844	1.018463	1.234492	3.188605	1.165430	1.161034
H	-1.112315	4.145748	1.265162	-1.128808	4.287230	0.922512
H	0.131789	5.379842	0.921972	0.110297	5.487340	0.462938
H	0.351000	4.199424	2.272073	0.341454	4.430659	1.910619
H	0.297315	0.410240	2.413163	0.368211	0.636889	2.382265
H	1.814398	-0.504141	2.273470	1.871328	-0.307230	2.279049
H	-1.847648	-1.048844	-2.207583	-1.847978	-1.199854	-2.106615
H	-2.772585	0.404447	-2.652648	-2.768406	0.223116	-2.664099
H	-4.438281	-2.260917	-1.826804	-4.425396	-2.356620	-1.589220
H	-5.194554	-0.700084	-2.234906	-5.184351	-0.838689	-2.133055
H	-5.480010	-1.441647	-0.641584	-5.458327	-1.430454	-0.476089
H	0.668973	-2.352227	-2.267204	1.061720	-0.479680	-2.416790
C	-3.523418	-0.551507	-0.874854	-3.502290	-0.570444	-0.805690
C	-3.610136	0.540505	0.158354	-3.575975	0.613507	0.121291
H	-4.563180	0.852826	0.587253	-4.522931	0.971920	0.526808
C	-2.535737	1.561300	-0.149034	-2.499124	1.595043	-0.287667
H	-2.104163	2.030774	0.747983	-2.057862	2.142826	0.558560
H	-2.937069	2.351800	-0.802246	-2.900308	2.322545	-1.010420
C	-1.482247	0.722022	-0.923341	-1.455384	0.677229	-0.985462
H	-0.799809	1.336783	-1.516515	-0.776647	1.242760	-1.633780

Table S14 Coordinates of Compound **4b**

	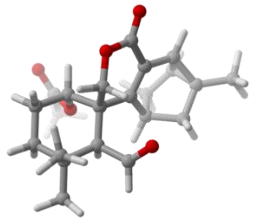 conformer **4b-I**			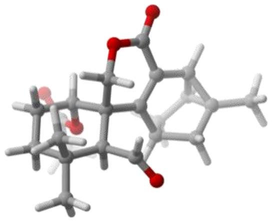 conformer **4b-II**			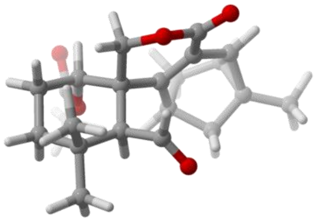 conformer **4b-III**		
C	-3.476208	-2.224127	0.460861	-3.522085	-2.080558	0.811734	3.432203	-2.280159	-0.079672
C	-3.040429	-1.122850	-0.520981	-3.036566	-1.160946	-0.323394	2.902432	-1.091688	0.743943
C	-3.717902	-1.413881	-1.874099	-3.659614	-1.675148	-1.631965	3.310025	-1.328463	2.207431
C	-3.541133	0.257654	-0.049731	-3.526132	0.280877	-0.085148	3.560795	0.215787	0.262978
C	-2.757941	0.850827	1.116015	-2.762562	1.021860	1.009030	3.004377	0.738983	-1.058278
C	-1.271610	0.948202	0.794963	-1.265909	1.063489	0.718570	1.507314	1.009130	-0.946201
H	-0.727708	1.393967	1.637649	-0.736670	1.602672	1.515583	1.111744	1.411817	-1.887475
O	-1.086420	1.790587	-0.363379	-1.042977	1.755487	-0.526388	1.338919	2.026900	0.055274
C	-1.081585	3.125474	-0.156621	-1.010425	3.105531	-0.479820	1.005500	3.267233	-0.371645
C	-0.915656	3.862741	-1.457519	-0.818989	3.678272	-1.857236	0.812820	4.191933	0.796872
O	-1.208141	3.632832	0.928165	-1.135181	3.740224	0.536077	0.865232	3.565141	-1.530534
C	-0.645396	-0.418440	0.430520	-0.647448	-0.342841	0.539727	0.713421	-0.269359	-0.563222
C	-0.579001	-1.304519	1.686548	-0.599735	-1.061502	1.898142	0.739203	-1.123624	-1.850977
O	0.241392	-0.746809	2.713214	0.250725	-0.423741	2.844378	-0.182112	-2.202584	-1.889477
C	1.512391	-0.408433	2.385477	1.532656	-0.178664	2.470875	-1.488774	-1.891780	-1.680804
C	1.790317	-0.239034	0.943163	1.800439	-0.187996	1.016523	-1.740981	-0.673245	-0.881793
C	0.808598	-0.198957	0.019923	0.809005	-0.208215	0.100103	-0.751742	0.017640	-0.273401
C	2.409525	-0.896715	-1.732389	2.377388	-1.105070	-1.593849	-2.212767	0.255301	1.723180
C	4.937142	-1.086689	-1.102029	4.906148	-1.299722	-0.966748	-4.714452	-0.478139	1.537071
C	3.179642	0.008670	0.507273	3.190730	-0.021053	0.549338	-3.139084	-0.332517	-0.553615
H	3.936914	-0.028012	1.289594	3.954824	-0.003084	1.325590	-3.903162	-0.890846	-1.093028
O	2.318648	-0.211529	3.258552	2.353469	0.084121	3.311126	-2.345086	-2.650606	-2.052978
C	-1.483144	-1.047491	-0.720220	-1.484459	-1.117236	-0.527310	1.344161	-0.919457	0.711620
H	-1.371773	-0.353226	-1.572442	-1.343997	-0.561073	-1.464334	1.105093	-0.233500	1.539826
C	-0.937835	-2.354679	-1.253557	-0.915244	-2.492845	-0.808525	0.640230	-2.218037	1.063892
O	-0.036547	-3.002278	-0.776582	-0.441607	-2.813111	-1.872027	0.069284	-2.398775	2.113243
H	-3.273129	-1.974319	1.509776	-3.291870	-1.698904	1.814445	3.306974	-2.157491	-1.163530
H	-2.981102	-3.184411	0.245845	-3.107884	-3.097036	0.735254	2.958370	-3.230861	0.205467
H	-4.562074	-2.384905	0.375793	-4.617524	-2.175141	0.753246	4.512299	-2.390389	0.104313
H	-3.342019	-0.749303	-2.668313	-3.320351	-1.084318	-2.496685	2.961787	-0.509410	2.855309
H	-4.802255	-1.244756	-1.784171	-4.757357	-1.606039	-1.581828	4.406540	-1.386289	2.292057
H	-3.582435	-2.454825	-2.202678	-3.399054	-2.727454	-1.824800	2.890433	-2.266519	2.601220
H	-4.608080	0.175969	0.213992	-4.602120	0.260873	0.152100	4.648634	0.060310	0.181490
H	-3.481483	0.955550	-0.901230	-3.422042	0.843892	-1.027103	3.408458	0.991037	1.030704
H	-2.883336	0.251689	2.030592	-2.921036	0.556329	1.993942	3.192936	0.032442	-1.881365
H	-3.129440	1.855982	1.360157	-3.124644	2.055300	1.104433	3.502648	1.678211	-1.341322
H	-0.795574	4.932671	-1.257943	0.021236	3.186074	-2.365133	1.607398	4.050365	1.540764
H	-1.806635	3.700972	-2.082067	-0.646268	4.757054	-1.783418	-0.146700	3.952150	1.280410
H	-0.048821	3.474978	-2.009729	-1.723636	3.490208	-2.454322	0.787266	5.228360	0.444218
H	-0.194838	-2.293597	1.404630	-0.259063	-2.100492	1.760052	1.712496	-1.588754	-2.022804
H	-1.552776	-1.425367	2.166752	-1.573621	-1.088879	2.392367	0.529131	-0.457690	-2.705680
H	2.666960	-0.804901	-2.800460	2.621974	-1.115403	-2.668140	-2.446865	0.909126	2.579440
H	2.098822	-1.932143	-1.533255	2.046411	-2.115186	-1.314219	-1.775159	-0.676965	2.109554
H	4.901470	-2.165303	-0.882476	4.848895	-2.352296	-0.647677	-4.548719	-1.485091	1.950505
H	5.697932	-0.635775	-0.447624	5.684179	-0.807778	-0.364238	-5.539611	-0.543419	0.812376
H	5.271487	-0.963379	-2.144873	5.231126	-1.282322	-2.019429	-5.037541	0.178703	2.360715
H	-1.431030	-2.707314	-2.191165	-0.959641	-3.236857	0.024596	0.676666	-3.024770	0.295561
C	3.590052	-0.450050	-0.888990	3.575960	-0.614268	-0.804022	-3.461563	0.047495	0.890271
C	3.393695	1.018795	-0.620365	3.416843	0.876205	-0.669230	-3.449174	1.114569	-0.174112
H	4.233934	1.709104	-0.536928	4.273832	1.550474	-0.652221	-4.371203	1.554355	-0.556331
C	2.111222	1.452276	-1.291952	2.138061	1.277635	-1.367377	-2.223303	1.977709	0.037676
H	2.319539	1.821369	-2.309061	2.345489	1.545407	-2.415761	-2.472819	2.820491	0.702630
H	1.573175	2.234264	-0.737196	1.626135	2.122082	-0.883793	-1.794551	2.384952	-0.891011
C	1.305075	0.127432	-1.366550	1.299137	-0.027925	-1.315931	-1.258146	0.998926	0.759113
H	0.503905	0.175353	-2.111376	0.494055	-0.041218	-2.054778	-0.447392	1.499643	1.289807

Table S15 Coordinates of Compound **4c**

	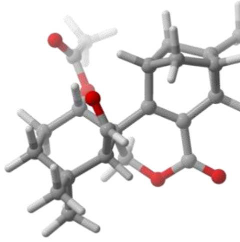 conformer **4c-I**			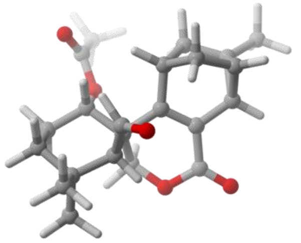 conformer **4c-II**		
C	-4.048775	-1.571769	1.310189	4.089298	-1.429699	-1.259464
C	-3.252361	-0.808907	0.234833	3.290217	-0.640005	-0.207982
C	-3.751251	-1.322772	-1.129071	3.837409	-1.054177	1.170875
C	-3.528953	0.699239	0.379713	3.512226	0.868169	-0.436076
C	-2.628075	1.571360	-0.494474	2.578108	1.755269	0.384400
C	-1.166250	1.313853	-0.157342	1.131840	1.412118	0.059507
H	-1.011777	1.591358	0.891210	0.981493	1.609081	-1.010712
O	-0.306163	2.128487	-0.976171	0.231582	2.266446	0.784666
C	0.043263	3.333707	-0.475802	-0.160261	3.401300	0.163283
C	0.975429	4.052707	-1.411500	-1.109476	4.186144	1.024261
O	-0.335818	3.751535	0.589301	0.200221	3.713346	-0.944351
C	-0.738415	-0.147827	-0.353066	0.756544	-0.044396	0.372366
C	-0.699243	-0.513885	-1.846271	0.738719	-0.285099	1.890513
O	-0.292202	-1.865959	-2.057623	0.350193	-1.615685	2.221797
C	0.883419	-2.252009	-1.498455	-0.833203	-2.056389	1.721804
C	1.440975	-1.345416	-0.469853	-1.402603	-1.263955	0.608352
C	0.700938	-0.374851	0.105882	-0.674257	-0.350131	-0.064133
C	2.728347	0.975316	0.436999	-2.716981	0.923375	-0.559977
C	5.048876	-0.090287	-0.124029	-5.024005	-0.104080	0.111921
C	2.842640	-1.512711	-0.037336	-2.798273	-1.498050	0.190306
H	3.397148	-2.321337	-0.512694	-3.343874	-2.257165	0.749860
O	1.387530	-3.293071	-1.834853	-1.333817	-3.054397	2.171740
C	-1.716699	-1.083675	0.433978	1.769129	-1.002127	-0.350097
H	-1.522960	-2.112990	0.093907	1.616711	-2.013220	0.055018
C	-1.408726	-1.066689	1.924153	1.394318	-1.112417	-1.815633
O	-1.221607	-0.069682	2.581671	1.111486	-2.150941	-2.359217
H	-3.812202	-1.234848	2.330631	3.816453	-1.155281	-2.289714
H	-3.864032	-2.656608	1.250756	3.926608	-2.512981	-1.150292
H	-5.126842	-1.409432	1.159145	5.165332	-1.230301	-1.141189
H	-3.510386	-0.650397	-1.961659	3.562449	-0.360538	1.975616
H	-4.848324	-1.411475	-1.107251	4.937668	-1.076315	1.138353
H	-3.335183	-2.313446	-1.366347	3.485747	-2.056543	1.457024
H	-3.382238	0.991169	1.432550	3.367000	1.101618	-1.505441
H	-4.587717	0.893169	0.143024	4.562397	1.114914	-0.211774
H	-2.841471	2.632832	-0.302567	2.747831	2.811885	0.131441
H	-2.812549	1.397183	-1.566538	2.765316	1.649949	1.464352
H	1.990503	3.648646	-1.278464	-2.114643	3.747655	0.931498
H	0.985565	5.120326	-1.166619	-1.144326	5.223822	0.675198
H	0.682291	3.892124	-2.456605	-0.814372	4.135523	2.079708
H	-0.000100	0.165991	-2.355374	0.039843	0.429646	2.350825
H	-1.671178	-0.437440	-2.336987	1.717785	-0.158442	2.356746
H	3.212159	1.731663	1.075880	-3.207195	1.595768	-1.282345
H	2.500467	1.433656	-0.538050	-2.499294	1.490515	0.358235
H	5.097888	0.310106	-1.149147	-5.082381	0.407454	1.085870
H	5.576963	-1.055351	-0.116343	-5.538925	-1.071127	0.210841
H	5.597369	0.600446	0.536526	-5.579326	0.500686	-0.623086
H	-1.384381	-2.069502	2.416516	1.387879	-0.155076	-2.398171
C	3.621512	-0.247836	0.326626	-3.593174	-0.292541	-0.315397
C	3.186094	-1.190777	1.416696	-3.139569	-1.345024	-1.290954
H	3.896116	-1.843222	1.926574	-3.835856	-2.062743	-1.726158
C	2.060565	-0.535812	2.183661	-2.021435	-0.765268	-2.126449
H	2.462539	0.042531	3.029794	-2.436568	-0.282013	-3.024879
H	1.320673	-1.245259	2.580063	-1.275874	-1.505838	-2.444047
C	1.454585	0.432368	1.133902	-1.431768	0.320109	-1.185675
H	0.850378	1.219662	1.592844	-0.842009	1.070162	-1.724382

Table S16 Summary of binding energies, amino acid residue involved in the hydrogen bond and hydrophobic interactions of **2** observed in molecular docking studies.

compound	Binding energy (kcal/mol)	Residue involved in the interaction	
		Hydrogen bond	Hydrophobic interaction
**2**	–8.0	GLN257, ARG260	MET114, TRP84, TYR341, TYR367
**AR-C118901**	–9.2	GLU371, TRP366, TYR367	HEM901, VAL346, TYR367, PRO344

Datablock 1 - ellipsoid plot

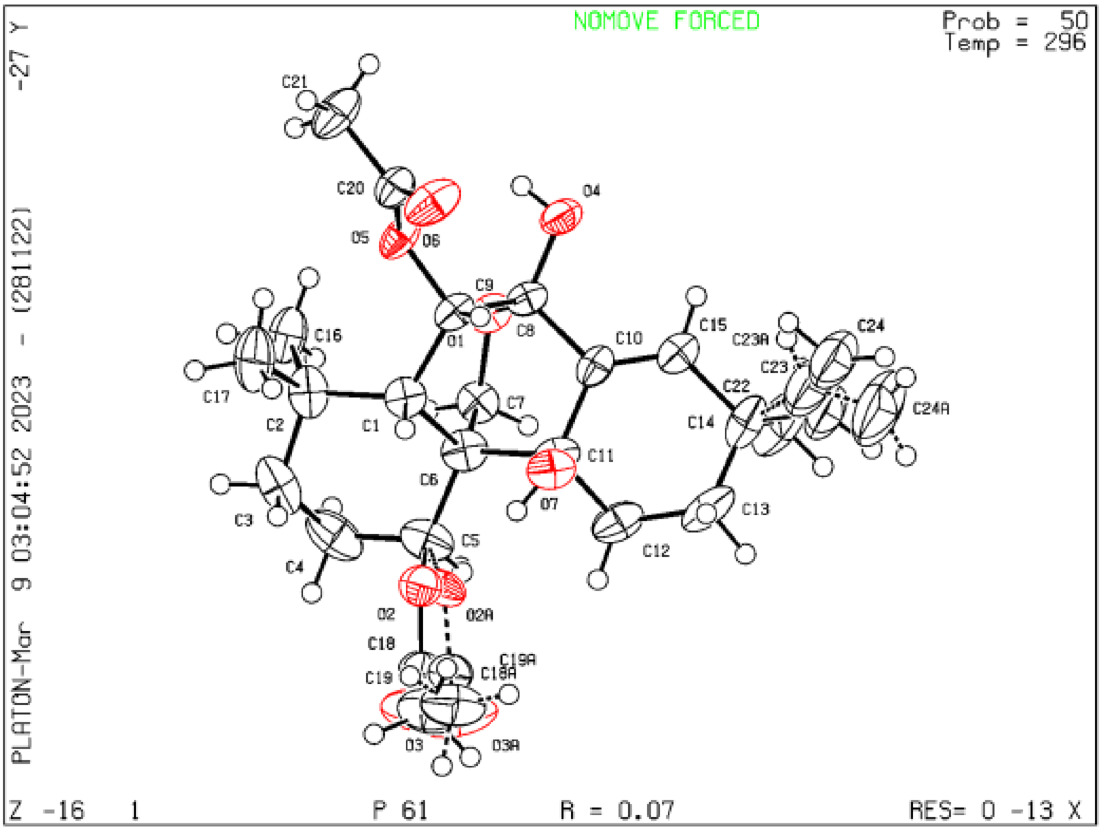

Figure S1 ORTEP drawing of crystal structure of **1**

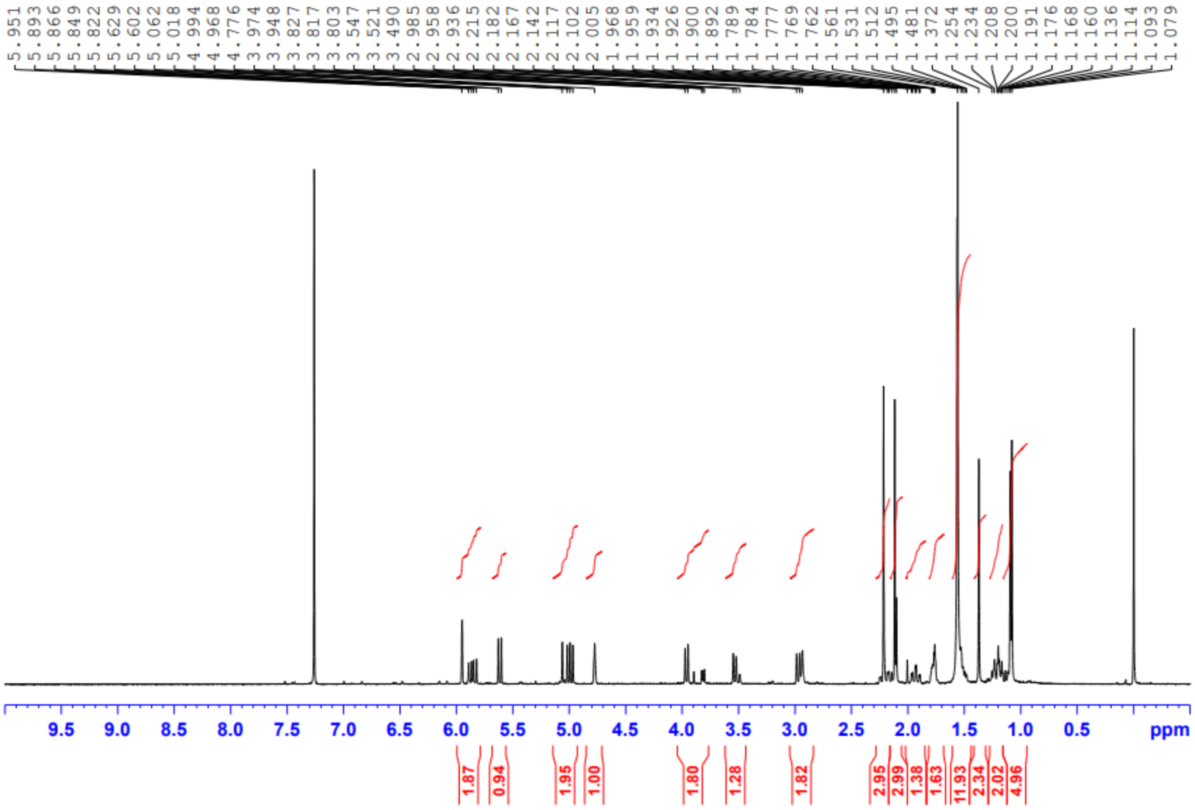

Figure S2 ^{1}H NMR spectrum (400 MHz) of compound **1** in $CDCl_3$

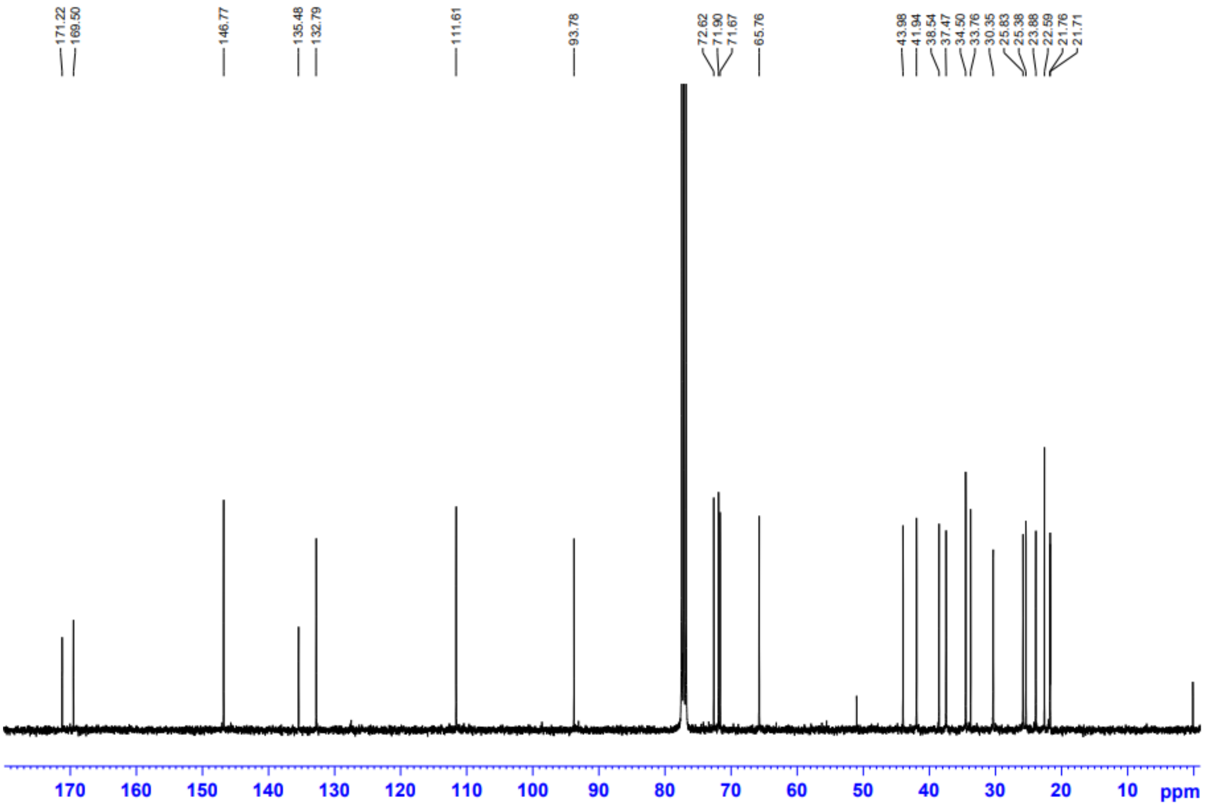

Figure S3 ^{13}C NMR spectrum (100 MHz) of compound **1** in $CDCl_3$

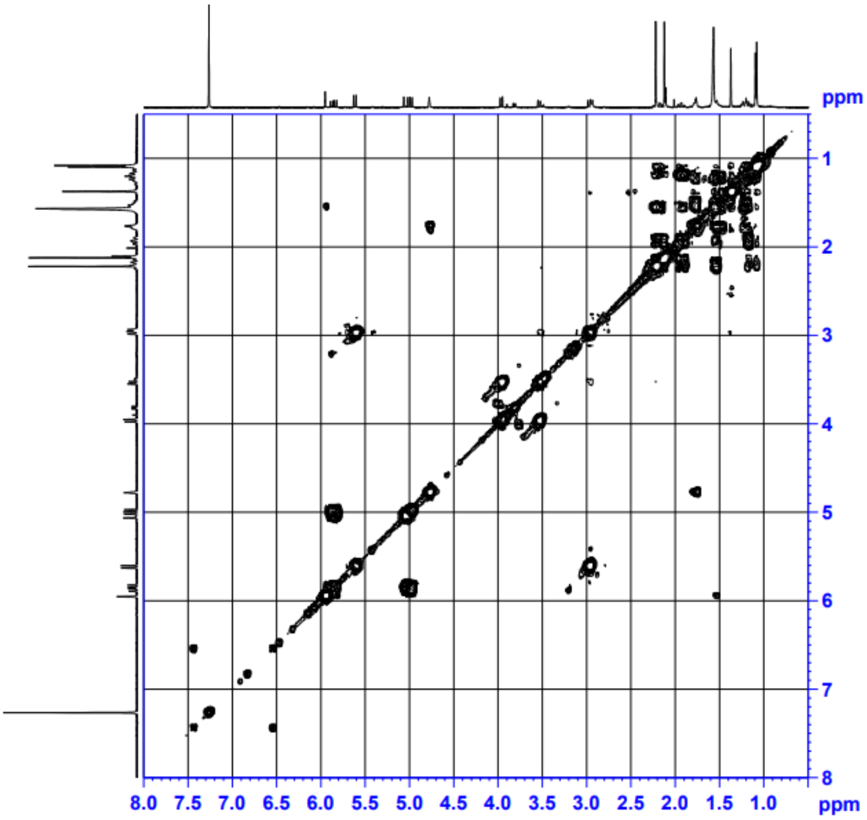

Figure S4 ^1H– ^1H COSY spectrum of compound **1** in $CDCl_3$

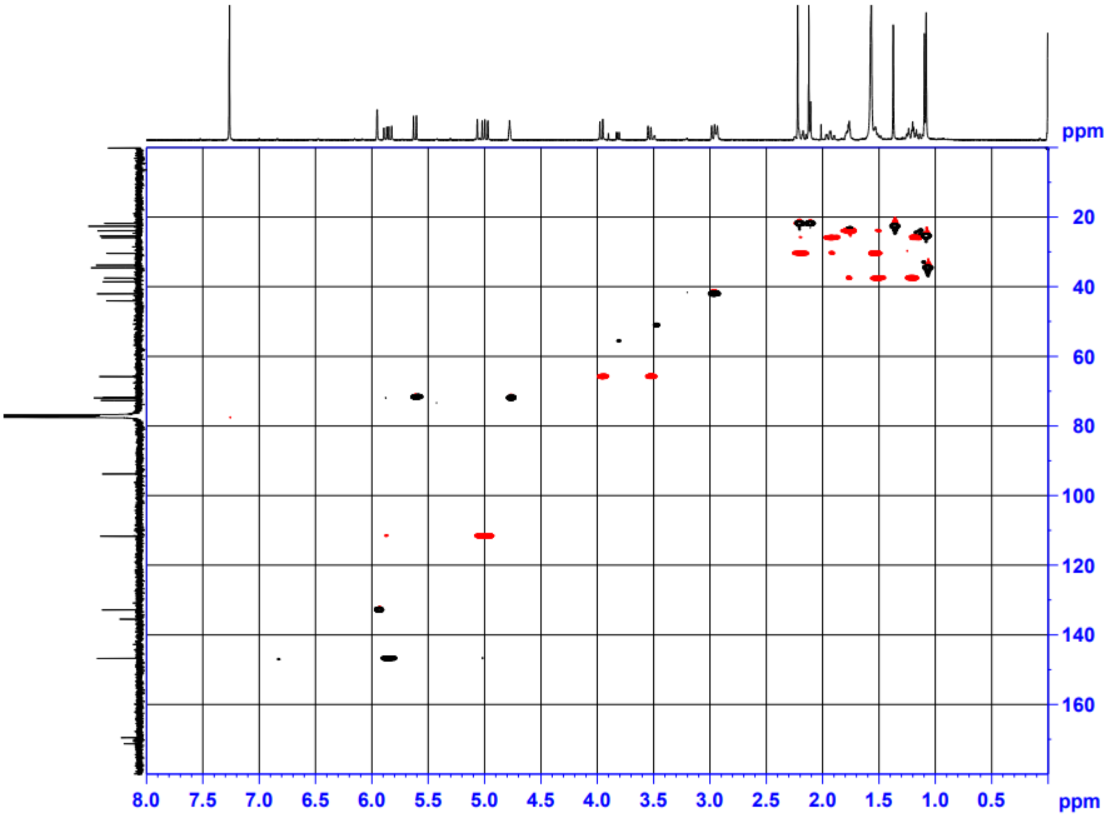

Figure S5 HSQC spectrum of compound **1** in $CDCl_3$

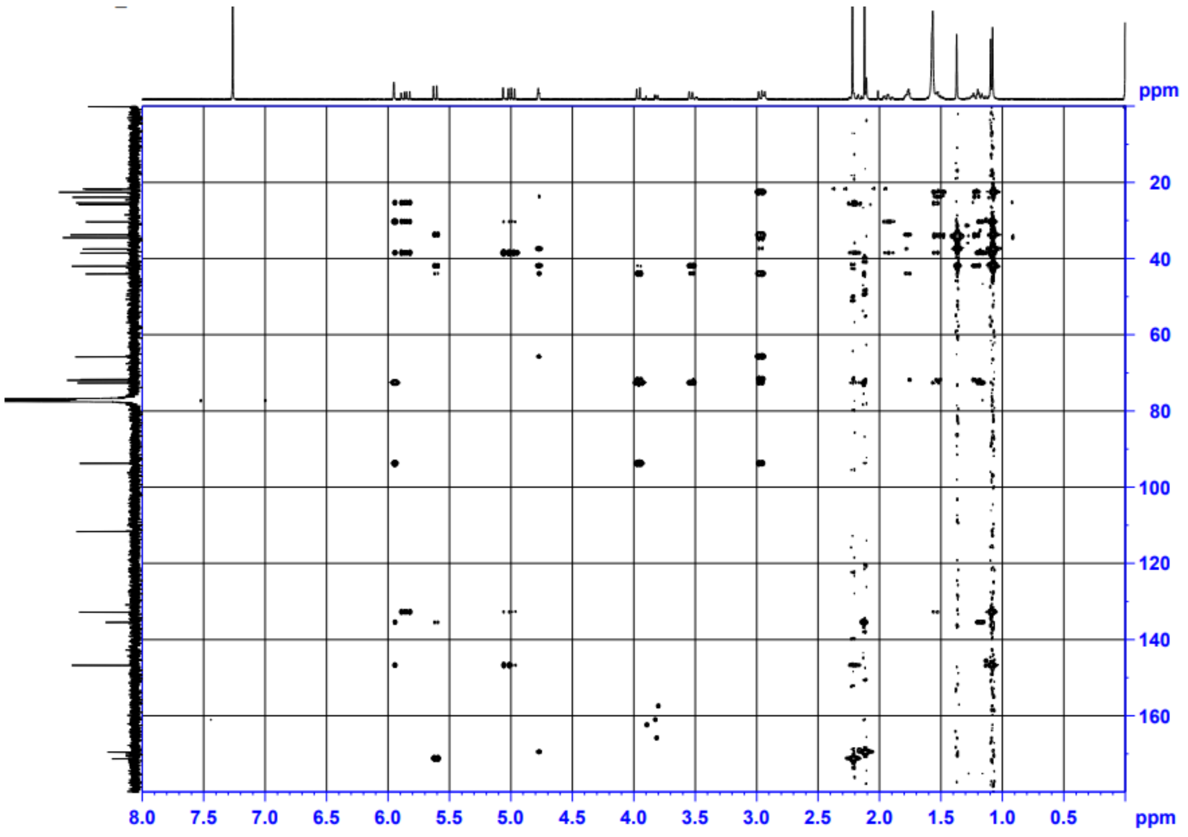

Figure S6 HMBC spectrum of compound **1** in $CDCl_3$

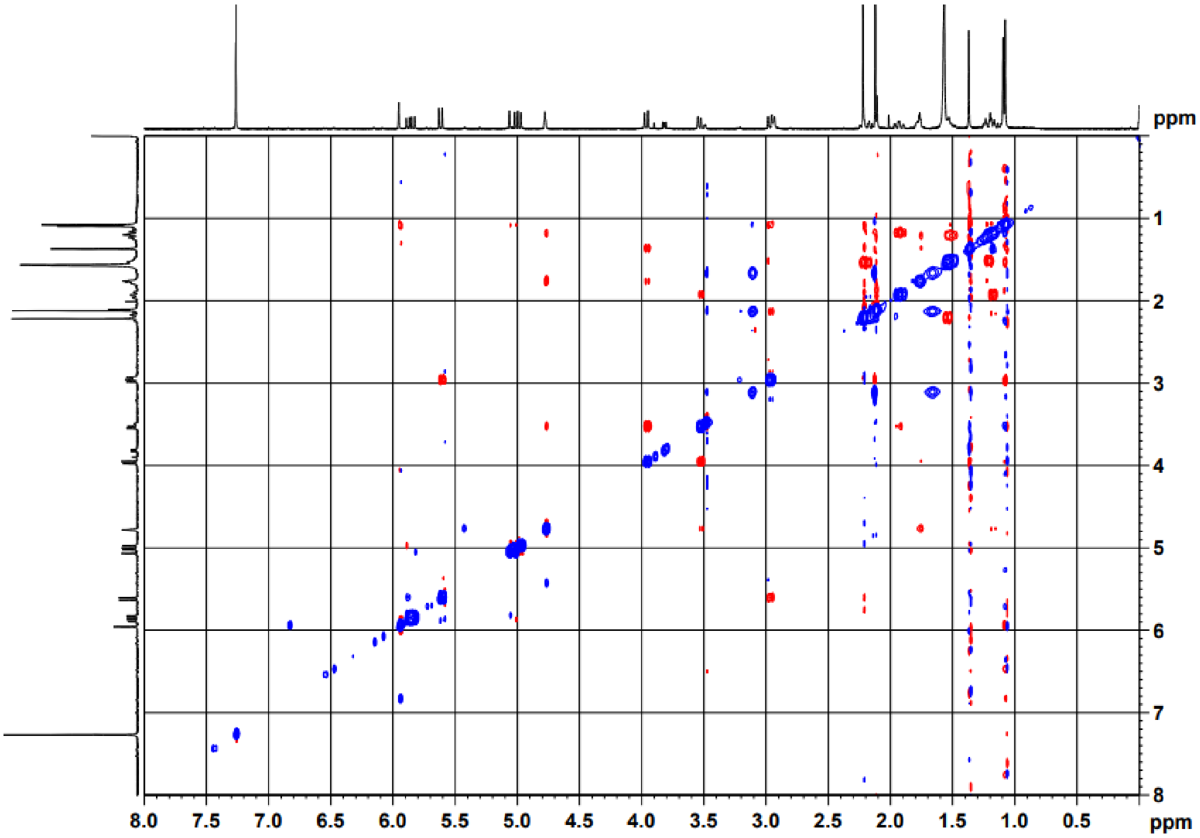

Figure S7 NOESY spectrum of compound **1** in $CDCl_3$

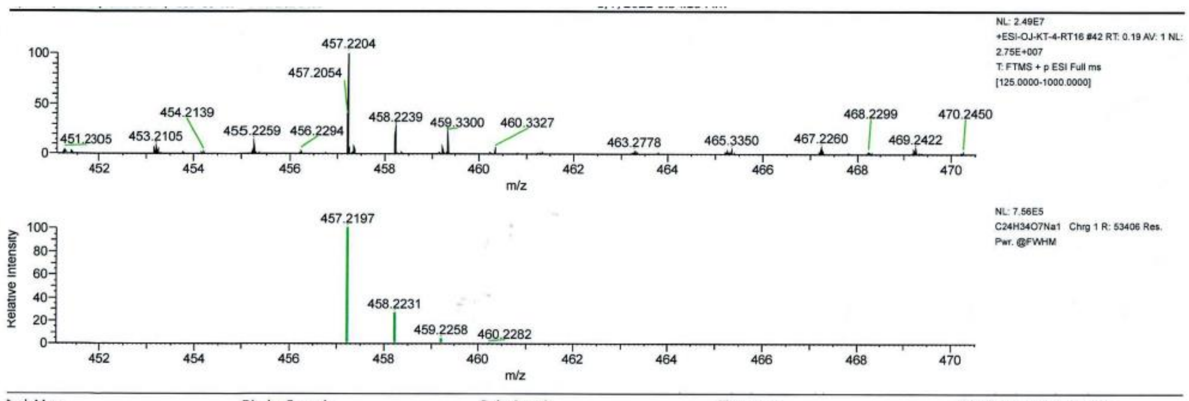

Peak Mass	Display Formula	Delta [ppm]	Theo. mass	MSMS Matched Fragments
457.2204	$C_{24}H_{34}O_7{}^{23}Na$ [M+Na]+	1.58	457.21967	(Collection)
457.2204	$C_{26}H_{33}O_7$	-3.68	457.22208	(Collection)
457.2204	$C_8H_{41}O_{20}$	4.00	457.21857	(Collection)

Figure S8 HREI (+) MS spectrum of compound **1**

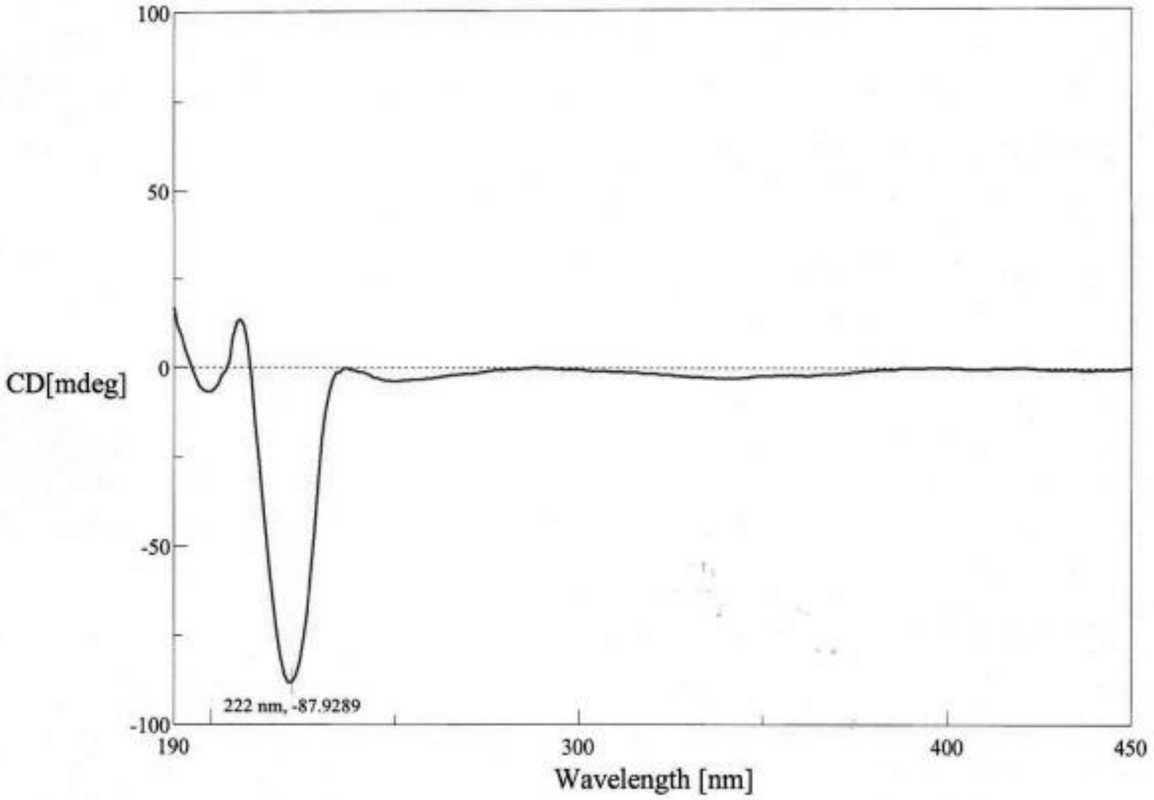

Figure S9 CD spectrum of compound **1**

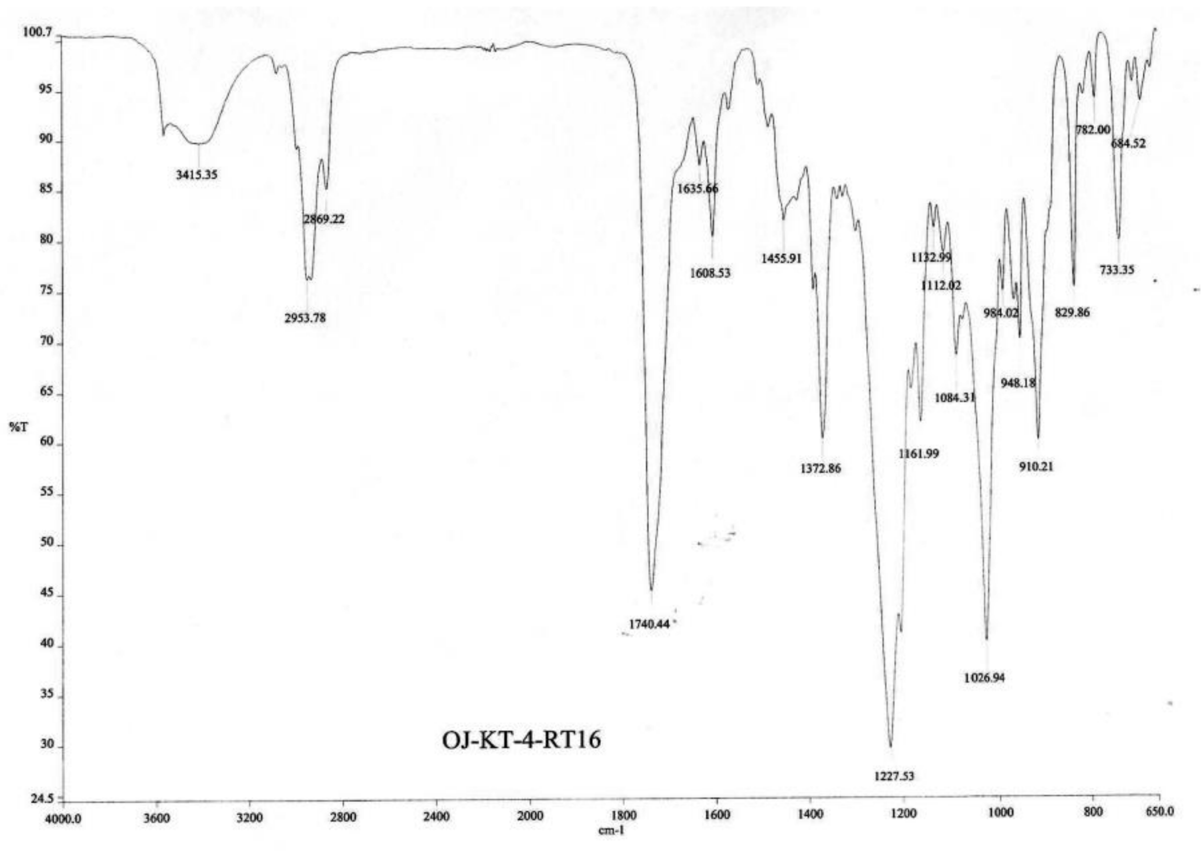

Figure S10 IR spectrum of compound **1**

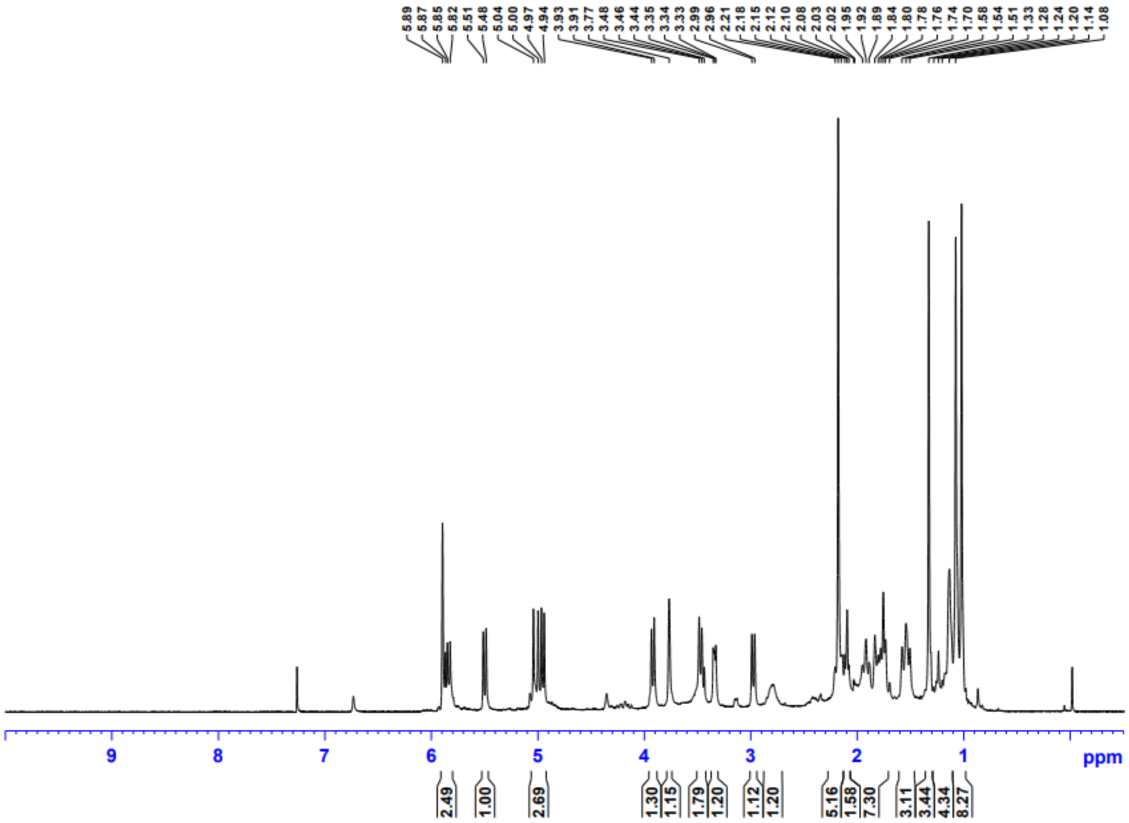

Figure S11 ^{1}H NMR spectrum (400 MHz) of compound **2** in $CDCl_3$

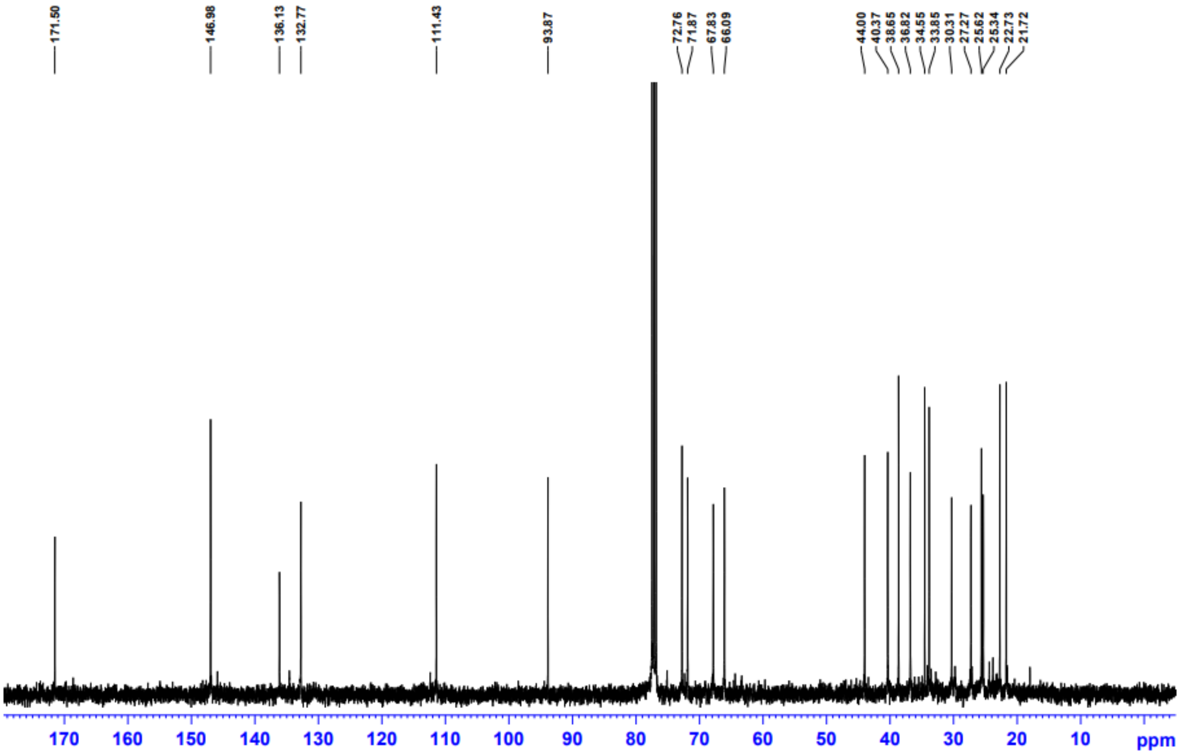

Figure S12 ^{13}C NMR spectrum (100 MHz) of compound **2** in $CDCl_3$

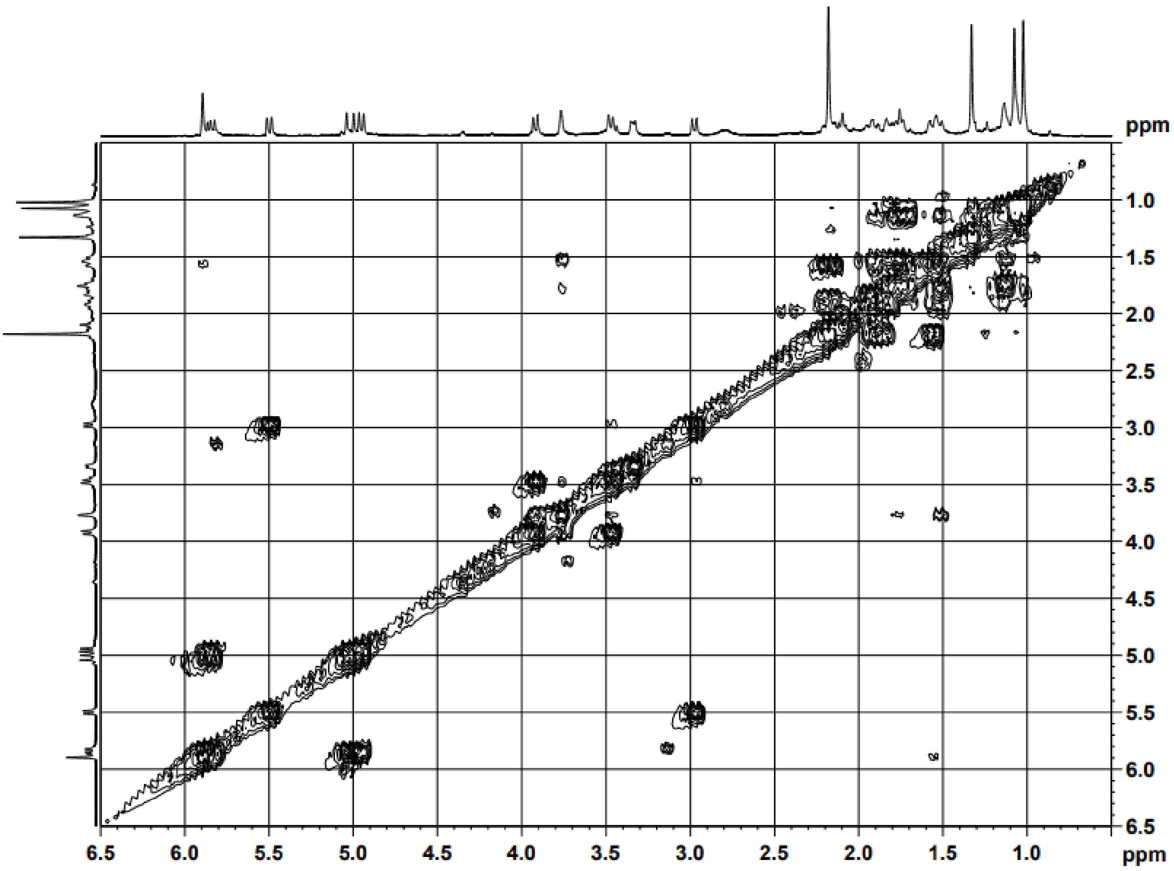

Figure S13 ^{1}H– ^{1}H COSY spectrum of compound **2** in $CDCl_3$

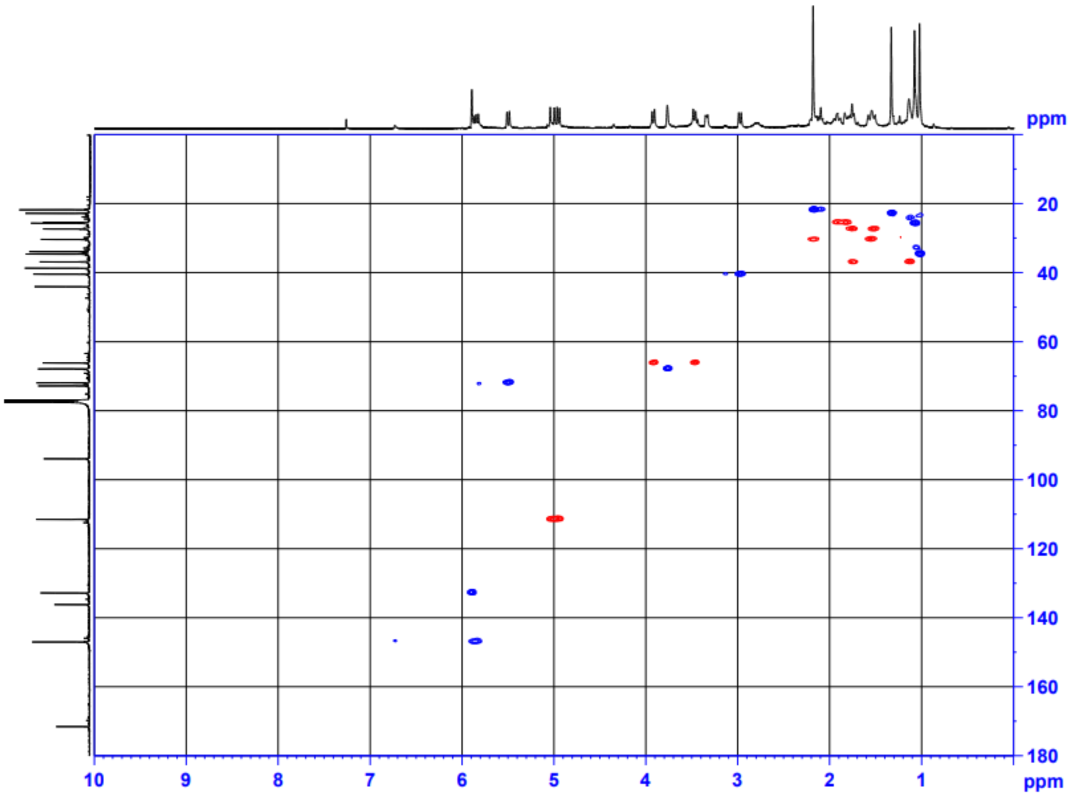

Figure S14 HSQC spectrum of compound **2** in $CDCl_3$

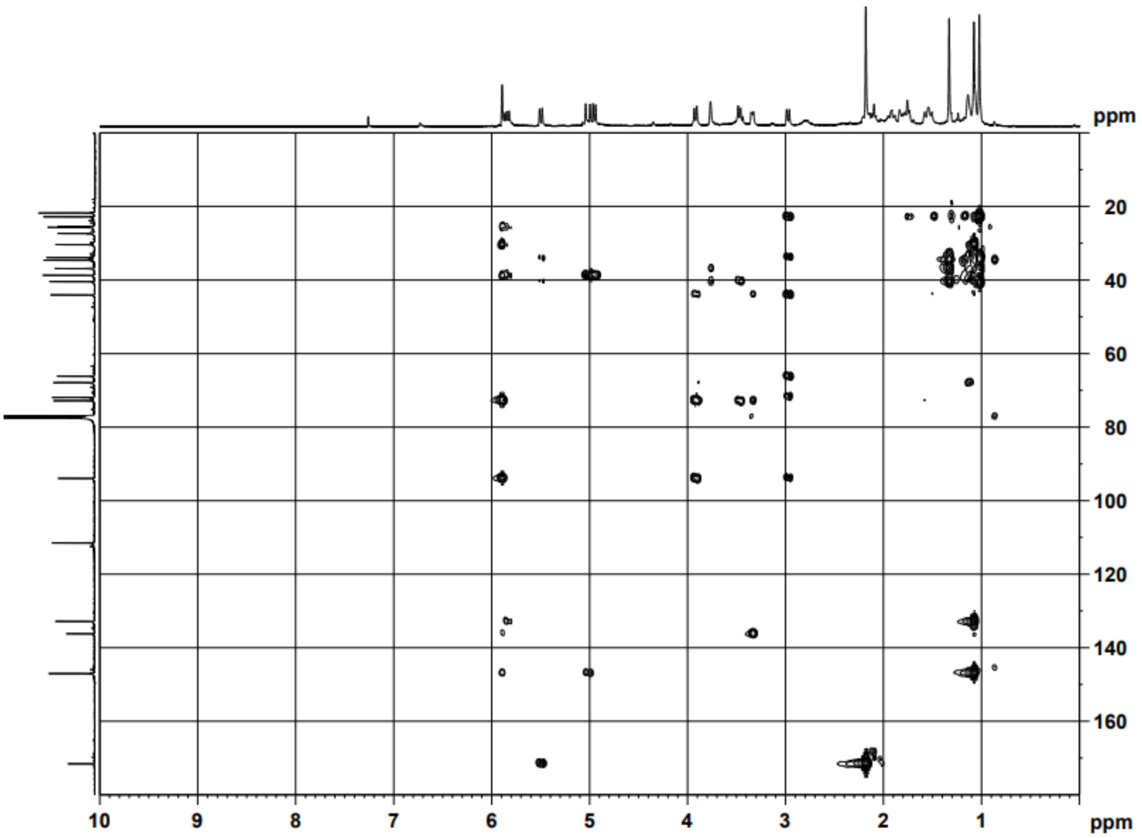

Figure S15 HMBC spectrum of compound **2** in $CDCl_3$

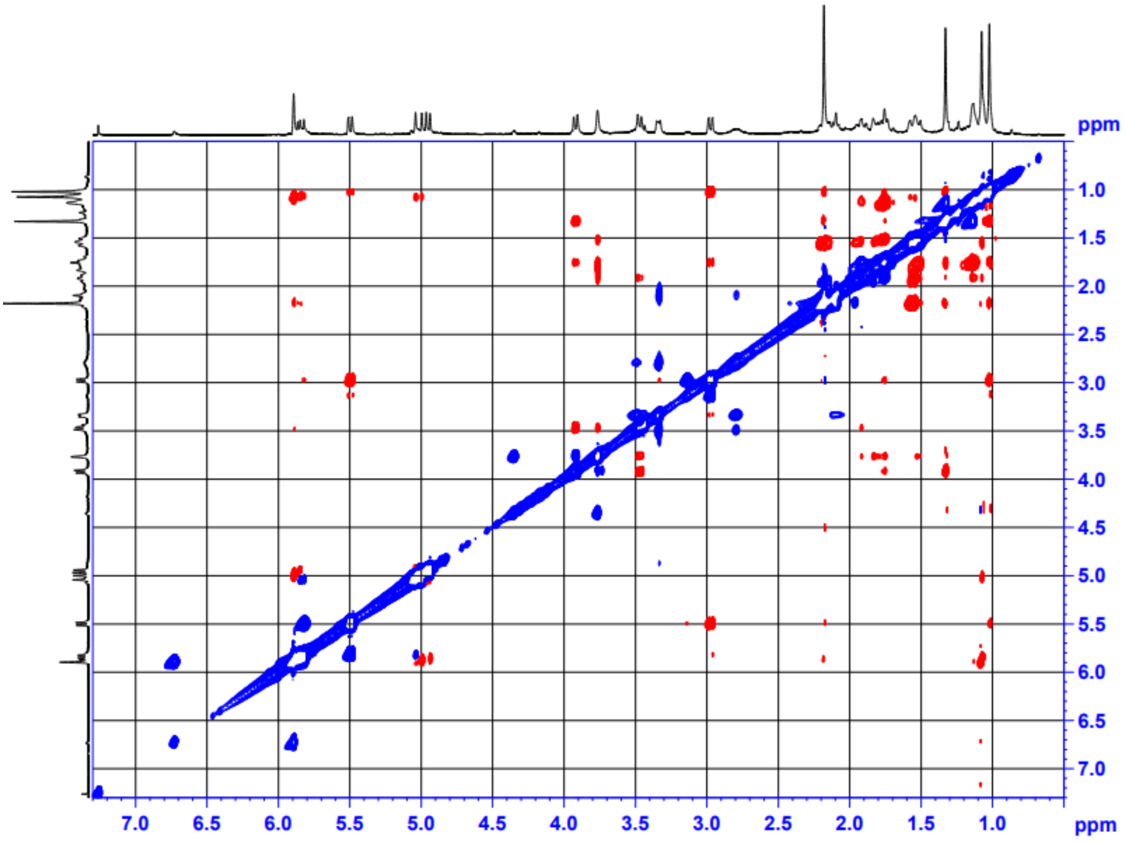

Figure S16 NOESY spectrum of compound **2** in $CDCl_3$

Acquisition Parameter

Source Type	ESI	Ion Polarity	Positive	Set Nebulizer	0.5 Bar
Focus	Not active	Set Capillary	3000 V	Set Dry Heater	125 °C
Scan Begin	60 m/z	Set End Plate Offset	-500 V	Set Dry Gas	5.5 l/min
Scan End	800 m/z	Set Charging Voltage	2000 V	Set Divert Valve	Source
		Set Corona	0 nA	Set APCI Heater	0 °C

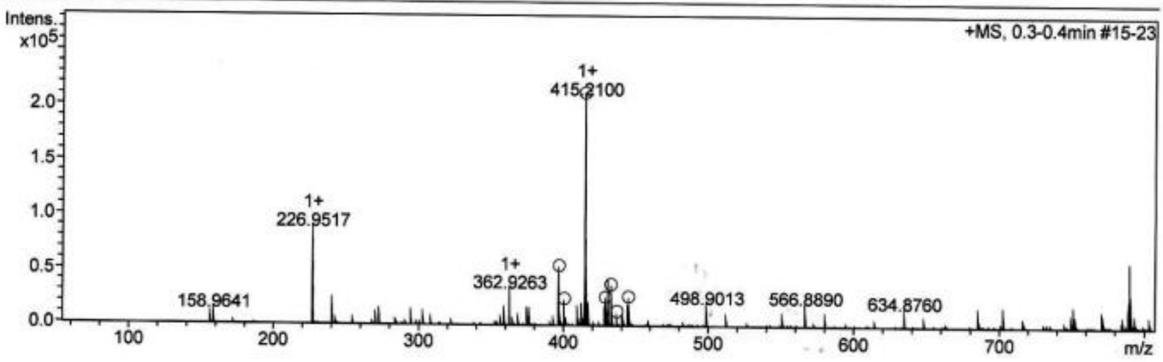

Meas. m/z	#	Ion Formula	m/z	err [ppm]	Mean err [ppm]	rdb	N-Rule	e⁻ Conf	mSigma	Std I	Std Mean m/z	Std I VarNorm	Std m/z Diff	Std Comb Dev
397.198815	1	C22H30NaO5	397.198545	-0.7	1039.3	7.5	ok	even	22.5	37.6	n.a.	n.a.	n.a.	n.a.
401.229712	1	C22H34NaO5	401.229845	0.3	1.6	5.5	ok	even	6.3	8.4	n.a.	n.a.	n.a.	n.a.
√ 415.209996	1	C22H32NaO6	415.209109	-2.1	-4.0	6.5	ok	even	24.3	37.9	n.a.	n.a.	n.a.	n.a.
429.225400	1	C23H34NaO6	429.224760	-1.5	962.2	6.5	ok	even	26.6	49.5	n.a.	n.a.	n.a.	n.a.
433.219949	1	C22H34NaO7	433.219674	-0.6	-0.2	5.5	ok	even	4.9	8.6	n.a.	n.a.	n.a.	n.a.
437.190255	1	C19H30N2NaO8	437.189437	-1.9	948.7	5.5	ok	even	22.7	40.6	n.a.	n.a.	n.a.	n.a.
445.219827	1	C23H34NaO7	445.219674	-0.3	-1.9	6.5	ok	even	30.7	49.1	n.a.	n.a.	n.a.	n.a.

Figure S17 HREI (+) MS spectrum of compound **2**

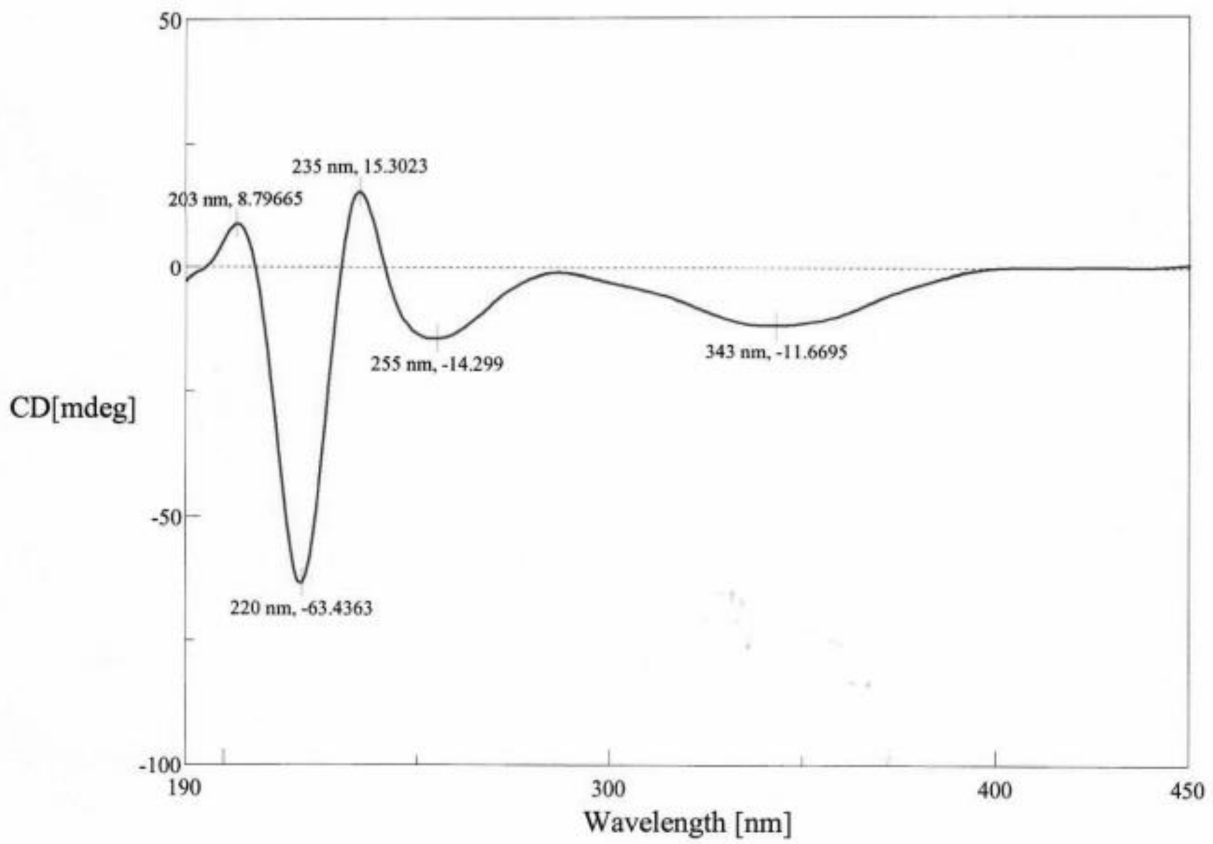

Figure S18 CD spectrum of compound **2**

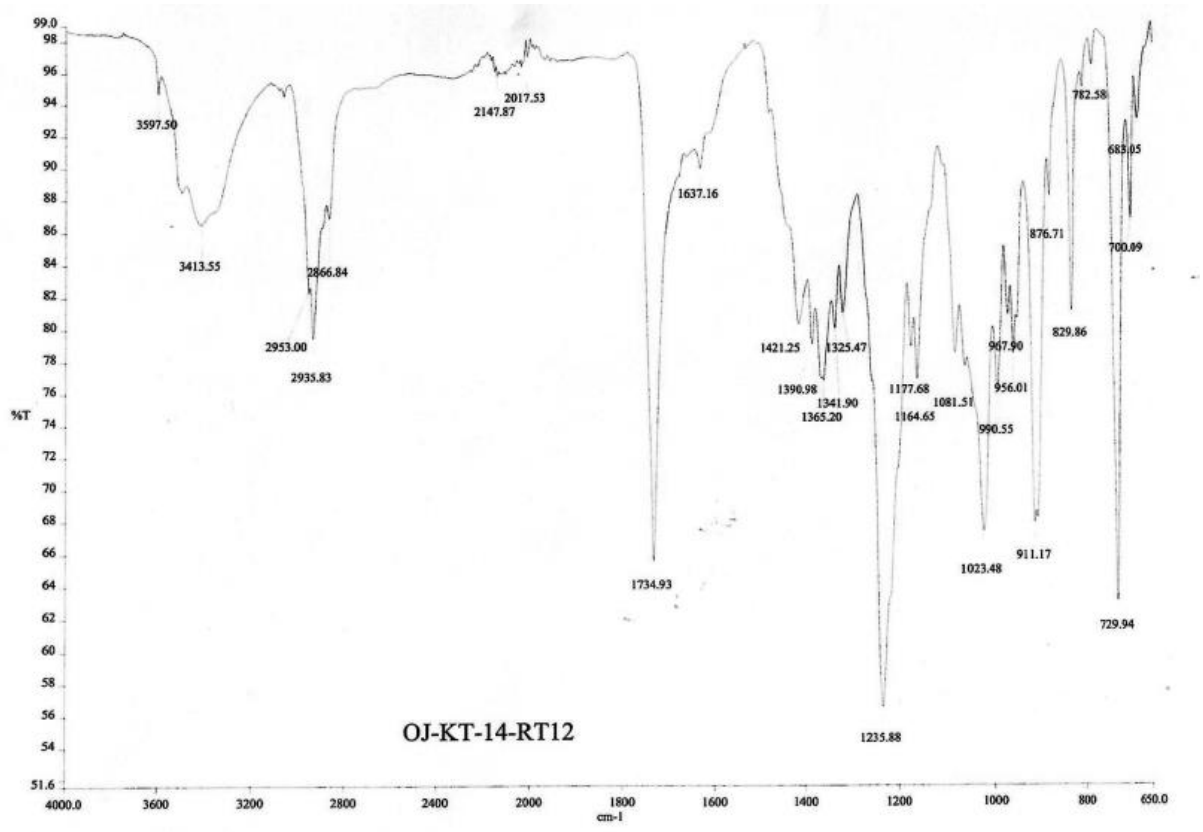

Figure S19 IR spectrum of compound **2**

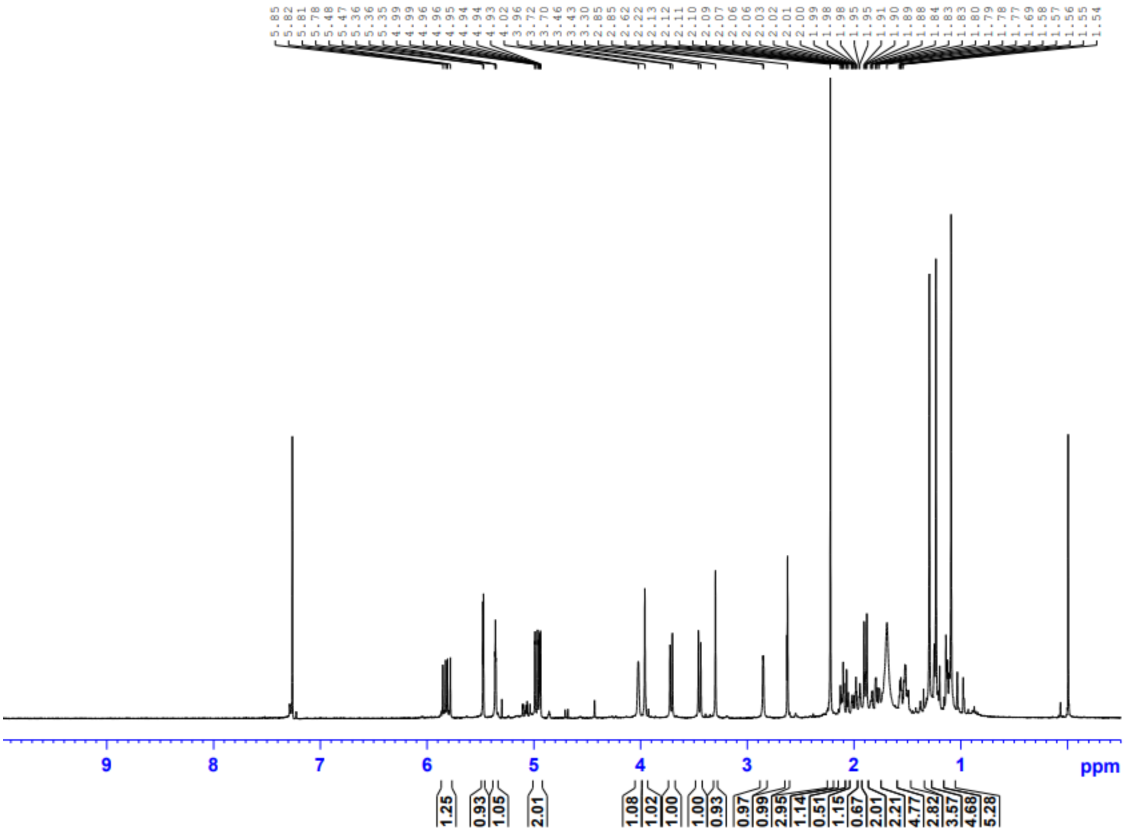

Figure S20 ^{1}H NMR spectrum (400 MHz) of compound **3** in $CDCl_3$

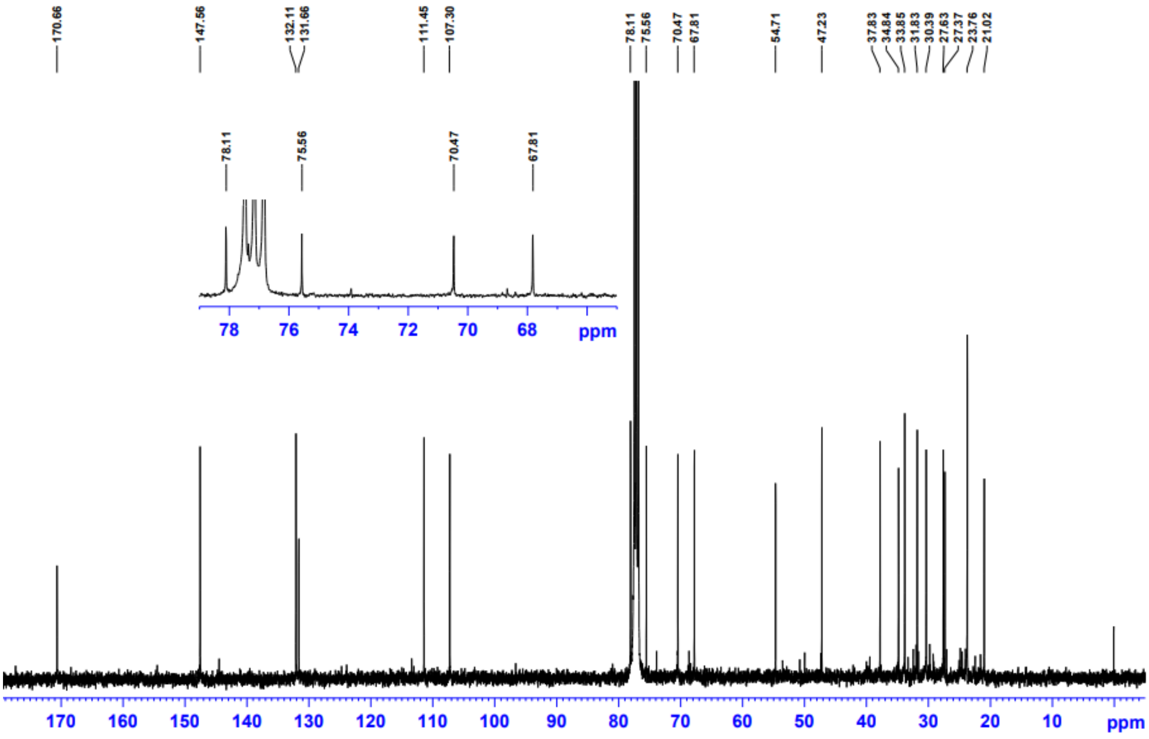

Figure S21 ^{13}C NMR spectrum (100 MHz) of compound **3** in $CDCl_3$

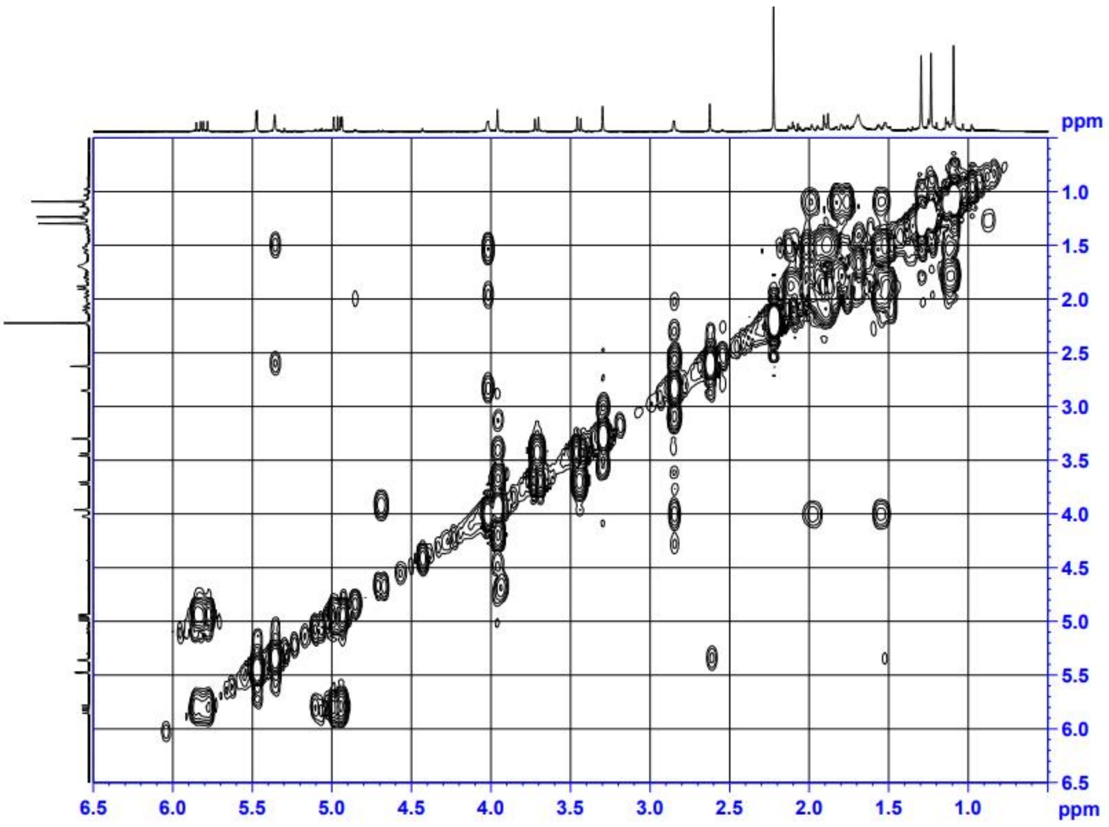

Figure S22 ^{1}H– ^{1}H COSY spectrum of compound **3** in $CDCl_3$

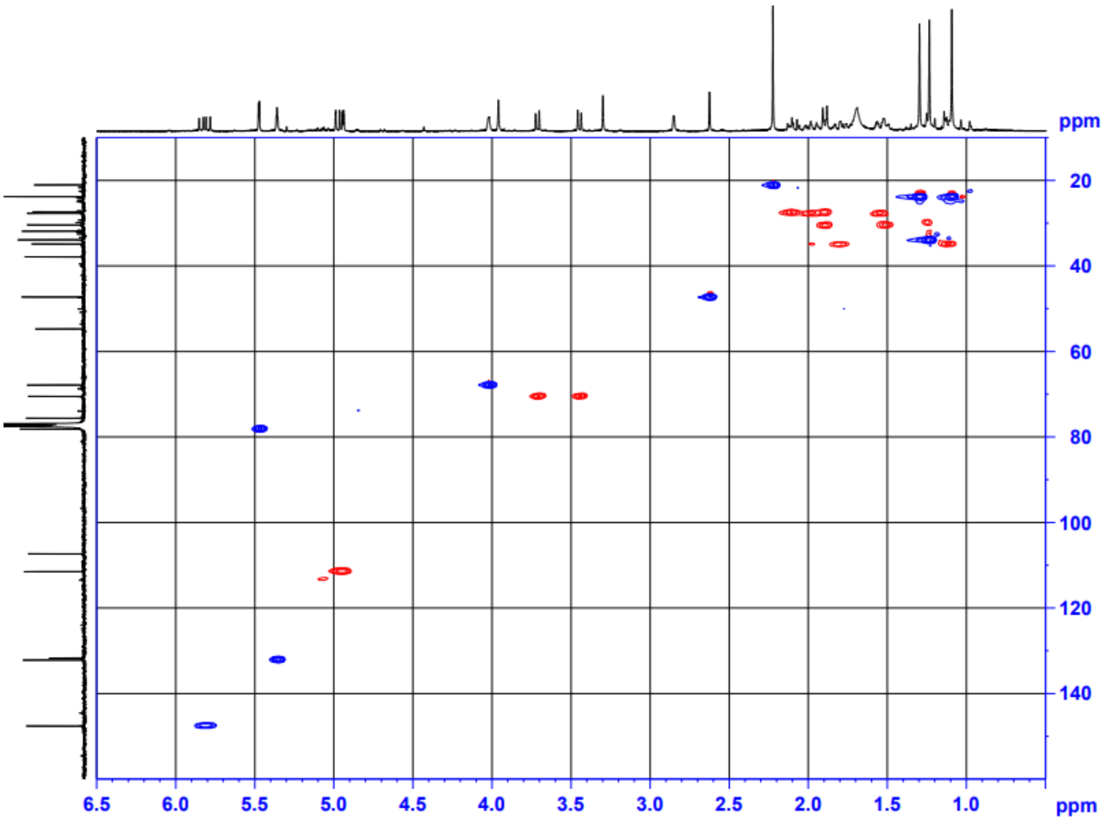

Figure S23 HSQC spectrum of compound **3** in $CDCl_3$

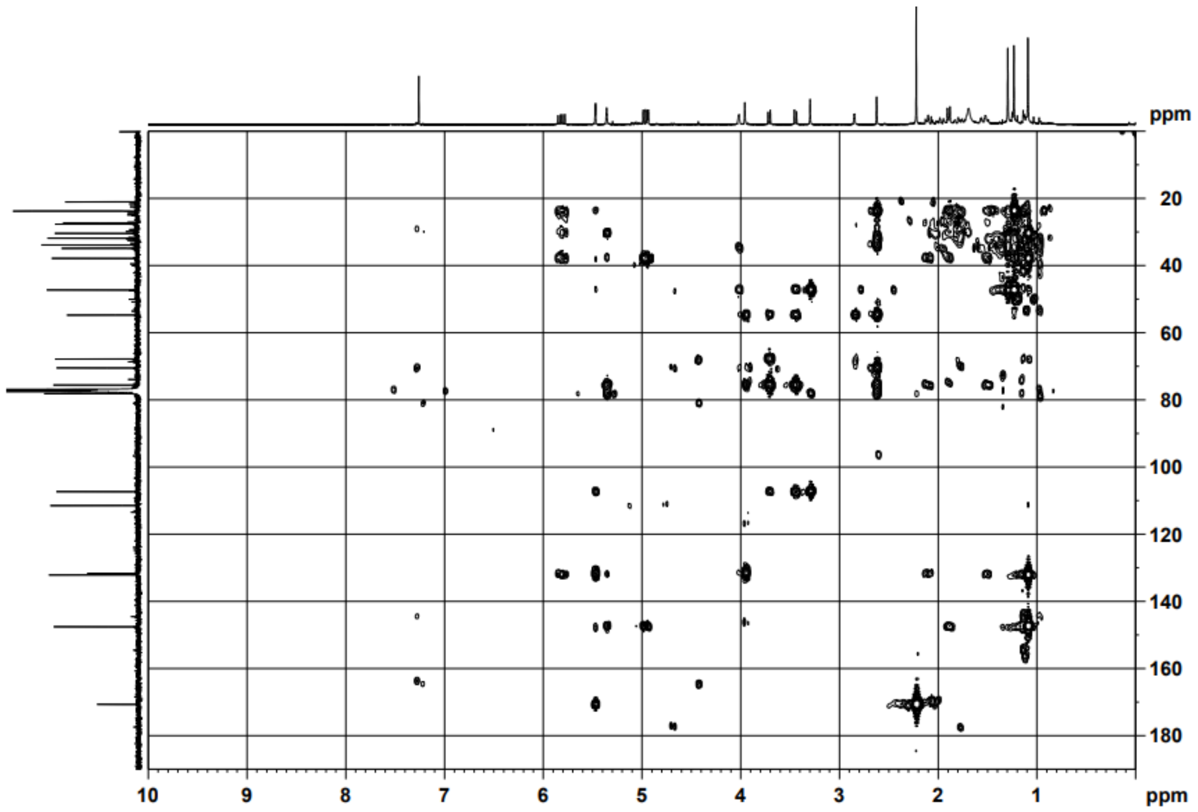

Figure S24 HMBC spectrum of compound **3** in $CDCl_3$

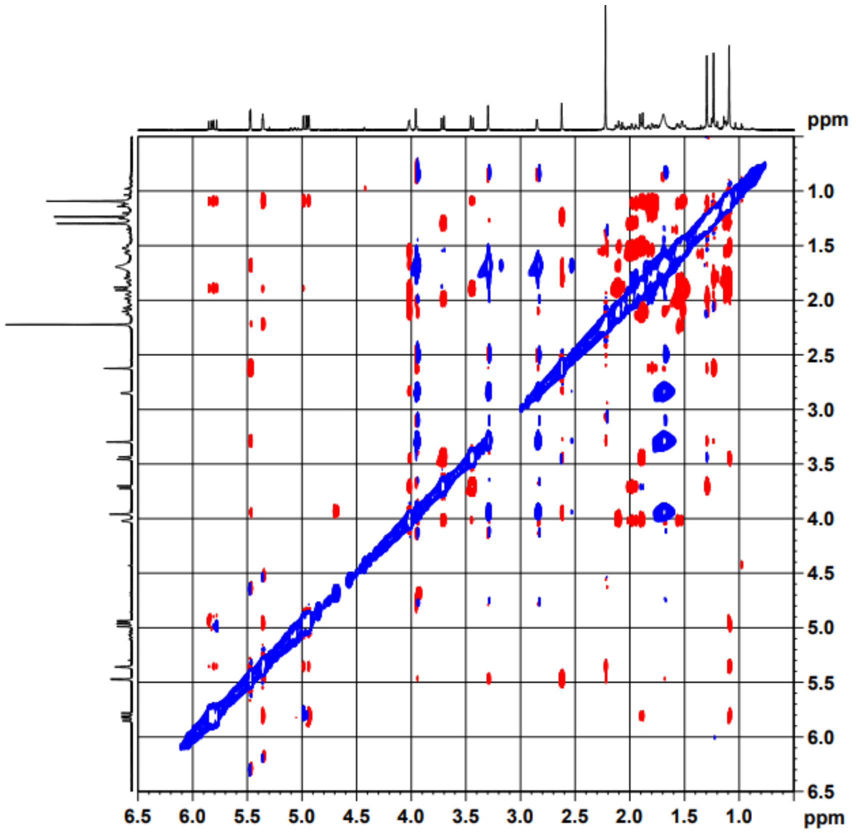

Figure S25 NOESY spectrum of compound **3** in $CDCl_3$

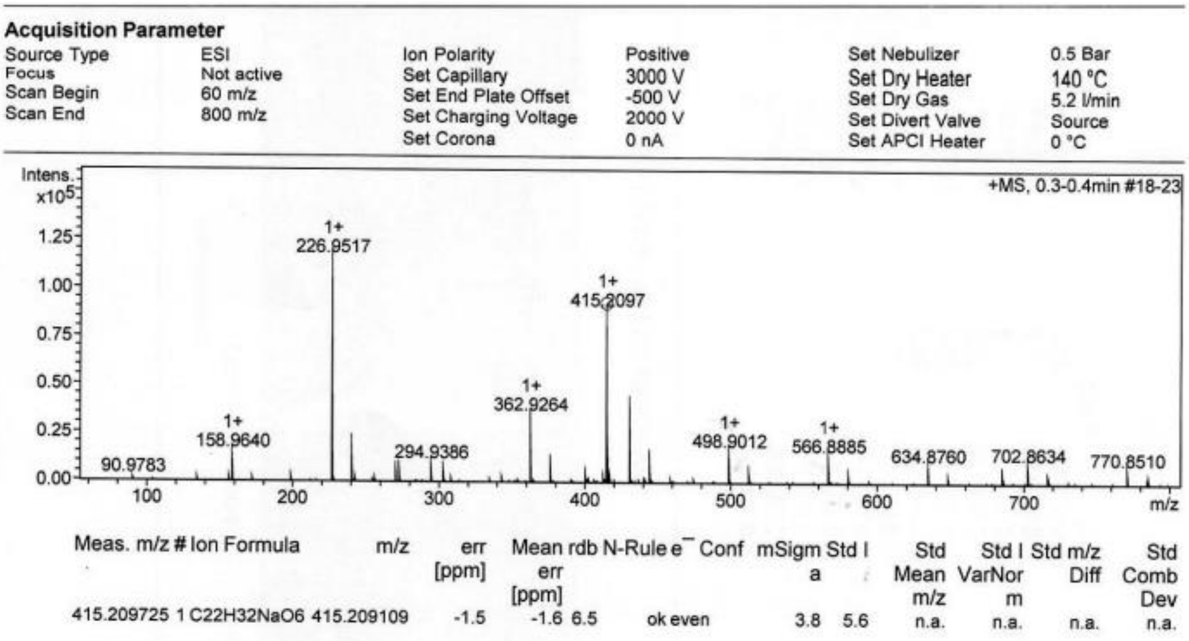

Figure S26 HREI (+) MS spectrum of compound **3**

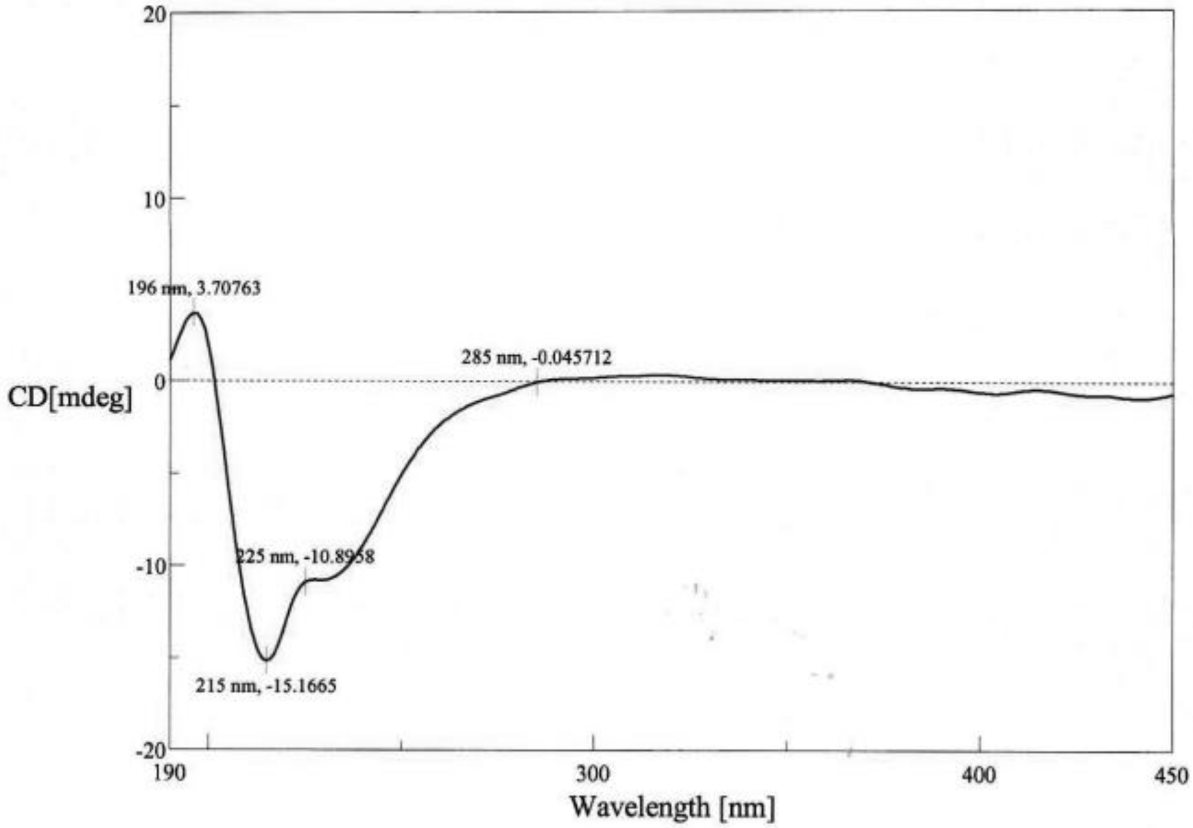

Figure S27 CD spectrum of compound **3**

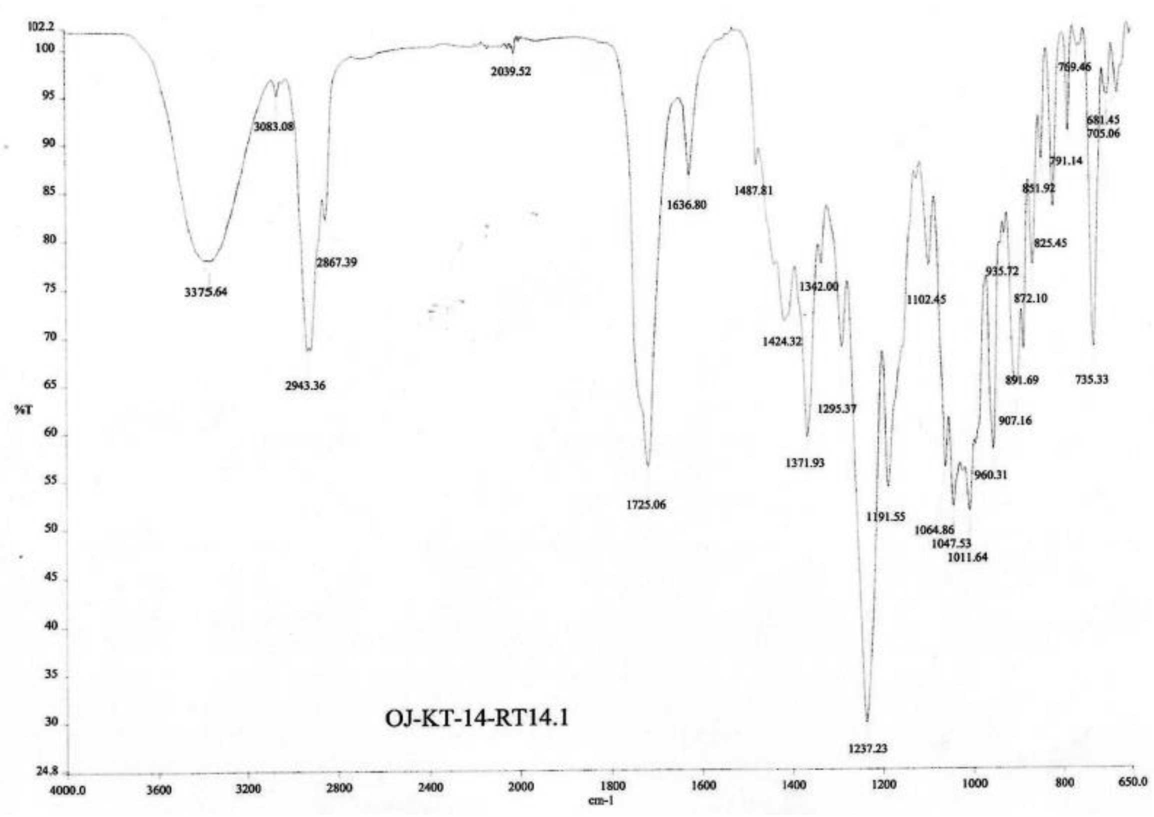

Figure S28 IR spectrum of compound **3**

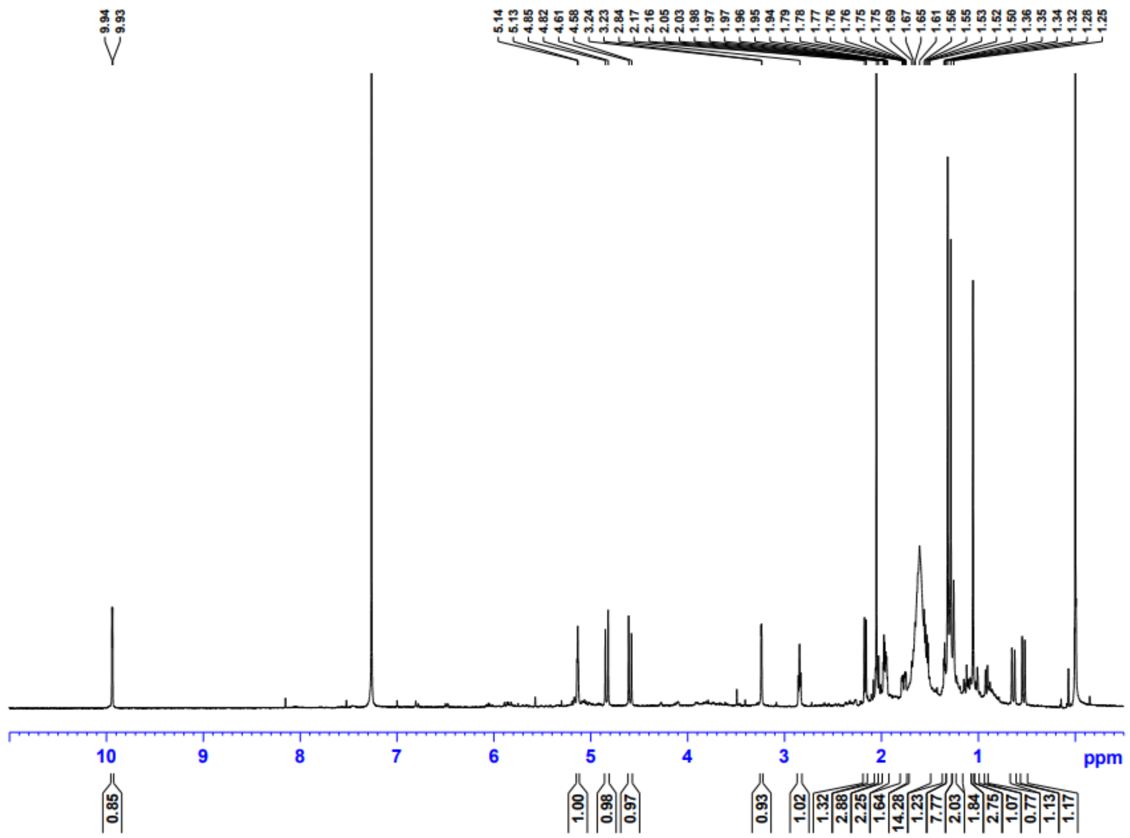

Figure S29 ^{1}H NMR spectrum (400 MHz) of compound **4** in $CDCl_3$

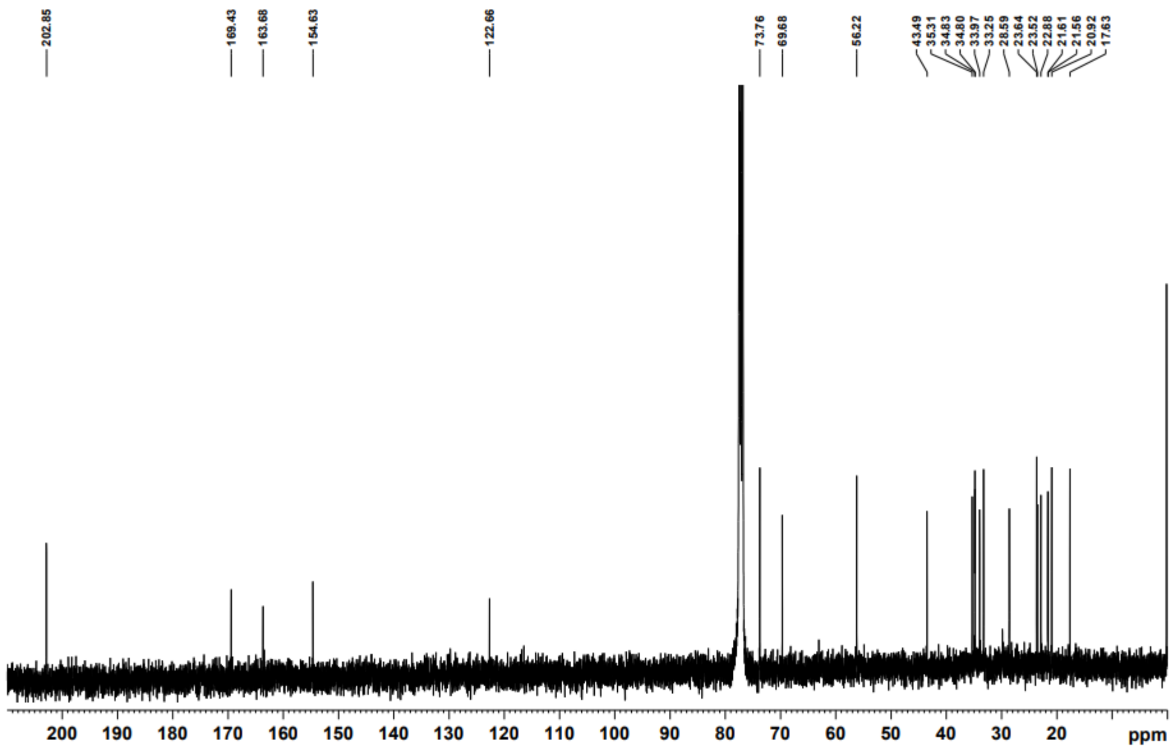

Figure S30 ^{13}C NMR spectrum (100 MHz) of compound **4** in $CDCl_3$

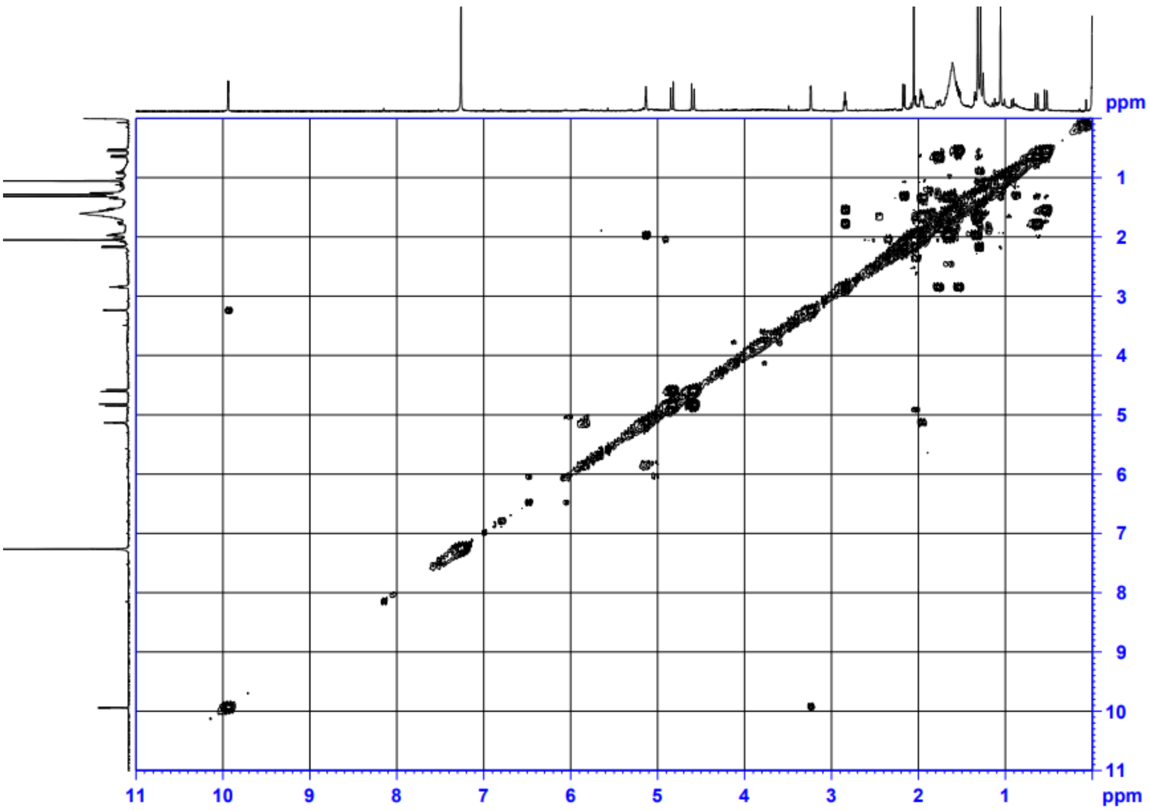

Figure S31 ^{1}H– ^{1}H COSY spectrum of compound **4** in $CDCl_3$

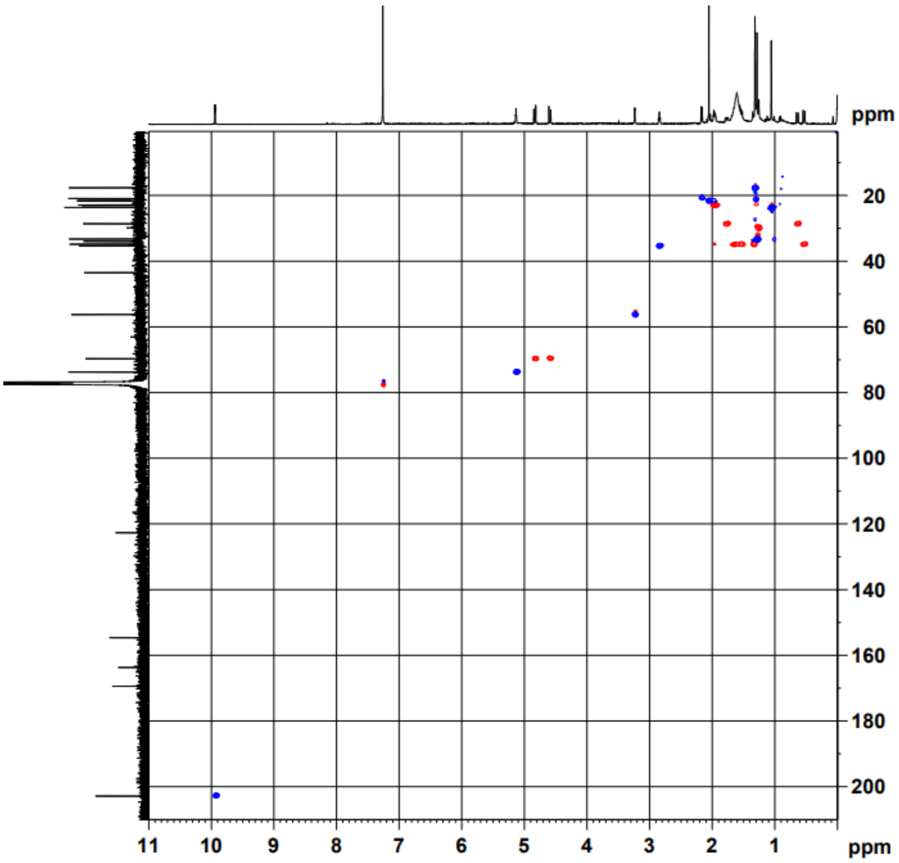

Figure S32 HSQC spectrum of compound **4** in $CDCl_3$

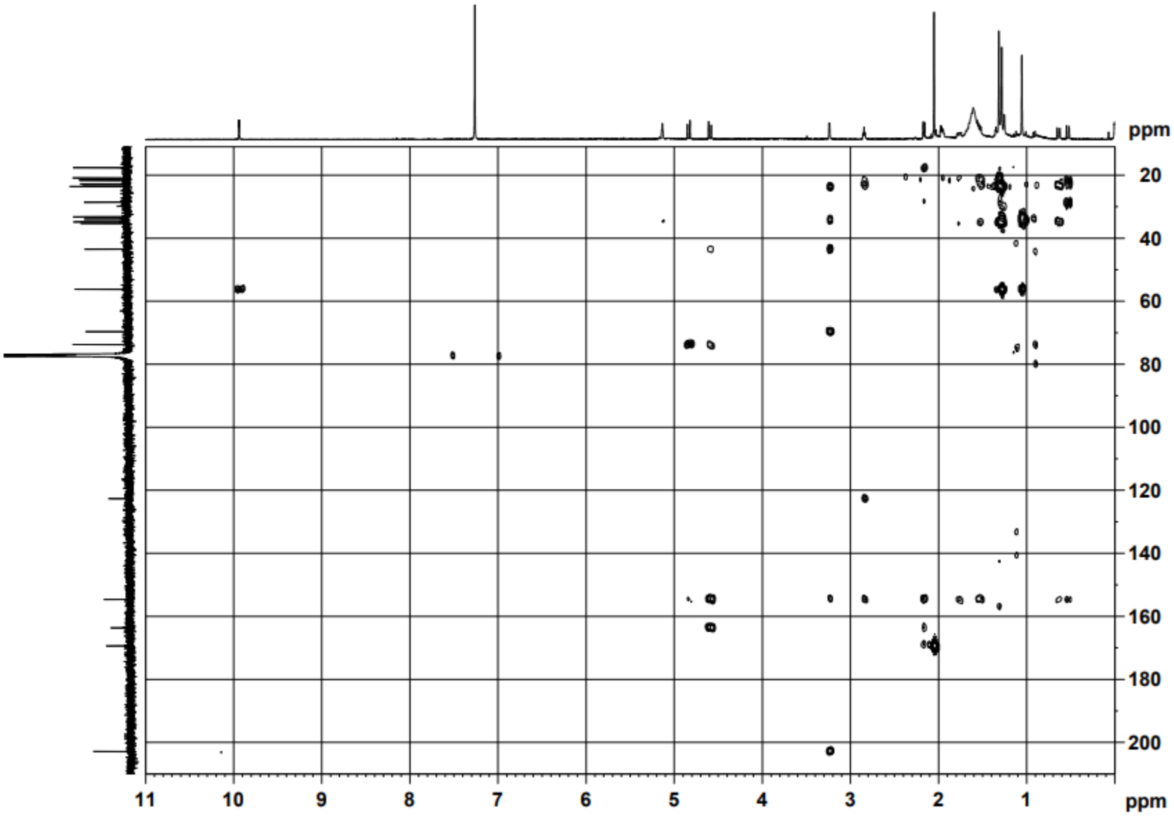

Figure S33 HMBC spectrum of compound **4** in $CDCl_3$

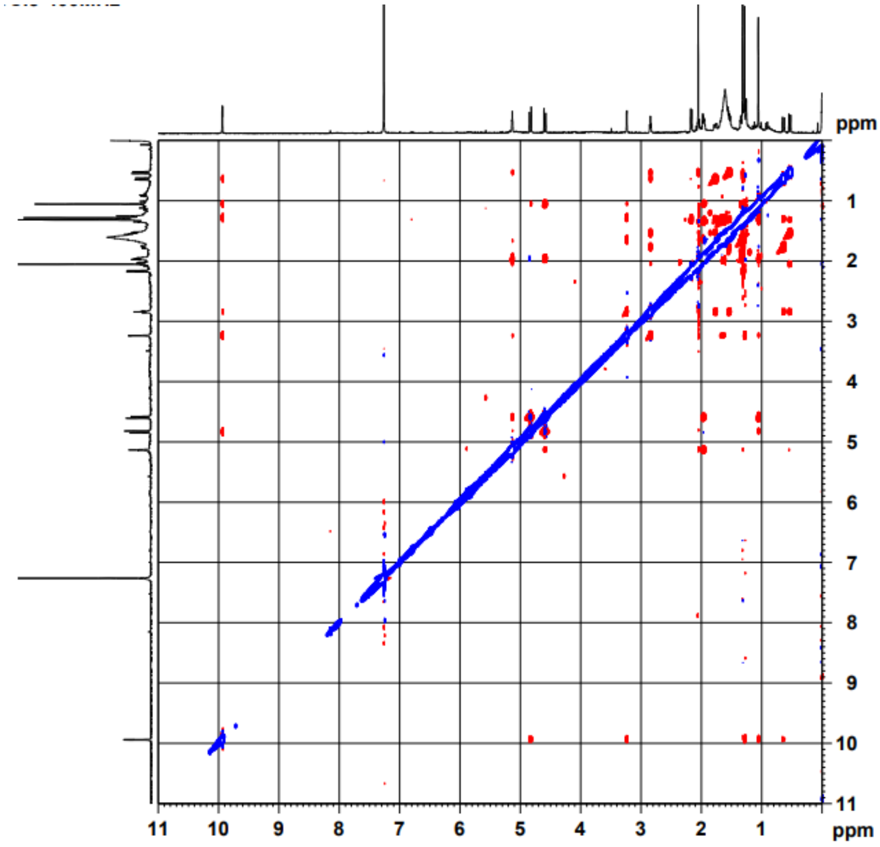

Figure S34 NOESY spectrum of compound **4** in $CDCl_3$

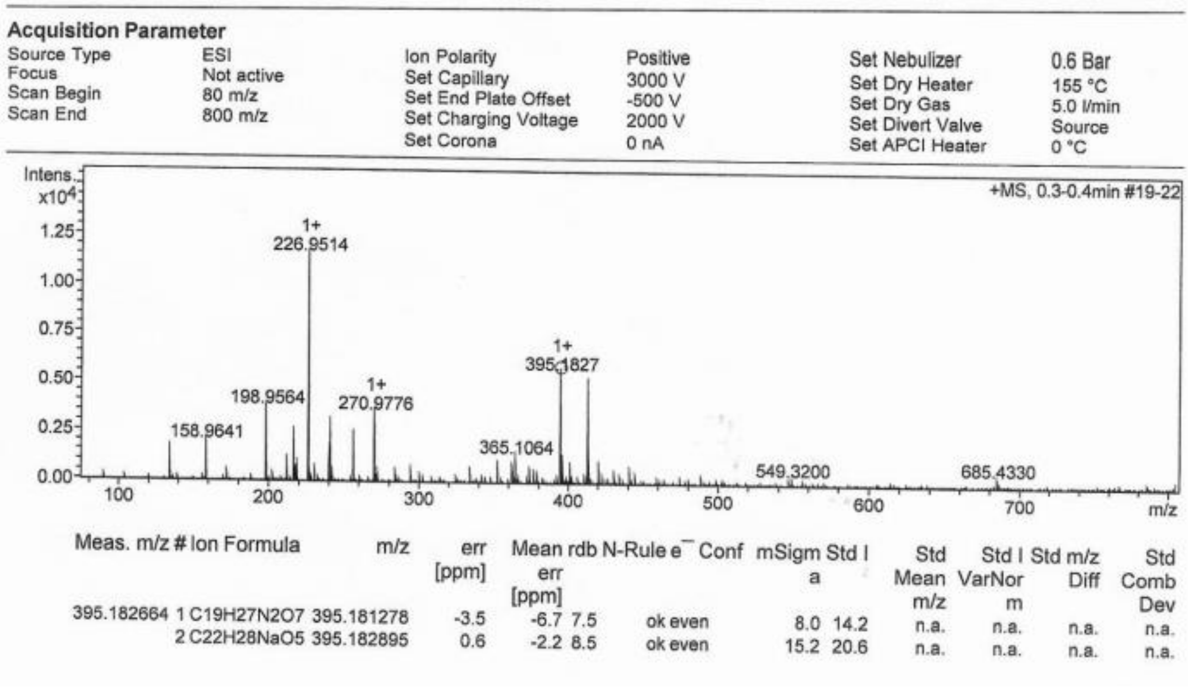

Figure S35 HREI (+) MS spectrum of compound **4**

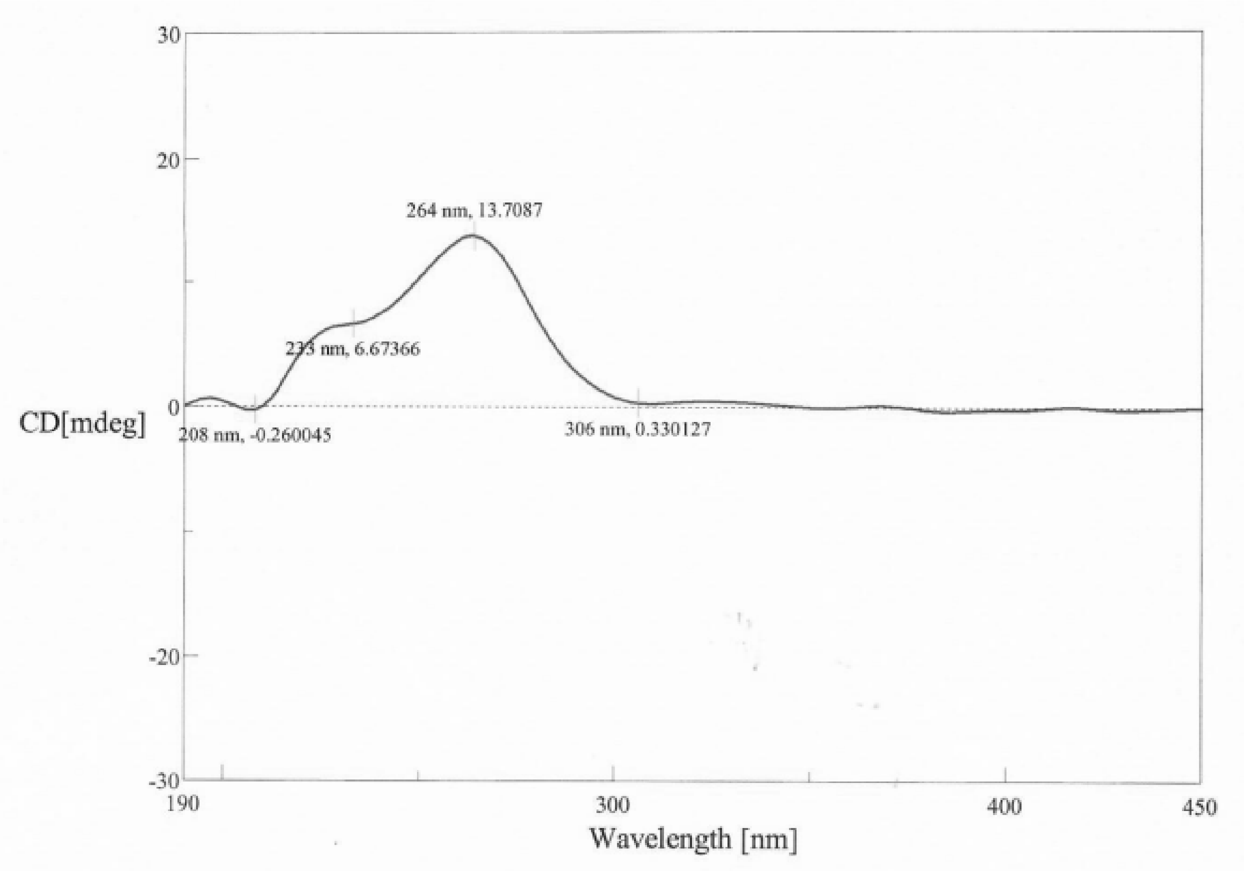

Figure S36 CD spectrum of compound **4**

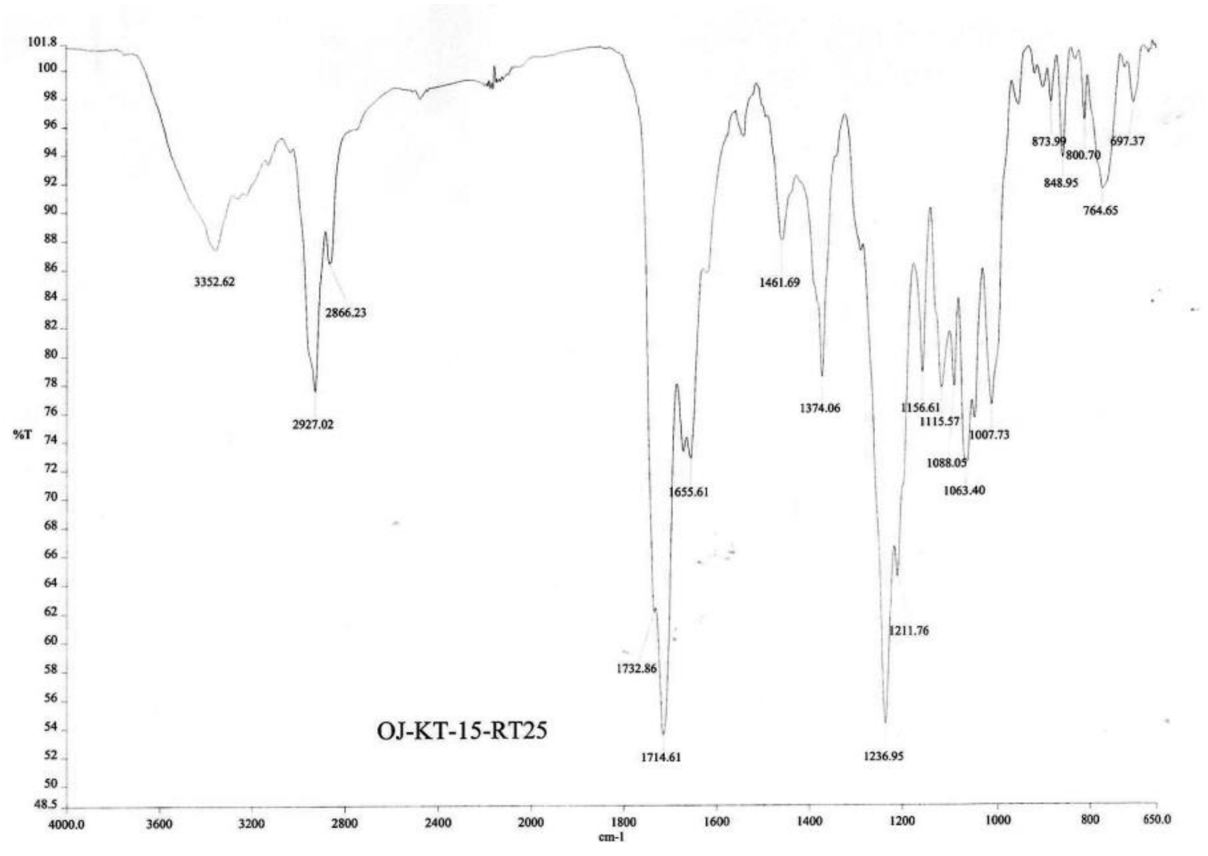

Figure S37 IR spectrum of compound **4**
